# Repairing Interfacial Defects in Self‐Assembled Monolayers for High‐Efficiency Perovskite Solar Cells and Organic Photovoltaics through the SAM@Pseudo‐Planar Monolayer Strategy

**DOI:** 10.1002/advs.202404725

**Published:** 2024-07-30

**Authors:** Chieh‐Ming Hung, Chi‐Chi Wu, Yu‐Hsuan Yang, Bo‐Han Chen, Chih‐Hsuan Lu, Che‐Chun Chu, Chun‐Hao Cheng, Chun‐Yun Yang, Yan‐Ding Lin, Ching‐Hsuan Cheng, Jiann‐Yeu Chen, I‐Chih Ni, Chih‐I Wu, Shang‐Da Yang, Hsieh‐Chih Chen, Pi‐Tai Chou

**Affiliations:** ^1^ Department of Chemistry Center for Emerging Materials and Advanced Devices National Taiwan University Taipei 106319 Taiwan; ^2^ Institute of Photonics Technologies National Tsing Hua University Hsinchu 300044 Taiwan; ^3^ Department of Fiber and Composite Materials Feng Chia University Taichung 407802 Taiwan; ^4^ i‐Center for Advanced Science and Technology (i‐CAST) and Innovation and Development Center of Sustainable Agriculture (IDCSA) National Chung Hsing University Taichung 402202 Taiwan; ^5^ Graduate Institute of Photonics and Optoelectronics National Taiwan University Taipei 106319 Taiwan; ^6^ Department of Chemistry Fu Jen Catholic University New Taipei City 242062 Taiwan

**Keywords:** hole‐selective layer, perovskite solar cells, pseudo‐planar monolayer, self‐assembled monolayer, transient absorption spectroscopy

## Abstract

Lately, carbazole‐based self‐assembled monolayers (SAMs) are widely employed as effective hole‐selective layers (HSLs) in inverted perovskite solar cells (PSCs). Nevertheless, these SAMs tend to aggregate in solvents due to their amphiphilic nature, hindering the formation of a monolayer on the ITO substrate and impeding effective passivation of deep defects in the perovskites. In this study, a series of new SAMs including DPA‐B‐PY, CBZ‐B‐PY, POZ‐B‐PY, POZ‐PY, POZ‐T‐PY, and POZ‐BT‐PY are synthesized, which are employed as interfacial repairers and coated atop CNph SAM to form a robust CNph SAM@pseudo‐planar monolayer as HSL in efficient inverted PSCs. The CNph SAM@pseudo‐planar monolayer strategy enables a well‐aligned interface with perovskites, synergistically promoting perovskite crystal growth, improving charge extraction/transport, and minimizing nonradiative interfacial recombination loss. As a result, the POZ‐BT‐PY‐modified PSC realizes an impressively enhanced solar efficiency of up to 24.45% together with a fill factor of 82.63%. Furthermore, a wide bandgap PSC achieving over 19% efficiency. Upon treatment with the CNph SAM@pseudo‐planar monolayer, also demonstrates a non‐fullerene organic photovoltaics (OPVs) based on the PM6:BTP‐eC9 blend, which achieves an efficiency of 17.07%. Importantly, these modified PSCs and OPVs all show remarkably improved stability under various testing conditions compared to their control counterparts.

## Graphical abstract

A new series of SAMs from DPA‐B‐PY to POZ‐BT‐PY, employed as interfacial repairers, are coated atop CNph SAM to form a robust CNph SAM@pseudo‐planar monolayer as HSL in inverted PSCs. The CNph SAM@pseudo‐planar monolayer strategy enables a well‐aligned interface with perovskites, synergistically promoting perovskite crystal growth, improving charge extraction/transport, and minimizing nonradiative interfacial recombination loss.

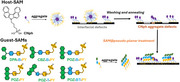

## 1 Introduction

Over the past decade, emerging perovskite solar cells (PSCs) have garnered significant attention as one of the most promising photovoltaic technologies. This is evidenced by a remarkable increase in power conversion efficiency (PCE), rising from 3.8% to a certified 26.1%, placing them on par with crystalline silicon solar cells.^[^ ^1^ ^]^ Inverted PSCs offer attractive advantages compared to regular PSCs, including high device stability, negligible hysteresis, and excellent compatibility with flexible and tandem cells.^[^ ^2^ ^]^ In this regard, ultrathin self‐assembled monolayers (SAMs) have been considered as one of the most promising hole‐selective layers (HSLs) for inverted PSCs due to their low costs, minimal material consumption, and simple device fabrication process, while maintaining high PCEs.^[^ ^3^ ^]^

Employment of SAM as an HSL in inverted PSCs offers multiple unique properties. Firstly, SAM molecules can be easily modified by altering functional groups, enabling precise control over the substrate surface properties. Secondly, SAM forms a thin and compact layer between the perovskite and ITO, facilitating hole transfer from the perovskite to Indium Tin Oxide ITO via SAM and preventing back charge recombination. Thirdly, SAMs exhibit low material consumption and minimal parasitic absorption in the device. Fourthly, SAMs are soluble in green solvents such as ethanol, reducing biotoxicity. Typically, these SAMs incorporate anchoring groups such as carboxylic acid or phosphonic acid, which promote the formation of covalent bonds to the substrate, ensuring uniform coverage of the SAMs. To date, most of efficient SAMs are based on carbazole core renowned for their electron‐rich nature and exceptional hole transport capability, forming passivated interfaces with perovskites, effectively reducing charge carrier transport losses and enhancing device performance.^[^ ^4^ ^]^ Albrecht et al. successfully improved the interface by employing carbazole‐containing SAMs of 2PACz and MeO‐2PACz as HSLs for inverted PSCs. This approach established a hole‐selective contact, reducing interface recombination and aiding hole extraction. Consequently, they attained impressive PCEs of 20.9% and 21.2% for 2PACz‐ and MeO‐2PACz SAM‐based PSCs, respectively.^[^ ^5^ ^]^ Jen et al. employed CbzNaph (CNph) SAM as an efficient HSL for inverted PSCs. They enhanced its molecular dipole moment and strengthened π–π interactions through helical π‐expansion, resulting in an impressive PCE of 24.1% and improved device stability.^[^ ^6^ ^]^ Regardless of the impressive progress in developing SAMs for inverted PSCs, there are still some critical shortcomings that need to be addressed. The steric hindrance of the carbazole‐based SAMs, such as 2PACz, MeO‐2PACz, and CbzNaph (CNph), tends to aggregate in solvents owing to their amphiphilic nature, hampering formation of a monolayer on the ITO substrate, obstructing efficacious passivation of deep defects in the perovskites. Therefore, it may give rise to the energy loss and nonradiative recombination, which is disadvantageous for the device performance. Jen et al. introduced a co‐solvent approach to effectively disperse precursors and mitigate interface traps. Moreover, addressing permanent defects on the ITO surface necessitates an additional anchoring group. Most SAMs utilized so far are based on carbazole derivatives, lacking efficient electron‐withdrawing capabilities. This limitation hinders charge dynamics, requiring a larger driving force for exciton dissociation and charge extraction. To overcome this hurdle, we introduced molecules designed to interact with ITO. This effectively fills interfacial deep traps, passivates perovskite defects, and simultaneously enhances exciton dissociation probability and charge extraction capability. Additionally, long‐term stability is another significant challenge toward commercialization. Intrinsic ion migration and poor charge‐selective contact interfaces severely hamper device performance and hasten the degradation process. This has spurred efforts to find robust HSLs capable of passivating interfacial defects while enabling effective charge transfer.

**Figure 1:**
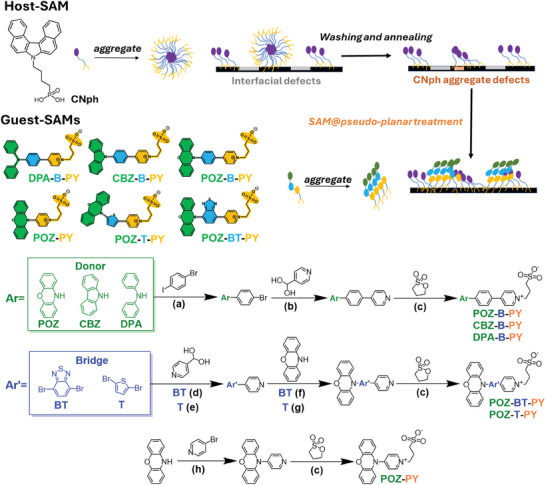
A schematic representation of SAM@pseudo‐planar monolayer treatment together with the chemical structures employed in this study. The synthetic route to zwitterions salts. a) Pd(OAc)_2_, xantphos, NaO*^t^* Bu, toluene, 110 °C, 16 h. b) Pd(PPH_3_)_2_Cl_2_, K_2_CO_3_, toluene/EtOH/H_2_O, 110 °C, 16 h. c) 1,3‐Propanesultone (Ps), toluene, 110 °C, 48 h. d) Pd(PPH_3_)_4_, K_2_CO_3_,1,4‐dioxane/H_2_O, 110 °C, 16 h. e) Pd(PPH_3_)_2_Cl_2_, K_2_CO_3_, toluene/EtOH/H_2_O, 130 °C, 2.5 h. f) Pd(OAc)_2_, [(*^t^* Bu)_3_P]HBF_4_, NaO*^t^* Bu, toluene, 110 °C, 72 h. g) Pd_2_(dba)_3_, [(*^t^* Bu)_3_P]HBF_4_, NaO*^t^* Bu, xylene, 115 °C, 16 h. h) Pd(OAc)_2_, xantphos, K_2_CO_3_, toluene, 110 °C, 16 h.

Inspired by the aforementioned perspectives, we report, for the first time, a series of sulfonate‐based D–π–A SAMs, including DPA‐B‐PY, CBZ‐B‐PY, POZ‐B‐PY, POZ‐PY, POZ‐T‐PY, and POZ‐BT‐PY (see **Figure** **1**) as potential solutions to the interfacial challenges in SAM/perovskite heterojunctions. These SAMs demonstrate promising potential as interfacial repairers for the HSL in efficient inverted PSCs. Particularly noteworthy is the incorporation of these sulfonate‐based D–π–A SAMs, coated atop CNph SAM via a two‐step approach, resulting in a robust CNph SAM@pseudo‐planar monolayer (Figure 1). This configuration features an appropriate energy level cascade, which enhances charge transfer as well as renders effective passivation of buried interfacial defects. The net effect is to improve monolayer coverage, promote perovskite crystal growth, reduce interfacial nonradiative recombination losses, and hence facilitate the hole extraction.

Herein, we conduct a comprehensive study into the interplays of sulfonate‐based SAMs, CNph SAM, and perovskites by integrating theoretical calculations with experimental studies. The champion device modified with POZ‐BT‐PY achieves a PCE of 24.45%, consistently higher than that of the reference CNph device (23.00%). In addition, devices modified with POZ‐BT‐PY exhibit much improved long‐term operational stability under ambient and thermal conditions, compared to the reference CNph device. Extending this approach to the wide bandgap (1.73 eV) inverted PSCs yields a maximum PCE surpassing 19%, along with a *V*_OC_ of 1.300 V and a minimal voltage loss of 0.43 V. This achievement holds great promise for the application of perovskite/silicon tandem solar cells. Additionally, applying SAM@pseudo‐planar monolayer treatment to the non‐fullerene organic photovoltaics (OPVs) based on the PM6:BTP‐eC9 system also boosts its solar efficiencies from 16.28% to 17.07%, with notable improvements in short‐circuit current density (*J*_SC_) and fill factor (FF). The SAM@pseudo‐planar monolayer strategy offers a simple and versatile solution to address interfacial issues in SAM/perovskite heterojunctions, applicable to both PSCs and OPVs.

## 2 Results and Discussion

Molecules containing phosphonic acid anchoring groups are capable of forming packed, uniform monolayers on various oxides. Specifically, on ITO substrates, they establish robust bidentate/tri‐dentate bonds with the oxide surface, ensuring stable and consistent monolayer formation. However, when confronted with larger ITO surfaces, various challenges emerge, such as surface defects, thermal stress, and solvent‐induced aggregation of SAMs, which potentially impede the effective anchoring of phosphonic acid onto ITO.^[^ ^7^ ^]^ These drawbacks can compromise the desired properties of the HSL. To tackle these issues, we refrain from employing strong anchoring functional groups such as phosphonic acid, carboxylic acid, and cyanoacetic acid. Instead, we opt for sulfonate anchoring groups with weaker anchoring capabilities to minimize interference with the host SAMs (Figure 1).

Moreover, we noted that numerous SAM molecules exhibit a weak push‐pull effect, resulting in reduced charge extraction/transfer capability of perovskites. Hence, our newly designed guest sulfonate‐based D–π–A SAMs are composed of three key components. The first part, represented in the green region, encompasses the selection of donors, such as DPA, CBZ, and POZ. The second part, depicted in the blue region, includes benzene thiophene, benzothiadiazole, and non‐π–bridge options. By altering the π–bridge, intramolecular push‐pull effects can be achieved, thereby reducing exciton delocalization.^[^ ^8^ ^]^ Finally, the third part, indicated in the yellow region, involves the selection of pyridinium and sulfonates to ensure charge balance while enhancing the push‐pull effect.^[^ ^9^ ^]^ Through this strategic molecular design, our aim is to mitigate the challenges associated with SAM molecules anchoring on ITO substrate and enhance the intramolecular push‐pull effects of SAMs to synergistically optimize the performance of PSCs.

Density Functional Theory (DFT)^[^ ^10^ ^]^ calculations and Time‐Dependent Density‐Functional Theory (TD‐DFT)^[^ ^11^ ^]^ were conducted to gain deeper insights into the electronic structures of sulfonate‐based D–π–A molecules and investigate the competition between the sulfonate‐based D–π–A SAMs and the CNph SAM on the ITO surface. The Gaussian 16 package^[^ ^12^ ^]^ was utilized at the ωB97XD/6‐31+G** level for single molecular computation.^[^ ^13^ ^]^ The frontier molecular orbitals and charge transfer length of the SAMs are depicted in Figure S1 (Supporting Information), and the dihedral angles between donor and acceptor are listed in Table S1 (Supporting Information). As the computational results, the highest occupied molecular orbital (HOMO) and the lowest unoccupied molecular orbital (LUMO) of the SAMs served as host molecules in our study are well separated (so‐called charge‐transfer character) due to molecular design, such as suitable donor moiety, the addition of benzene‐bridge and twisted molecular structure. The charge‐transfer character could effectively reduce the delocalization of the carrier, thereby enhancing the PSC performance.

**Figure 2:**
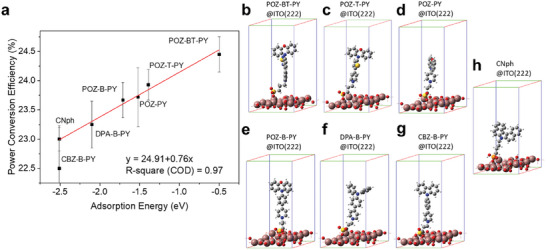
a) Plot of adsorption energies versus the PCE percentage of SAMs on the ITO(222) surface. b–h)The adsorption geometries of SAMs on ITO (222) surface.

For the periodic systems, all the calculations are performed by the Vienna ab initio simulation package (VASP 6.3.1) and employed projector‐augmented waves (PAW) plane‐wave basis set with the Perdew–Burke–Ernzerhof (PBE) functional.^[^ ^14^ ^]^ We use In_16_O_24_ (222) as a surface model ITO(222)^[^ ^15^ ^]^ to simulate the ITO surface and add the adsorbates on the surface to form SAMs@ITO(222) models, which are manifested in **Figure** **2**, and the distance between oxygen atom of sulfonate/ phosphonic acid and the surface are shown in Figure S2 (Supporting Information). For all the geometry optimizations and total energy calculations, a 2 × 2 × 1 Monkhorst–Pack k‐point mesh for sampling the Brillouin zone was used, and a cutoff energy of 400 eV was applied. During the geometry optimizations, atoms in the cell relax until the energy and residual force converge to 10^−3^ eV and 0.02 eV Å^−1^, respectively. A vacuum thickness of 24 Å was adopted along the z‐axis to avoid the interaction between adjacent unit cells. Based on the experiment concept, SAMs' adsorption characteristics are then analyzed. The adsorption energy (*E*_ads_) between surfaces and absorbates listed in Table S2 (Supporting Information) can be calculated by the equation

(1) $$\;E_{ads}=E_{sys}\;-\left(E_{sub}-E_{ab}\right)$$

where *E*_sub_, *E*_ab_, and *E*_sys_ are the energies of the substrates, adsorbates, and substrates with adsorbate, respectively.^[^ ^16^ ^]^

The plot shown in Figure 2 reveals that as the adsorption energy value increases, so does the PCE percentage, consistent with the experimental results shown in Figure S2 (Supporting Information). The results indicate that the adsorption of the guest SAMs can enhance efficiency by filling up the vacancy of the ITO/CNph SAM and should not compete with the adsorption of the host SAMs.

Figure S3 (Supporting Information) compares the cyclic voltammograms (CV) and differential polarized voltammograms (DPV) of the PY‐series molecules in tetrahydrofuran (THF)/tetra‐n‐butylammonium perchlorate (TBAP). The HOMO energy levels for PY‐series molecules are in the order of CBZ‐B‐PY (−5.75 eV) < POZ‐PY (−5.39 eV) < DPA‐B‐PY (−5.32 eV) < POZ‐T‐PY (−5.26 eV) < POZ‐BT‐PY (−5.21 eV) < POZ‐B‐PY (−5.13 eV). The LUMO energy levels were derived from the HOMO energy level and the absorption edge (see Figure S4, Supporting Information). Figure S5 (Supporting Information) illustrates the corresponding energy level diagram. Kelvin probe force microscopy (KPFM) was utilized to assess the average surface contact potential difference (CPD) between ITO/CNph and ITO/CNph@PY‐series films (see Figure S6, Supporting Information). Initial measurements conducted on a gold surface, characterized by a defined work function (WF) of 5.1 eV, allowed for the calibration of CPD to −7 meV. Subsequent measurements on ITO revealed a CPD of 178 meV and a WF of 4.915 eV, while ITO/CNph exhibited a CPD of −211 meV and a WF of 5.304 eV, consistent with previously reported data.^[^ ^6^ ^]^ Notably, ITO/CNph@POZ‐BT‐PY exhibits a CPD of −31 meV and a WF of 5.124 eV (Table S3, Supporting Information). The KPFM results clearly demonstrate the effectiveness of the SAMs@pseudo‐planar strategy, which is attributable to the efficient charge transfer facilitated by the applied current during KPFM measurement. This process ensures the shortest path for the current flow, aligning the work function of the ITO/CNph@PY series closely with that of ITO.

We then verified the chemical anchoring of SAM@pseudo‐planar monolayers by conducting X‐ray photoelectron spectroscopy (XPS) on the ITO surface. Figure S7 (Supporting Information) illustrates a noticeable red shift in the In‐OH peaks of all ITO/CNph@PY‐series of SAMs in comparison with the ITO/CNph SAM, confirming the anchoring of PY‐series SAMs on the ITO surface. Figure S8 (Supporting Information) demonstrates a similar trend in the P 2p spectra, except for the blue shift of the ITO/CNph@CBZ‐B‐PY SAM, indicating that the anchoring capability of CBZ‐B‐PY is inferior to that of other guest SAMs. This affects the inherent host CNph SAM, to certain extent, resulting in the blueshift of the peak. Note that the intensity of the S 2p peak shown in Figure S9 (Supporting Information) indicates that among all ITO/CNph@PY series SAMs, the S signal of CBZ‐B‐PY is weaker, suggesting its inferior anchoring ability to the ITO substrate. This deficiency may have a negative impact on the device performance of inverted PSCs (vide infra).

**Figure 3:**
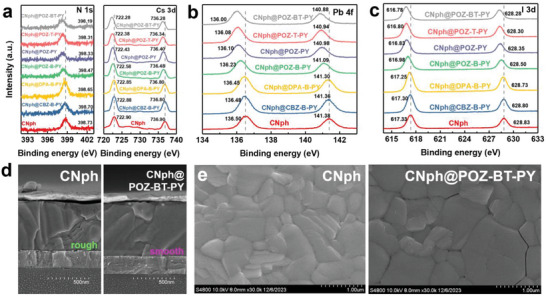
XPS spectra of the core‐level a) N 1s and Cs 3d, b) Pb 4f and c) I 3d elements of perovskite films with varied SAMs. d) Cross‐sectional and e) topographic SEM images of perovskites atop CNph and CNph@POZ‐BT‐Py substrates.

Next, perovskites deposition onto the ITO/CNph@PY‐series SAM was followed by a comprehensive analysis of the XPS spectra for N, Cs, Pb, and I. Shown in **Figure** **3**a, N and Cs peaks are associated with the A site in the perovskite ABX_3_ structure, where as the push‐pull properties of the PY series molecules increase, a notable redshift can be observed with respect to that of the pristine CNph host‐SAM. Particularly, when POZ was selected as the donor unit for the guest‐SAMs, a pronounced red‐shifted spectrum becomes apparent. The results indicate that the lone pair electrons of oxygen on POZ can effectively passivate uncoordinated lead ions on the perovskite surface. In the case of the strongest push‐pull properties observed in the POZ‐BT‐PY SAM, the N 1s and Cs 3d spectrum is red‐shifted by 0.54 and 0.62 eV, respectively, compared to the pristine CNph host‐SAM. Figure 3b depicts the Pb spectrum corresponding to the B site in the perovskite ABX_3_ structure, where a noticeable red shift is observed in the Pb 4f spectra of all ITO/CNph@PY‐series modified SAMs compared to the ITO/CNph SAM. Among them, the CNph@POZ‐BT‐PY modified film displays a maximum redshift of 0.50 eV, which can be attributed to the oxygen atoms on POZ acting as passivation agents for lead ions.^[^ ^17^ ^]^ Figure 3c depicts the I spectrum that is associated with the X site in the perovskite ABX_3_ structure. Again, compared to that of the ITO/CNph SAM, a red‐shifted peak be seen in the I 3d spectra of all ITO/CNph@PY‐series modified SAMs. The maximum red‐shifted peak observed in POZ‐BT‐PY reaches 0.45 eV, resulting from the strongest push‐pull effect that facilitates the coordination of iodine ions with positively charged pyridinium.^[^ ^18^ ^]^

We then examined the morphology of the cross‐sectional and upper surface of the perovskite films, as depicted in Figure 3d,e. The larger average grain size observed on the surface of CNph@POZ‐BT‐PY compared to the pristine counterpart can be attributed to the specific interactions between the functional groups of POZ‐BT‐PY and the perovskite precursor. Relative to the perovskites the oxygen and sulfur atoms in POZ‐BT may virtually act as an electron acceptor, which can effectively passivate the lead defects, while the pyridinium group helps to prevent iodide ion migration. These interactions are conducive to inhibiting the nucleation process and promoting grain growth during the antisolvent step, thereby enhancing the purity of the perovskite intermediate phase. Subsequently, during thermal annealing at 110 °C, the perovskite can transition more smoothly from the bottom to the surface, leading to larger grain sizes.^[^ ^19^ ^]^ Moreover, the cross‐sectional SEM image of the CNph@POZ‐BT‐PY‐based perovskites (Figure 3d) reveals larger and continuous crystal growth with vertically aligned grains, which is conducive to improve the carrier transport. We proceeded with further investigation into the quality changes of perovskite films on various SAM‐modified substrates using X‐ray diffraction (XRD) measurements. The diffraction patterns of perovskite films with different SAM‐modified HSLs are similar, except that a slight PbI_2_ diffraction peak can be observed for the CNph‐treated perovskites (Figure S10, Supporting Information). These results suggest that the SAM@pseudo‐planar monolayer strategy can modulate crystallization by enhancing the grain size and hence reducing the interfacial trap density. The net result is to mitigate charge recombination, thereby improving the *V*_OC_ and FF of devices elaborated in the following device section.

**Figure 4:**
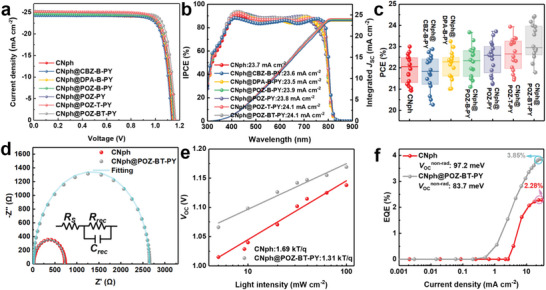
a) Current density−voltage (*J–V*) curves of the champion devices modifying CNph SAM with/without PY‐series treatment measured under 1 sun illumination. b) IPCE spectra of the champion devices with/without PY‐series modification. c) Box plot of PCE values based on different SAM‐modified PSCs. d) Nyquist plot, e) VOC versus light intensity plot, and f) EQE_EL_–*J*_SC_ curves of CNph and CNph@POZ‐BT‐PY‐modified PSCs.

Inverted PSCs were fabricated with a device architecture of MgF_2_/glass/ITO/SAM/perovskite/PCBM/BCP/Ag to evaluate the improvement upon employing the SAM@pseudo‐planar monolayer strategy for enhancing photovoltaic performance. Detailed procedures for device fabrication are provided in the Supporting Information, and the relevant photovoltaic parameters are summarized in **Table** **1**. On the one hand, after optimization, the control device utilizing CNph achieves a maximum PCE of 23.00%, accompanied by a *V*_OC_ of 1.138 V, a *J*_SC_ of 24.62 mA cm^−2^, and an FF of 82.08%. On the other hand, the device modified with CNph@POZ‐BT‐PY, leveraging strong push‐pull effects, demonstrates a highest PCE of 24.45% accompanied by an improved *V*_OC_ of 1.169 V, an enhanced *J*_SC_ of 25.31 mA cm^−2^, and a notable FF of 82.63% (**Figure** **4**a). Among these devices, the PCE of POZ‐B‐PY devices could be improved to 23.67%, which could be attributed to the enhanced *V*_OC_ (1.150 V) and FF (83.51%). Similar trends were observed for devices utilizing DPA‐B‐PY and POZ‐PY as interfacial modifiers. The DPA‐B‐PY device exhibits a PCE of 23.25% with a *V*_OC_ of 1.142 V, a *J*_SC_ of 24.29 mA cm^−2^, and an FF of 83.54%, while the POZ‐PY device achieves a PCE of 23.72% with a *V*_OC_ of 1.155 V, a *J*_SC_ of 24.75 mA cm^−2^, and an FF of 82.97%. Across all PY‐type SAMs, the PCEs of these interfacial modifiers significantly improve the device performance compared to the control CNph PSC, except for the CBZ‐B‐PY cell, possibly due to its inferior anchoring capability compared to other PY SAMs (vide supra). Additionally, incorporating the thiophene π–bridge into CNph@POZ‐T‐PY reduces the delocalization of electrons, leading to a higher *V*_OC_ of 1.162 V, *J*_SC_ of 25.21 mA cm^−2^, FF of 81.69%, and an increased PCE of 23.93%. Ultra‐violet photoelectron spectroscopy (UPS) measurements were employed to further investigate the valence band of perovskites deposited on both CNph‐ and CNph@POZ‐BT‐PY‐modified substrates (Figure S11, Supporting Information). The valence band of perovskite atop the CNph‐treated film was determined to be −5.78 eV, while the valence band of the CNph@POZ‐BT‐PY‐based perovskite film was deduced to be −5.68 eV. Consequently, the presence of CNph@POZ‐BT‐PY facilitates the formation of a more favorable cascade interfacial energy level alignment with perovskites, contributing to the enhancement of *V*_OC_ (Figure S12, Supporting Information). The SAM@pseudo‐planar monolayer strategy also contributes to the amelioration of hysteresis phenomena. The *J–V* hysteresis of the CNph@POZ‐BT‐PY‐treated device is notably reduced compared to that of the CNph counterpart (Figure S13, Supporting Information). This reduction is attributable to the decrease in trap density within the perovskites (vide infra) and the better charge extraction/transport at the interfaces between the HSL and the perovskites.^[^ ^20^ ^]^ The IPCE measurements were conducted to ensure the reliability of the data obtained from the devices. Figure 4b illustrates the integrated JSC values derived from the IPCE spectra, yielding 23.7, 23.6, 23.5, 23.9, 23.8, 24.1, and 24.1 mA cm^−2^ for the CNph, CNph@CBZ‐B‐PY, CNph@DPA‐B‐PY, CNph@POZ‐B‐PY, CNph@POZ‐PY, CNph@POZ‐T‐PY, and CNph@POZ‐BT‐PY devices, respectively. These values are consistent with the *J*_SC_ data extracted from the *J–V* measurements. The results indicate the effectiveness of the SAM@pseudo planar monolayer approach in enhancing the performance of SAM HSL‐based devices. The substantial improvement in *V*_OC_ and FF can be ascribed to the improved hole‐extraction/transport capabilities of the HSL, which is facilitated by repairing SAM packing and well‐aligned interfacial energy levels with perovskites (vide supra). A statistical distribution of the solar efficiency in PSCs with various SAMs is depicted in Figure 4c.

**Table 1:** Photovoltaic parameters of champion PSCs.

Compound	*V*_OC_ [V]	*J*_SC_ [mA cm^−2^]	FF [%]	PCE [%]
CNph	1.138	24.62	82.08	23.00
CNph@CBZ‐B‐PY	1.131	24.29	83.32	22.89
CNph@DPA‐B‐PY	1.142	24.37	83.54	23.25
CNph@POZ‐B‐PY	1.150	24.65	83.51	23.67
CNph@POZ‐PY	1.155	24.75	82.97	23.72
CNph@POZ‐T‐PY	1.162	25.21	81.69	23.93
CNph@POZ‐BT‐PY	1.169	25.31	82.63	24.45

Electrochemical impedance spectroscopy (EIS) was employed to analyze the charge transport dynamics. In Figure 4d, Nyquist plots of devices incorporating CNph and CNph@POZ‐BT‐PY‐modified perovskite films were obtained under dark conditions and fitted using an equivalent circuit model comprising series resistance (*R*_s_) and recombination resistance (*R*_rec_). CNph exhibits a *R*_s_ value of 18.56 Ω, whereas CNph@POZ‐BT‐PY shows a reduced *R*_s_ value of 15.78 Ω. Additionally, the *R*_rec_ value for the CNph@POZ‐BT‐PY device (2649.42 Ω) exceeds that of the CNph counterpart (730.46 Ω). The higher *R*_rec_ for the CNph@POZ‐BT‐PY device suggests inhibition of the charge recombination rate through the pseudo planar POZ‐BT‐PY monolayer treatment, consequently boosting *J*_SC_ and FF in PSCs.^[^ ^21^ ^]^ Furthermore, the charge recombination in PSCs was investigated via light intensity‐dependent *V*_OC_ measurements. The slope values were calculated to be 1.69 kT/q for CNph‐ and 1.31 kT/q for CNph@POZ‐BT‐PY‐based PSCs (Figure 4e). The lower slope indicates faster hole extraction and reduced charge carrier recombination, which results from the repairing and more ordered SAM packing.^[^ ^22^ ^]^ In general, *V*_OC_ and FF values are closely related to charge transport at the HSL/perovskite interface. To evaluate the defect density and extent of charge carrier recombination at this interface, space charge‐limited current (SCLC) measurements were conducted on hole‐only devices with an architecture of ITO/SAMs/perovskite/PM6/MoO_x_/Ag under dark conditions. Beyond the kink point in the bias voltage, the current demonstrates a nonlinear increase, indicating complete filling of trap states. Thus, the trap density of CNph‐ and CNph@POZ‐BT‐PY‐modified devices was determined to be 1.07 × 10^16^ and 5.29 × 10^15^ cm^−3^, respectively, as depicted in Figure S14 (Supporting Information). Furthermore, it is evident that the incorporation of all PY‐series interfacial modifiers effectively mitigates traps of perovskites. This observation aligns with trends observed in light intensity‐dependent *V*_OC_ measurements. Additionally, we investigated carrier migration behavior through electroluminescence (EL) experiments. Figure 4f illustrates the EQE_EL_ of CNph and CNph@POZ‐BT‐PY‐modified PSCs as a function of injection current density. When the injected current density is equal to the *J*_SC_ of the device, EQE_EL_ of PSCs modified by CNph@POZ‐BT‐PY demonstrates an EQE of 3.85% with non‐radiative recombination (Δ*V*_OC_^nrad^) of 83.7 meV, whereas CNph exhibits an EQE of 2.28% with Δ*V*_OC_^nrad^ of 97.2 meV. Higher EQE_EL_ values indicate reduced non‐radiative recombination in PSCs, leading to higher *V*_OC_ in devices.^[^ ^23^ ^]^ These results underscore the capability of SAM@pseudo‐planar monolayer architecture to effectively suppress non‐radiative recombination within PSCs.

**Figure 5:**
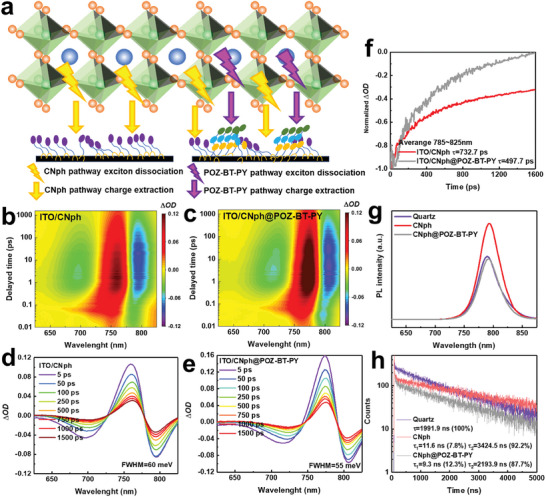
a) Schematic mechanism for the SAM@pseudo‐planar monolayer structure. 2D pseudo‐color plots of the picosecond transient absorption (ps‐TA) spectra for perovskites atop b) CNph‐, and c) CNph@POZ‐BT‐PY‐coated ITO. ps‐TA spectra of perovskites atop d) CNph‐, and e) CNph@POZ‐BT‐PY‐coated ITO under front face excitation. f) The lifetime of ITO/SAM/perovskite films under photo‐induced absorption. g) Steady‐state PL spectra and h) TRPL decay of perovskite films with/without POZ‐BT‐PY passivation.

Typically, perovskites necessitate ≈39 to 45 meV of energy to overcome the exciton binding energy.^[^ ^24^ ^]^ Therefore, a smaller driving force required by the perovskite implies fewer defects in the perovskite film, resulting in faster exciton dissociation decay. Meanwhile, the free electrons and holes liberated from the exciton binding energy might experience weaker push‐pull electronic capabilities of SAMs, necessitating PSC to generate a larger built‐in electric field to drift the free carriers toward the corresponding electrodes. **Figure** **5**a depicts the proposed schematic mechanism for the SAM@pseudo‐planar monolayer architecture. In the left panel, CNph SAM is adsorbed on the ITO substrate, limiting the photovoltaic conversion process to proceed solely through the charge extraction pathway via CNph. Conversely, in the right panel of Figure 5a, POZ‐BT‐PY acts as the guest SAM adsorbed on the ITO substrate, establishing a new pathway to synergistically enhance charge extraction, thereby accelerating charge extraction decay. Picosecond‐transient absorption (ps‐TA) spectra were performed to further understand the exciton dynamics of perovskites. Figure 5b,c depicts typical 2D pseudo‐color plots of the ps‐TA spectra as a function of pump‐probe delay time and probe wavelength, obtained from the deposition of perovskites atop CNph and CNph@POZ‐BT‐PY‐modified films on an ITO substrate. The TA spectra depicted in Figure 5d,e indicate that both the reference CNph and CNph@POZ‐BT‐PY‐modified perovskite films exhibit a positive signal at 770 nm corresponding to photoinduced absorption (PIA) and a negative signal at 800 nm relevant to photobleaching (PB). The PIA signal can be attributed to the depletion of valence band charges, indicating that electrons are removed from the valence band. On the other hand, the PB signal represents the transition of perovskite valence band charges to the conduction band, where the bleaching effect occurs due to the state‐filling in the conduction band. Additionally, the CNph@POZ‐BT‐PY‐modified perovskite film displays a smaller PB peak with a full width at half maximum (FWHM) of 55 meV, whereas the PB peak of the control CNph‐based film has a FWHM of 60 meV. This discrepancy indicates that POZ‐BT‐PY SAM, with its stronger push‐pull electron capability, possesses better passivation ability, which can reduce perovskite defects and suppress exciton delocalization. The reduction in FWHM indicates a decrease in the number of background carriers due to the Coulomb blocking effect, which diminishes bandgap oscillation.^[^ ^25^ ^]^ The FWHM of CNph@DPA‐B‐PY, CNph@POZ‐B‐PY, CNph@POZ‐PY, and CNph@POZ‐T‐PY, as illustrated in Figure S15 (Supporting Information), correlates consistently with the performance of PSCs. Next, we proceeded to fit the exciton dissociation decay of the PB peak, as depicted in Figure 5f. The decay time for CNpha and CNph@POZ‐BT‐PY is fitted to be 732.7 ps and 497.7 ps, respectively. The result suggests that perovskites treated with the SAM@pseudo‐planar monolayer exhibit a smaller exciton binding energy, which facilitates faster exciton dissociation and charge transport.

Steady‐state photoluminescence (PL) and time‐resolved photoluminescence (TRPL) spectra were conducted to directly assess the impact of SAM‐modified ITO on carrier extraction and transport capabilities. As depicted in Figure 5g, the reduced PL intensity of perovskite atop CNph@POZ‐BT‐PY‐modified ITO suggests enhanced charge extraction/transfer efficiency from the perovskite film. The observed blue shift in the PL peak of the CNph@POZ‐BT‐PY modified perovskite film, compared to the CNph modified perovskite film in Figure 4g, can be attributed to the formation of larger and more tightly packed perovskite crystals. These structural changes can influence the optical properties, leading to a blue shift in the PL spectrum.^[^ ^7^, ^26^ ^]^ Figure 5h illustrates that perovskites deposited on quartz display 100% nongeminate recombination decay with a carrier lifetime of 1991.9 ns. The lifetime of perovskites deposited atop ITO/CNph film exhibits a fast charge extraction decay (τ_1_) of 11.6 ns (7.8%) and a slow nongeminate recombination decay (τ_2_) of 3424.5 ns (92.2%), rendering an average lifetime of 3158.3 ns. In contrast, the ITO/CNph@POZ‐BT‐PY/perovskite film reveals a shorter τ_1_ of 9.3 ns (12.3%) and a comparable τ_2_ of 2193.9 ns (87.7%), giving rise to an average lifetime of 1925.2 ns. Following the modification of CNph with the POZ‐BT‐PY monolayer, the shorter lifetime suggests an improvement in charge extraction and transport. This improvement can be attributed to the push‐pull effect of POZ‐BT‐PY along the molecular backbone, which reduces the probability of exciton delocalization and thereby prevents charge recombination as well as charge accumulation at the interface.^[^ ^27^ ^]^ The net result is to substantially enhance the device performance, such as *V*_OC_ and *J*_SC_.^[^ ^28^ ^]^

**Figure 6:**
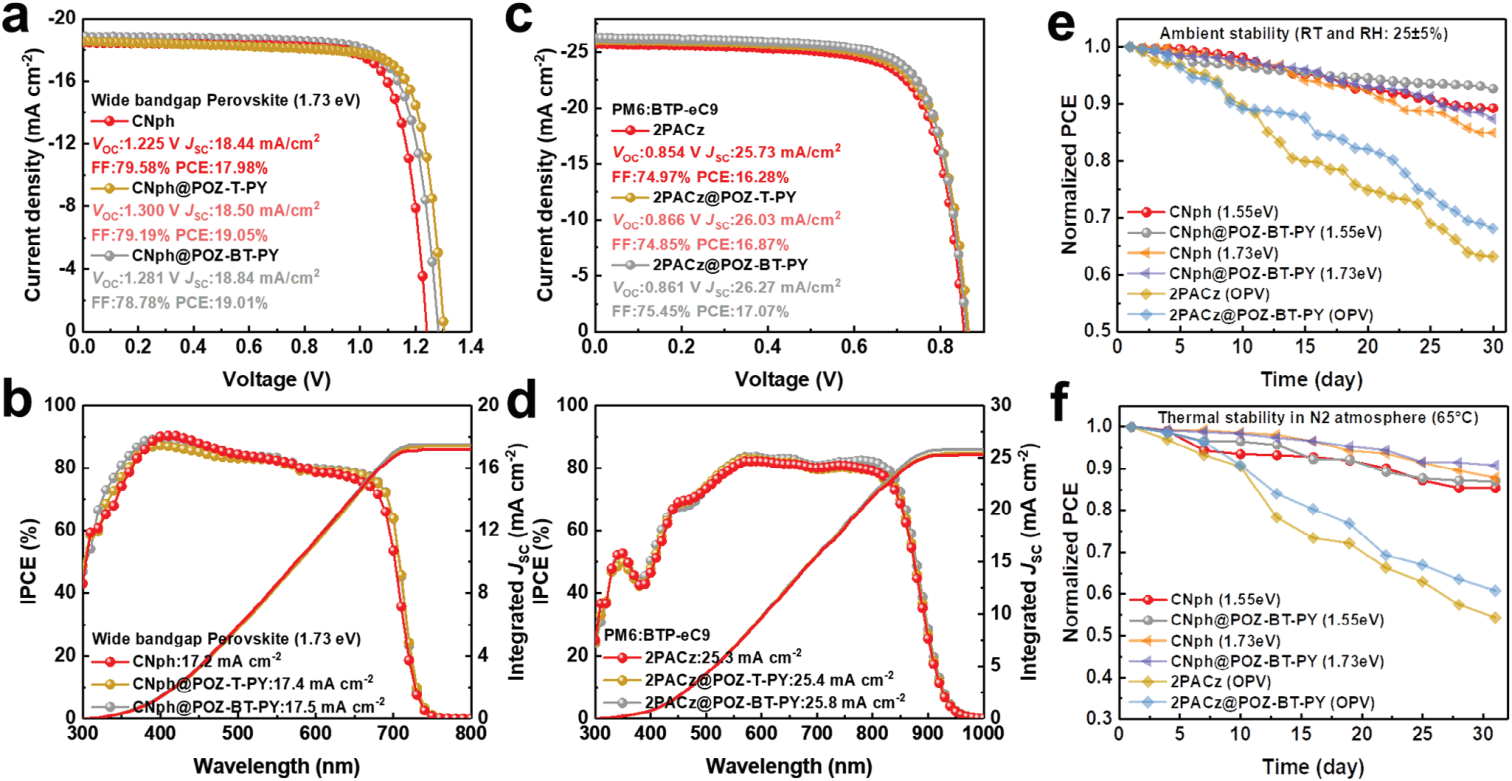
a) *J*–*V* curves of the champion wide bandgap PSCs modified with CNph SAM, with/without PY‐series treatment. b) IPCE spectra of the champion wide bandgap PSCs with/without PY‐series modification. c) *J*–*V* curves of the champion PM6:BTP‐eC9 OPVs modified with CNph SAM, with/without PY‐series treatment. d) IPCE spectra of the champion PM6:BTP‐eC9 OPVs with/without PY‐series modification. e) PCE of the SAM‐modified devices as a function of storage time in ambient air (25 °C and RH: 25±5%). f) Thermal stability test of the SAM‐modified devices at 65 °C in nitrogen‐filled atmosphere.

Apart from implementing the SAM@pseudo‐planar monolayer strategy in inverted PSCs, we have extended this approach to other photovoltaic systems, such as wide‐bandgap PSCs and fullerene‐free organic photovoltaics (OPVs). Wide‐bandgap inverted PSCs, in particular, have received great attention owing to their potential for tandem integration with silicon solar cells. The *J–V* curve of the wide‐bandgap perovskites with a bandgap of 1.73 eV for inverted PSCs, is depicted in **Figure** **6**a. The Control devices based on CNph exhibit a maximum PCE of 17.98% with a *V*_OC_ of 1.225 V, a *J*_SC_ of 18.44 mA cm^−2^, and an FF of 79.58%. In comparison, the CNph@POZ‐T‐PY‐modified devices show significantly enhanced photovoltaic performance with a *V*_OC_ of 1.300 V, a *J*_SC_ of 18.50 mA cm^−2^, and an FF of 79.19%, delivering an impressive PCE of 19.05%. Moreover, devices based on CNph@POZ‐BT‐PY also achieve a notable improvement in PCE to 19.01%, which is attributable to the enhanced *V*_OC_ (1.281 V) and FF (78.78%). Notably, the voltage loss for PY‐modified devices can be reduced to less than 0.45 eV, indicating efficient charge transfer to enhance *V*_OC_. The integrated *J*_SC_ values obtained from the IPCE spectra (Figure 6b) align well with the values extracted from the *J–V* curves. Finally, we utilize fullerene‐free PM6:BTP‐eC9 blends as a model system to further evaluate the feasibility of pseudo‐planar monolayers crossing into OPV systems. The reference device, based on 2PACz, demonstrates a *V*_OC_ of 0.854 V, a *J*_SC_ of 25.73 mA cm^−2^, and an FF of 74.97%, resulting in a PCE of 16.28%. Comparatively, the 2PACz@POZ‐BT‐PY‐modified device performs a maximum PCE of 17.07% along with an improved *J*_SC_ (26.27 mA cm^−2^) and a high FF (75.45%) (Figure 6c). The *J*_SC_ values calculated by integrating the IPCE spectra well match the values acquired from the *J–V* measurements (Figure 6d). These results demonstrate the universal enhancement of SAM HSL‐based device performance through the SAM@pseudo‐planar monolayer strategy.

Last but not least, atmospheric stability tests were initially performed on the photovoltaic devices across various photovoltaic systems under ambient conditions with a relative humidity of 25±5%. Following 30 days of tracking for PSC systems, the PCEs of CNph@POZ‐BT‐PY‐modified devices remain 93% and 87% of their initial values for the perovskites with bandgaps of 1.55 eV and 1.73 eV, respectively, which outperform those of the reference CNph counterparts (Figure 6e). In the case of the OPV system, the PCE of the 2PACz device experiences a significant drop after 30 days, maintaining only 63% of its initial value. Conversely, the PCE of the 2PACz@POZ‐BT‐PY‐modified device retains 68% of its initial value, surpassing that of the reference CNph counterpart. Additionally, we conducted a thermal stability test at 65 °C in a nitrogen‐filled glove box (Figure 6f). The incorporation of POZ‐BT‐PY interfacial modifiers leads to enhanced stability of CNph@POZ‐BT‐PY‐modified PSCs under continuous thermal stress. In particular, after 30 days of monitoring for PSC systems, the PCEs of CNph@POZ‐BT‐PY‐modified devices maintain 87% and 91% of their original efficiencies for perovskites with bandgaps of 1.55 eV and 1.73 eV, respectively. In comparison, the PCEs of reference CNph devices retain 85% and 88% of their initial values for perovskites with bandgaps of 1.55 eV and 1.73 eV, respectively, under the same operational conditions. The grain boundaries in perovskite films are sensitive to thermal stress, which serves as high‐speed pathways for quick diffusion of atomic, ionic, and molecular species. Therefore, perovskite degradation primarily occurs along the weakest grain boundaries of the films.^[^ ^29^ ^]^ Consequently, CNph@POZ‐BT‐PY‐modified devices exhibit superior thermal stability compared to the reference CNph device, which could be attributed to the high‐quality perovskites and effective defect passivation. Furthermore, in the OPV system, the control CNph‐based device degrades more rapidly under the same operational conditions, experiencing a 46% loss in its initial PCE. In stark contrast, the 2PACz@POZ‐BT‐PY‐modified device demonstrates higher thermal durability than the reference CNph device, especially for the 2PACz@POZ‐BT‐PY‐modified device, retaining 61% of the initial PCE even after 30 days. Thus, the application of the SAM@pseudo‐planar monolayer strategy can effectively enhance both the efficiency and stability of PSCs and OPVs.

## 3 Conclusion

In summary, we have synthesized a series of new SAM molecules, including DPA‐B‐PY, CBZ‐B‐PY, POZ‐B‐PY, POZ‐PY, POZ‐T‐PY, and POZ‐BT‐PY, designed to act as interfacial repairers. These SAM molecules were coated atop the CNph SAM layer via a two‐step approach to construct a robust CNph SAM@pseudo‐planar monolayer, and served as the HSL in highly efficient inverted PSCs. The CNph SAM@pseudo‐planar monolayer strategy, featuring a well‐aligned interface with perovskites, played a pivotal role in enhancing charge transfer and effectively passivating deep interfacial defects. This approach notably improved monolayer coverage, stimulated the growth of perovskite crystals, reduced interfacial nonradiative recombination losses, and facilitated hole extraction and transfer. As a result of these enhancements, the POZ‐BT‐PY‐modified device achieved a significantly enhanced PCE of 24.45%, accompanied by an FF of 82.63%. We also demonstrated the application of the CNph SAM@pseudo‐planar monolayer in a wide bandgap PSC, achieving an efficiency exceeding 19%. Additionally, we applied this strategy to a non‐fullerene organic photovoltaic based on the PM6:BTP‐eC9 blend, yielding an impressive efficiency of 17.07%. These modified PSCs and OPVs all demonstrated improved long‐term stability in comparison to their control counterparts. The results underscore the effectiveness of the CNph SAM@pseudo‐planar monolayer strategy in enhancing the performance and stability of various photovoltaic devices, promising advancements in the field of renewable energy technologies.

## Conflict of Interest

The authors declare no conflict of interest.

## Author Contributions

C.‐M.H., C.‐C.W., and Y.‐H.Y. contributed equally to this work. C.‐M.H. conceived, designed, led, and managed the entire experiment, and drafted the initial manuscript. C.‐C.W. performed the theoretical calculations. Y.‐H.Y. synthesized the PY‐series. B.‐H.C., C.‐H.L., and S.‐D.Y. set up and analyzed the transient absorption. C.‐C.C. conducted the OPV experiments. C.‐H.C. carried out experiments on wide bandgap PSCs. C.‐Y.Y. performed experiments on normal bandgap PSCs. Y.‐D.L. conducted steady‐state PL and TRPL measurements. C.‐H.C. performed IPCE measurements. J.‐Y.C. conducted KPFM measurements. I‐C.N. and C.‐I.W. performed XPS and UPS measurements. H.‐C.C. assisted with the drafting of the initial manuscript and completed the final manuscript. P.‐T.C. coordinated and managed the entire experiment and finalized the manuscript. All authors read and approved the final manuscript.

## Supporting information

Supporting Information

## Data Availability

The data that support the findings of this study are available in the supplementary material of this article.
