# Supplementary material for: Repairing Interfacial Defects in Self‐Assembled Monolayers for High‐Efficiency Perovskite Solar Cells and Organic Photovoltaics through the SAM@Pseudo‐Planar Monolayer Strategy
Source: Adv Sci (Weinh). 2024 Jul 30;11(36):2404725. doi: 10.1002/advs.202404725 (PMC11423173; doi:10.1002/advs.202404725)
Supplement: Supplementary file 1 — Supporting Information [file ADVS-11-2404725-s001.docx]

Supporting Information
©Wiley-VCH 2021
69451 Weinheim, Germany

Repairing Interfacial Defects in Self-Assembled Monolayers for High-Efficiency Perovskite Solar Cells and Organic Photovoltaics through the SAM@Pseudo-Planar Monolayer Strategy

Chieh-Ming Hung,^1,+^ Chi-Chi Wu,^1,+^ Yu-Hsuan Yang,^1,+^ Bo-Han Chen,^2^ Chih-Hsuan Lu,^2^ Che-Chun Chu,^1^ Chun-Hao Cheng,^1^ Chun-Yun Yang,^3^ Yan-Ding Lin,^1^ Ching-Hsuan Cheng,^1^ Jiann-Yeu Chen,^4^ I-Chih Ni,^5^ Chih-I Wu,^5^ Shang-Da Yang,^2^ Hsieh-Chih Chen,*^,6^ Pi-Tai Chou*^,1^

* E-mail: [158266@mail.fju.edu.tw](mailto:158266@mail.fju.edu.tw); chop@ntu.edu.tw

Table of Contents

| 1. | Experiment Section…………………………………………..…………… | S3 |
| --- | --- | --- |
|  | Chemicals…………………………………………………….……………. | S3 |
|  | Perovskite Thin Film Fabrication……………………………..………….. | S3 |
|  | Device Fabrication of Inverted Perovskite Solar Cells………..……...... | S3 |
|  | Characterization……………………………………………………………. | S5 |
|  | Synthetic Route and Procedures…………………………….…………… | S7 |
| 2. | Supporting Figures, and Tables………………………………...……....... | S13 |
| 3. | References………………………………………………………………….. | S37 |

1. Experiment Section

**Chemicals:** Cesium chloride (CsCl, 99.9%), formamidinium iodide (FAI, 99.99%), and methylammonium bromide (MABr, 99.99%) were purchased from Dyesol. Phenethylammonium chloride (PEACl, 99.9%), lead iodide (PbI_2_, 99.99%), and lead bromide (PbBr_2_, 99.99%) were obtained from TCI. (4-(7H-dibenzo[c,g]carbazol-7-yl)butyl)phosphonic acid (CbzNaph, CNph, 99%), (2-(9H-carbazol-9-yl)ethyl)phosphonic acid (2PACz, 99%), poly[(2,6-(4,8-bis(5-(2-ethylhexyl-3-fluoro)thiophen-2-yl)-benzo[1,2-b:4,5-b’]dithiophene))-alt-(5,5-(1’,3’-di-2-thienyl-5’,7’-bis(2-ethylhexyl)benzo[1’,2’-c:4’,5’-c’]dithiophene-4,8-dione)] (PM6), BTP-eC9 (99%), poly[(9,9-bis(3'-((N,N-dimethyl)-N-ethylammonium)-propyl)-2,7-fluorene)-alt-2,7-(9,9-dioctylfluorene)]dibromide (PFN-Br), and phenyl-C_61_-butyric acid methyl ester (PCBM, 99%) were sourced from Lumtec. C_60_ (99%) and bathocuproine (BCP, 99.9%) were purchased from Sigma Aldrich. All liquid solvents, including N,N-dimethylformamide (DMF, anhydrous, 99.9%), dimethyl sulfoxide (DMSO, 99.9%), isopropanol (IPA, anhydrous, 99.9%), chlorobenzene (CB, anhydrous, 99.9%), ethanol (EtOH, anhydrous, 99.5%), and methanol (MeOH, anhydrous, 99.5%) were also obtained from Sigma Aldrich.

**1.55 eV Bandgap Perovskite Thin Film Fabrication:** To fabricate the perovskite thin film, the precursor solution was prepared by dissolving 228.76 mg of FAI, 677.67 mg of PbI_2_, 23.57 mg of CsCl, and 7.84 mg of MABr in 0.85 mL of DMF and 0.15 mL of DMSO. An aliquot of 85 μL of this precursor solution was then spin-coated onto the substrate at 5000 rpm for 30 s. During the spin-coating process, 250 μL of CB was dripped onto the film as an anti-solvent 15 s before the end of the spin-coating procedure. Finally, the substrate was annealed at 110 °C for 20 min to form the perovskite film.

**1.73 eV Bandgap Perovskite Thin Film Fabrication:** To fabricate the perovskite thin film, the precursor solution was prepared by dissolving 228.76 mg of FAI, 386.27 mg of PbI_2_, 231.98 mg of PbBr_2_, 23.57 mg of CsCl, and 7.84 mg of MABr in 0.85 mL of DMF and 0.15 mL of DMSO. Subsequently, 85 μL of the prepared precursor solution was spin-coated onto the substrate at 5000 rpm for 30 s. During the spin-coating process, 250 μL of CB was dripped onto the film as an anti-solvent for 15 s before the end of the last procedure. Finally, the substrate was annealed at 120 °C for 20 min to form the perovskite film.

**Device Fabrication of Inverted Perovskite Solar Cells:** The inverted perovskite solar cells were fabricated with the structure MgF_2_/ITO/SAMs/perovskite/PEACl/PCBM/BCP/Ag. Patterned ITO-coated glass substrates (10 Ω per square) were successively cleaned by ultrasonication in 1% neutral detergent in water, deionized water, acetone, and isopropanol for 20 min each, followed by drying under a stream of dry nitrogen. First, a 300 nm MgF_2_ film was vacuum-deposited on the glass side at high vacuum (< 1 × 10^-6^ torr). Then, DI water was dropped onto the ITO side and spin-coated at 5000 rpm for 30 s. The substrates (ITO side) were then subjected to UV−ozone treatment for 20 min and transferred into an N_2_-filled glove box (< 0.1 ppm O_2_ and H_2_O). SAM solutions were prepared by dissolving CNph (0.5 mg/mL) in ethanol and stirring for 60 min at room temperature before deposition. 100 μL of the CNph solution was dropped onto the substrates and spin-coated at 5000 rpm for 30 s. After annealing at 100 °C for 5 min, the substrates were washed with CNph solution (100 μL) through spin-coating at 7000 rpm for 30 s, followed by annealing at 100 °C for 5 min. Next, the PY-series (0.5 mg/mL) was dissolved in DMF, dropped onto the substrates, spin-coated at 5000 rpm for 30 s, and annealed at 100 °C for 5 min. Perovskite thin films were constructed using the same method described in the Perovskite Thin Film Fabrication section. After perovskite deposition, 1.25 mg/mL PEACl in IPA was spin-coated at 5000 rpm for 30 s and annealed at 110 °C for 5 min. After the film cooled down to room temperature, PCBM (20 mg/mL in CB) was deposited onto the perovskite films using spin-coating at 2000 rpm for 30 s. Finally, a 4 nm BCP layer and a 120 nm silver electrode were sequentially evaporated under high vacuum (< 1 × 10^-6^ torr). The effective area of one cell was 0.1 cm^2^.

**Device Fabrication of Organic Photovoltaics:** The normal organic photovoltaics were fabricated with the device structure of ITO/SAMs/active layer/PFN-Br/Ag. The substrates underwent UV−ozone treatment for 20 min and were then transferred into an N_2_-filled glove box (< 0.1 ppm O_2_ and H_2_O). SAM solutions were prepared by dissolving 2PACz (0.4 mg/mL) in ethanol and stirring for 60 min at room temperature before deposition. 100 μL of the SAM solution was dropped onto the substrates and spin-coated at 5000 rpm for 30 s. After annealing at 100 °C for 5 min, the substrates were washed with 2PACz solution (100 μL) through spin-coating at 7000 rpm for 30 s, followed by annealing at 100 °C for 5 min. Next, the PY-series (0.5 mg/mL) was dissolved in DMF, dropped onto the substrates, spin-coated at 5000 rpm for 30 s, and annealed at 100 °C for 5 min. The donor:acceptor blends (1:1.2, weight ratio) were dissolved in chloroform (total concentration of 17.6 mg/mL for all blends) and stirred overnight at room temperature in an N_2_-filled glove box (< 0.1 ppm O_2_ and H_2_O). The blend solution was then spin-cast at 4000 rpm for 30 s after being stirred on a hotplate at 65 °C for 30 min. The wet film was slowly dried in a covered Petri dish for 180 min in the glove box. Afterwards, CB solvent vapor annealing (SVA) treatment was adopted for 1 min. After SVA, PFN-Br (0.5 mg/mL in methanol) was dropped onto the films and spin-coated at 3000 rpm for 30 s. Finally, a 120 nm silver electrode was evaporated under high vacuum (< 1 × 10^-6^ torr). The effective area of one cell was 0.1 cm².

**Characterization:** The *J*–*V* curves were measured using a Newport (Sol3A Class AAA Solar Simulator) AM 1.5 G light source operating at 100 mW/cm^2^ and independently verified with a 300-W AM1.5 G source also operating at 100 mW/cm^2^. The light intensity was determined using a mono-silicon detector with a KG-5 visible color filter, calibrated by the National Renewable Energy Laboratory (NREL) to minimize spectral mismatch. The monochromatic incident photon-to-electron conversion efficiency (IPCE) spectra were measured using a lock-in amplifier with a current preamplifier under short-circuit conditions, supplied by PV Measurement. Devices were illuminated with monochromatic light from a xenon lamp passing through a monochromator, typically at an intensity of 30 μW. A calibrated mono-silicon diode with a known spectral response was used as a reference. The chemical composition and interactions of the perovskite films were confirmed using X-ray photoelectron spectroscopy (XPS) and ultraviolet photoemission spectroscopy (UPS) measurements (ULVAC-PHI, Japan). Scanning electron microscopy (SEM) images were captured by Hitachi S4800. X-ray diffraction (XRD) was measured with a D8-Advance Bruker AXS diffractometer, covering 2θ angles from 5° to 70° under CuKα-radiation (*λ* = 1.54178 Å). Grazing incidence wide-angle X-ray scattering (GIWAXS) patterns were obtained at beamline BL13A1 of the National Synchrotron Radiation Research Center (NSRRC) in Taiwan. The scattering patterns were collected using a Mar165 CCD with a diameter of 40 mm. The scattering vector, *q* = 4π/*λ*sinθ, and the scattering angles in these patterns were calibrated using silver behenate. Kelvin probe force microscopy (KPFM) images were acquired using a Dimension Icon AFM (Bruker). Absorption spectra were obtained with a Hitachi UH-5700 spectrophotometer. Steady-state photoluminescence (PL) spectra and time-resolved studies were performed using a time-correlated single photon counting (TCSPC) system (FLS 980, Edinburgh). Electrochemical impedance spectroscopy (EIS) resistance analysis was conducted using a Metrohm Autolab in an ambient environment at 0.9 V under dark conditions and analyzed with NOVA 2.0 software. The trap-filled limit (*V*_TFL_) was measured under dark conditions using a Keithley 2400 Source Meter. Electroluminescence spectra were characterized using the LQ-50X system (Enlitech, Taiwan), which includes a PTFE integrating sphere, a Multi-Channel Photon Detector (MCD), and two spectrometers for collecting emission photons and conducting subsequent spectral analyses. The MCD enhances sensitivity, allowing for effective detection in low-light conditions. The system can measure a broad wavelength range from 300 to 1700 nm using Si and InGaAs detectors, calibrated against a NIST-traceable standard lamp. Cyclic voltammetry (CV) and differential pulse voltammetry (DPV) analyses were conducted to verify the intrinsic electronic properties of zwitterion derivatives. An Ag/Ag^+^ (0.01 M AgNO_3_) electrode was used as the reference electrode. The oxidation potentials were measured using a platinum electrode in dimethyl sulfoxide as the working electrode, with 0.1 M [NBu_4_]^+^PF_6_^–^ as the electrolyte and platinum wire as the counter electrode. The potentials were further referenced to the ferrocenium/ferrocene (Fc^+^/Fc) couple ($E_{OX}^{Fc}$ = 0.16 V). The HOMO energy levels (*E*_HOMO_) were calculated using the equation *E*_HOMO_ = –(*E*ₒₓ – *E*_FC_ + 4.8) (eV).

**Setup of Light Source and Transient Absorption Spectroscopy:** The detailed schematic setups of our light source and transient absorption spectroscopy are illustrated in our previous study.^[1]^ The details are summarized as follows. The measurements were conducted using a commercial Yb:KGW laser system (Pharos, Light Conversion) with a central wavelength of 1030 nm, an average power of 2.5 W, a repetition rate of 3.125 kHz, a pulse energy of 800 μJ, and a pulse duration of 190 fs. Two identical pulses were generated using a low-GDD 50/50 beam splitter and then passed through our designed nonlinear compressor, employing a technique known as multiple plate compression (MPC).^[2,3]^ A high-pass filter with a cut-off wavelength of 980 nm was applied for this experiment. Pulse compression was achieved with 8 chirped mirror bounces (Ultrafast Innovation) to remove the material dispersion introduced by the optics before the sample. The pump pulse was modulated by a laser-triggered mechanical chopper at half the laser repetition rate (1.5625 kHz). A broadband half-wave plate and wire-grid polarizer were employed to precisely control the excitation power and prevent any nonlinear effects. The delay time of the probe pulse relative to the pump pulse was adjusted using a linear translation stage (DL325, Newport) with a delay range of up to approximately 2.2 ns. The pump and probe beams were focused on the sample in a noncollinear manner with a cross-angle of 5°. Different focusing conditions were chosen for the pump and probe pulses to ensure that the focused pump spot size (~67.7 μm) was slightly larger than the focused probe spot size (~27.3 μm), ensuring uniform excitation of the probed region. After passing through the sample, the transmitted probe pulse was spatially separated and guided into our designed spectrometer. The spectrometer included a high-speed linear array camera (Glaz Linescan-I-Gen2, Synertronic with S12198-512Q CMOS, Hamamatsu) to capture each probe pulse. Since the pump pulses were modulated at half the repetition rate, the spectral difference between every two probe shots (one exposed to the pump and the other not) provided the Δ*T*/*T* signal.

**Synthetic Route and Procedures :**

**General Methods and Materials**

Unless otherwise noted, commercially available reagents were purchased from Sigma-Aldrich, Acros Organics, Combi-Blocks, AK Scientific, and Alfa Aesar without further purification. Solvent purification followed the procedures outlined in "Purification of Laboratory Chemicals" (Perrin, D. D.; Armarego, W. L. and Perrins, D. R., Pergamon Press: Oxford, 1980). All reactions were carried out under a nitrogen atmosphere and monitored by thin layer chromatography (TLC) using precoated Merck silica gel 60 F254 alumina plates (0.25 mm). Visualization was accomplished using ultraviolet light at wavelengths of 256 nm and 365 nm. Column chromatography was performed using silica gel (230−400 mesh) supplied by Merck. ^1^H and ^13^C NMR spectra were recorded at 298 K on an Agilent Mercury 400 spectrometer, with DMSO-d6 (δ 2.50 for ^1^H NMR and δ 39.52 for ^13^C NMR) as the internal reference. The following abbreviations were used to describe the multiplicities: s = singlet, d = doublet, t = triplet, qui = quintet, and m = multiplet. High-resolution mass spectra (HRMS) were recorded on a Bruker ultrafleXtreme instrument in the Matrix-Assisted Laser Desorption/Ionization (MALDI) mode. Column chromatography was carried out using silica gel (230−400 mesh) supplied by Merck. 1H, 13C spectra were recorded at 298 K on Agilent Mercury 400 spectrometer, is reported with DMSO-d6 (δ 2.50 for ^1^H NMR and δ 39.52 for ^13^C NMR) as internal references. The following abbreviations were used to explain the multiplicities: s = singlet, d = doublet, t = triplet, qui = quintet, m = multiplet. High resolution mass spectra (HRMS) were recorded on Bruker ultrafleXtrene TM instrument in the Matrix- Assisted Laser Desorption/ Ionization (MALDI) mode. **Figures S16-40** provide the mass spectra, as well as the ^13^C and ^1^H NMR spectra for the intermediates and final products.

**Synthesis and Characterization**

**Synthesis of N,N-Diphenyl-4-(pyridin-4-yl)aniline (1)**

4-Bromotriphenylamine (5.00 g, 15.48 mmol), 4-pyridylboronic acid (2.34 g, 19.0 mmol), Pd(PPh_3_)_2_Cl_2_ (548 mg, 0.77 mmol) and K_2_CO_3_ (6.42 g, 46.44 mmol) were added to the mixture solution of toluene/EtOH/H_2_O (30 mL/15 mL/15 mL). The mixture was heated at 110 °C for 16 h under nitrogen atmosphere. After cooling to room temperature, the mixture was extracted with EtOAc (3 × 20 mL), and the combined extracts were washed with brine and dried over anhydrous MgSO_4_. After filtration and concentration under reduced pressure. The crude residue was purified by column chromatography on silica gel (hexane:EtOAc = 5:2) to afford the desired product (3.54 g, 71%) as solid. ^1^H NMR (400 MHz, DMSO-*d_6_*, 298 K) δ (ppm): 8.56 (d, *J* = 5.1 Hz, 2H), 7.71 (d, *J* = 8.3 Hz, 2H), 7.63 (d, *J* = 5.1 Hz, 2H), 7.33 (t, *J* = 7.7 Hz, 4H), 7.08 (t, *J* = 7.0 Hz, 6H), 7.01 (d, *J* = 8.4 Hz, 2H). ^13^C NMR (101 MHz, DMSO-*d_6_*, 298 K) δ (ppm): 150.11, 148.39, 146.64, 146.27, 129.97, 129.69, 127.77, 124.74, 123.80, 122.13, 120.39.

**Synthesis of 9-(4-bromophenyl)-9H-carbazole (2)**

Carbazole (1.00 g, 5.98 mmol), 1-bromo-4-iodobenzene (2.03 g, 7.18 mmol), Pd(OAc)_2_ (13.4 mg, 0.0598 mmol), xantphos (34.6 mg, 0.0598 mmol) and sodium tert-butoxide (862 mg, 8.97 mmol) were added to the anhydride toluene (20 mL). The mixture was heated to reflux for 16 h under nitrogen atmosphere. After cooling to room temperature, the reaction was quenched with 30 mL water and extracted with EtOAc (3 × 20 mL), and the combined extracts were washed with brine and dried over anhydrous MgSO_4_. After filtration and concentration under reduced pressure. The crude residue was purified by column chromatography on silica gel (hexane:EtOAc = 3:1) to afford the desired product (1.31 g, 68%) as white solid. ^1^H NMR (400 MHz, DMSO-*d_6_*, 298 K) δ (ppm): 8.25 (d, *J* = 6.8 Hz, 2H), 7.86 (dd, *J* = 8.7, 1.2 Hz, 2H), 7.60 (dd, *J* = 8.7, 1.2 Hz, 2H), 7.43 (dt, *J* = 6.8, 1.3 Hz, 2H), 7.39 (dd, *J* = 8.2, 1.0 Hz, 2H), 7.30 (tt, *J* = 8.1, 1.3 Hz, 2H).

**Synthesis of 9-(4-(pyridin-4-yl)phenyl)-9H-carbazole (3)**

9-(4-Bromophenyl)-9H-carbazole (1.00 g, 3.12 mmol), 4-pyridylboronic acid (456 mg, 3.74 mmol), Pd(PPh_3_)_2_Cl_2_ (109 mg, 0.156 mmol) and K_2_CO_3_ (1.29 g, 9.35 mmol) were added to the mixture solution of toluene/EtOH/H_2_O (6 mL/3 mL/3 mL). The mixture was heated at 110 °C for 16 h under nitrogen atmosphere. After cooling to room temperature, the mixture was extracted with EA (3 × 10 mL), and the combined extracts were washed with brine and dried over anhydrous MgSO_4_. After filtration and concentration under reduced pressure. The crude residue was purified by column chromatography on silica gel (hexane:EA = 3:1) to afford the 3 (501 g, 50%) as white solid. ^1^H NMR (400 MHz, DMSO-*d_6_*, 298 K) δ (ppm): 8.71 (d, *J* = 5.0 Hz, 2H), 8.27 (dd, *J* = 7.8, 1.0 Hz, 2H), 8.12 (d, *J* = 7.7 Hz, 2H), 7.82 (dd, *J* = 17.4, 6.4 Hz, 4H), 7.46 (d, *J* = 6.2 Hz, 4H), 7.36 – 7.28 (m, 2H).

**Synthesis of 10-(4-bromophenyl)-10H-phenoxazine (4)**

Phenoxazine (1.00 g, 5.46 mmol), 1-bromo-4-iodobenzene (2.03 g, 7.18 mmol), Pd(OAc)_2_ (13.4 mg, 0.0598 mmol), xantphos (34.6 mg, 0.0598 mmol) and sodium tert-butoxide (862 mg, 8.97 mmol) were added to the anhydride toluene (20 mL). The mixture was heated to reflux for 16 h under nitrogen atmosphere. After cooling to room temperature, the reaction was quenched with 30 mL water and extracted with EtOAc (3 × 20 mL), and the combined extracts were washed with brine and dried over anhydrous MgSO_4_. After filtration and concentration under reduced pressure. The crude residue was purified by column chromatography on silica gel (hexane:EtOAc = 100:1) to afford the desired product (1.32 g, 72%) as white solid. ^1^H NMR (400 MHz, DMSO-*d_6_*, 298 K) δ (ppm): 7.85 (dd, *J* = 8.5, 1.4 Hz, 2H), 7.40 (dd, *J* = 8.6, 1.4 Hz, 2H), 6.78 – 6.63 (m, 6H), 5.91 – 5.84 (m, 2H).

**Synthesis of 10-(4-(pyridin-4-yl)phenyl)-10H-phenoxazine (5)**

4 (600 mg, 1.78 mmol), 4-pyridylboronic acid (239 g, 1.96 mmol), Pd(PPh_3_)_2_Cl_2_ (63 mg, 0.089 mmol) and K_2_CO_3_ (738 mg, 5.34 mmol) were added to the mixture solution of toluene/EtOH/ H_2_O (6 mL/3 mL/3 mL). The mixture was heated at 110 °C for 16 h under nitrogen atmosphere. After cooling to room temperature, the mixture was extracted with EA (3 × 10 mL), and the combined extracts were washed with brine and dried over anhydrous MgSO_4_. After filtration and concentration under reduced pressure. The crude residue was purified by column chromatography on silica gel (hexane:EA = 3:1) to afford the 5 (245 g, 41%) as yellow solid. ^1^H NMR (400 MHz, DMSO-*d_6_*, 298 K) δ (ppm): 8.69 (d, *J* = 6.1 Hz, 2H), 8.10 (d, *J* = 8.3 Hz, 2H), 7.81 (d, *J* = 6.3 Hz, 2H), 7.58 (d, *J* = 8.3 Hz, 2H), 6.82 – 6.74 (m, 2H), 6.73 – 6.62 (m, 4H), 5.99 – 5.91 (m, 2H).

**Synthesis of 10-(pyridin-4-yl)-10H-phenoxazine (6)**

Phenoxazine (1.00 g, 5.46 mmol), 4-bromo pyridine (1.27 g, 6.55 mmol), Pd(OAc)_2_ (61 mg, 0.273 mmol), xantphos (260 mg, 0.546 mmol) and K_2_CO_3_ (2.26 g, 8.97 mmol) were added to the anhydride toluene (18 mL). The mixture was heated to reflux for 16 h under nitrogen atmosphere. After cooling to room temperature, the reaction was quenched with 30 mL water and extracted with EtOAc (3 × 20 mL), and the combined extracts were washed with brine and dried over anhydrous MgSO_4_. After filtration and concentration under reduced pressure. The crude residue was purified by column chromatography on silica gel (hexane:EtOAc = 3:1) to afford the desired product (1.30 g, 92%) as white solid. ^1^H NMR (400 MHz, DMSO-*d_6_*, 298 K) δ (ppm): 8.54 (d, *J* = 7.8 Hz, 2H), 7.79 (d, *J* = 7.8 Hz, 3H), 7.59 (d, *J* = 7.8 Hz, 2H), 7.48 – 7.29 (m, 12H), 4.45 (d, *J* = 6.9 Hz, 3H), 2.44 – 2.25 (m, 2H), 2.10 (s, 1H).

**Synthesis of 4-bromo-7-(pyridin-4-yl)benzo[c][1,2,5]thiadiazole (7)**

4,7-Dibromobenzo[c]-1,2,5-thiadiazole (647 mg, 2.20 mmol), 4-pyridylboronic acid (244 mg, 2.00 mmol), Pd(PPh_3_)_4_ (16 mg, 0.100 mmol), and K_2_CO_3_ (832 mg, 6.00 mmol) were added to the 1,4-dioxane/H_2_O (15 mL/5 mL). The mixture was heated to reflux for 16 h under nitrogen atmosphere. After cooling to room temperature, the reaction was quenched with 30 mL water and extracted with EtOAc (3 × 20 mL), and the combined extracts were washed with brine and dried over anhydrous MgSO_4_. After filtration and concentration under reduced pressure. The crude residue was purified by column chromatography on silica gel (hexane:EtOAc = 1:1) to afford the desired product (211 g, 33%) as light yellow solid. ^1^H NMR (400 MHz, DMSO-*d_6_*, 298 K) δ (ppm): 8.77 – 8.72 (m, 2H), 8.19 (d, *J* = 7.6 Hz, 1H), 8.02 – 7.98 (m, 2H), 7.97 (d, *J* = 7.6 Hz, 1H).

**Synthesis of 10-(7-(pyridin-4-yl)benzo[c][1,2,5]thiadiazol-4-yl)-10H-phenoxazine (8)**

7 (500 mg, 1.72 mmol), phenoxazine (472 mg, 2.58 mmol), Pd(OAc)_2_ (59 mg, 0.263 mmol), [(t-Bu)_3_PH]BF_4_ (229 mg, 0.789 mmol) and sodium tert-butoxide (496 mg, 5.16 mmol) were added to the anhydride toluene (20 mL). The mixture was heated to reflux for 16 h under nitrogen atmosphere. After cooling to room temperature, the reaction was quenched with 30 mL water and extracted with EtOAc (3 × 20 mL), and the combined extracts were washed with brine and dried over anhydrous MgSO_4_. After filtration and concentration under reduced pressure. The crude residue was purified by column chromatography on silica gel (hexane:EtOAc = 1:1) to afford the desired product (472 mg, 70%) as purple solid. ^1^H NMR (400 MHz, DMSO-*d_6_*, 298 K) δ (ppm): 8.82 – 8.78 (m, 2H), 8.24 (d, *J* = 7.4 Hz, 1H), 8.11 – 8.09 (m, 2H), 8.07 (d, *J* = 7.5 Hz, 1H), 6.82 (dd, *J* = 7.9, 1.5 Hz, 2H), 6.71 (td, *J* = 7.7, 1.5 Hz, 2H), 6.62 – 6.56 (m, 1H), 5.92 (dd, *J* = 8.0, 1.5 Hz, 2H). ) ^13^C NMR (101 MHz, DMSO-*d_6_*, 298 K) δ (ppm): δ 154.48, 152.11, 150.08, 143.53, 143.17, 133.30, 133.08, 131.45, 130.13, 129.79, 123.79, 123.65, 121.96, 115.48, 113.71. HRMS (MALDI) C_23_H_14_N_4_OS calcd. [M+H]^+^ for 395.097, found 359.096.

**Synthesis of 4-(5-bromothiophen-2-yl)pyridine (9)**

2,5-Dibromothiophene (305 μL, 2.70 mmol), 4-pyridylboronic acid (300 mg, 2.46 mmol), Pd(PPh_3_)_2_Cl_2_ (86 mg, 0.123 mmol), and K_2_CO_3_ (1.02 g, 7.38 mmol) were added to the mixture solution of toluene/EtOH/H_2_O (5 mL/3 mL/2 mL). The mixture was heated to 130 °C for 2.5 h under nitrogen atmosphere. After cooling to room temperature, the reaction was quenched with 30 mL water and extracted with EtOAc (3 × 5 mL), and the combined extracts were washed with brine and dried over anhydrous MgSO_4_. After filtration and concentration under reduced pressure. The crude residue was purified by column chromatography on silica gel (hexane:EtOAc = 1:1) to afford the desired product (76 g, 12%) as light yellow solid. ^1^H NMR (400 MHz, DMSO-*d_6_*, 298 K) δ (ppm): δ 8.60 – 8.55 (m, 2H), 7.68 (d, *J* = 4.0 Hz, 1H), 7.63 – 7.59 (m, 2H), 7.36 (d, *J* = 4.0 Hz, 1H).

**Synthesis of 10-(5-(pyridin-4-yl)thiophen-2-yl)-10H-phenoxazine (10)**

9 (70 g, 0.293 mmol), phenoxazine (59 mg, 0.322 mmol), Pd_2_(dba)_3_ (11 mg, 0.012 mmol), [(t-Bu)_3_PH]BF_4_ (6.8 mg, 0.023 mmol) and sodium tert-butoxide (56 mg, 0.586 mmol) were added to the anhydride xylene (10 mL). The mixture was heated to reflux for 16 h under nitrogen atmosphere. After cooling to room temperature, the reaction was quenched with 30 mL water and extracted with EtOAc (3 × 10 mL), and the combined extracts were washed with brine and dried over anhydrous MgSO_4_. After filtration and concentration under reduced pressure. The crude residue was purified by column chromatography on silica gel (hexane:EtOAc = 1:1) to afford the desired product (61 mg, 61%) as light yellow solid. ^1^H NMR (400 MHz, DMSO-*d_6_*, 298 K) δ (ppm): 8.61 (d, *J* = 6.2 Hz, 2H), 7.94 (d, *J* = 3.9 Hz, 1H), 7.79 – 7.66 (m, 2H), 7.35 (d, *J* = 3.9 Hz, 1H), 6.94 – 6.72 (m, 6H), 6.34 (dd, *J* = 5.9, 3.1 Hz, 2H). ^13^C NMR (101 MHz, DMSO-*d_6_*, 298 K) δ (ppm): 150.48, 143.16, 141.05, 140.62, 140.10, 132.97, 131.27, 126.24, 124.02, 122.84, 119.49, 115.63, 114.21. HRMS (MALDI) C_21_H_14_N_2_OS calcd [M+H]^+^ for 343.091, found 343.089.

**General procedure for preparation of Zwitterions**

The starting materials, pyridine derivatives (1 equiv.), was dissolved in dried toluene (0.04 M). After the mixture was stirred for 20 min at room temperature, 1,3-propanesultone (1 equiv.) was added. Then, the reaction mixture was heated at 120 °C for 48 h under nitrogen atmosphere. After cooling to room temperature, the resultant precipitate was washed with toluene. The crude residue was collected by subsequently washed with toluene (6 mL x 1) and diethyl ether (6 mL x 3) and dried under vacuum to afford the zwitterion products.

**3-(4-(4-(Diphenylamino)phenyl)pyridin-1-ium-1-yl)propane-1-sulfonate (DPA-B-PY)**

Following general procedure, 1 (300 mg, 0.931 mmol) to afford DPA-B-PY (182 mg, 44%) as yellow solid. ^1^H NMR (400 MHz, DMSO-*d_6_*, 298 K) δ (ppm): 8.93 (d, *J* = 7.1 Hz, 2H), 8.34 (d, *J* = 7.1 Hz, 2H), 7.98 (d, *J* = 9.0 Hz, 2H), 7.40 (dd, *J* = 8.4, 7.4 Hz, 4H), 7.27 – 7.06 (m, 6H), 6.94 (d, *J* = 8.9 Hz, 2H), 4.62 (t, *J* = 6.9 Hz, 3H), 2.41 (t, *J* = 7.1 Hz, 2H), 2.21 (q, *J* = 6.9 Hz, 2H). ^13^C NMR (101 MHz, DMSO-*d_6_*, 298 K) δ (ppm): 153.61, 151.12, 145.68, 144.41, 130.03, 129.57, 126.10, 125.27, 124.51, 122.53, 119.58, 58.23, 46.99, 27.26. HRMS (MALDI) C_26_H_24_N_2_O_3_S calcd. [M+H]^+^ for 445.1580, found 445.1586.

**3-(4-(4-(9H-carbazol-9-yl)phenyl)pyridin-1-ium-1-yl)propane-1-sulfonate (CBZ-B-PY)**

Following general procedure, 3 (250 mg, 0.780 mmol) to afford CBZ-B-PY (344.8 mg, quant.) as yellow solid. ^1^H NMR (400 MHz, DMSO-*d_6_*, 298 K) δ (ppm): 9.16 (d, *J* = 5.5 Hz, 2H), 8.62 (d, *J* = 5.5 Hz, 2H), 8.38 (dd, *J* = 8.5, 1.5 Hz, 2H), 8.28 (dd, *J* = 7.7, 1.1 Hz, 2H), 7.93 (dd, *J* = 8.5, 1.6 Hz, 2H), 7.55 – 7.43 (m, 4H), 7.34 (td, *J* = 7.4, 6.8, 1.3 Hz, 2H), 4.77 (t, *J* = 6.9 Hz, 2H), 2.30 (p, *J* = 7.0 Hz, 2H). ^13^C NMR (101 MHz, DMSO-*d_6_*, 298 K) δ (ppm): 153.66, 145.06, 140.25, 139.60, 132.21, 130.11, 127.24, 126.50, 124.52, 123.17, 120.66, 109.80, 58.80, 46.99, 27.31. HRMS (MALDI) C_26_H_22_N_2_O_3_S calcd. [M+H]^+^ for 443.143, found 443.142.

**3-(4-(10H-phenoxazin-10-yl)pyridin-1-ium-1-yl)propane-1-sulfonate (POZ-B-PY)**

Following general procedure, 5 (245.4 mg, 0.730 mmol) to afford POZ-B-PY (295 mg, 88.2%) as orang solid. ^1^H NMR (400 MHz, DMSO-*d_6_*, 298 K) δ (ppm): 9.17 (d, *J* = 6.4 Hz, 1H), 8.60 (d, *J* = 6.5 Hz, 1H), 8.36 (d, *J* = 8.5 Hz, 1H), 7.72 (d, *J* = 8.5 Hz, 1H), 6.83 – 6.77 (m, 1H), 6.76 – 6.64 (m, 2H), 5.97 (dd, *J* = 7.3, 2.1 Hz, 1H), 4.75 (t, *J* = 6.7 Hz, 1H), 3.36 (t, *J* = 6.5 Hz, 0H), 2.36 – 2.21 (m, 1H). ^13^C NMR (101 MHz, DMSO-*d_6_*, 298 K) δ (ppm): 153.68, 145.11, 143.26, 141.88, 133.81, 133.37, 131.65, 131.23, 124.82, 123.77, 121.92, 115.53, 113.40, 58.87, 46.96, 27.30. HRMS (MALDI) C_26_H_22_N_2_O_4_S calcd. [M+H]^+^ for 459.138, found 459.137.

**3-(4-(10H-phenoxazin-10-yl)pyridin-1-ium-1-yl)propane-1-sulfonate (POZ-PY)**

Following general procedure, 6 (700 mg, 2.691 mmol) to afford POZ-PY (1.03 g, quant.) as white solid. ^1^H NMR (400 MHz, DMSO-*d_6_*, 298 K) δ (ppm): 8.56 (d, *J* = 7.8 Hz, 2H), 7.81 (d, *J* = 7.8 Hz, 2H), 7.61 (d, *J* = 7.8 Hz, 2H), 7.50 – 7.31 (m, 6H), 4.47 (t, *J* = 6.8 Hz, 2H), 2.46 – 2.27 (m, 2H), 2.10 (t, *J* = 7.0 Hz, 2H). ^13^C NMR (101 MHz, DMSO-*d_6_*, 298 K) δ (ppm): 154.68, 152.08, 144.43, 128.76, 128.73, 125.32, 124.70, 118.11, 110.00, 56.87, 46.95, 27.13. HRMS (MALDI) C_20_H_18_N_2_O_4_S calcd. [M+H]^+^ for 383.107, found 383.108.

**3-(4-(7-(10H-phenoxazin-10-yl)benzo[c][1,2,5]thiadiazol-4-yl)pyridin-1-ium-1-yl)propane-1-sulfonate (POZ-BT-PY)**

Following general procedure, 8 (362 mg, 0.919 mmol) to afford POZ-BT-PY (452 mg, 98%) as purple solid. ^1^H NMR (400 MHz, DMSO-*d_6_*, 298 K) δ (ppm): 9.29 (d, *J* = 7.0 Hz, 2H), 8.88 (d, *J* = 7.0 Hz, 2H), 8.57 (d, *J* = 7.5 Hz, 1H), 8.20 (d, *J* = 7.5 Hz, 1H), 6.83 (dd, *J* = 7.9, 1.5 Hz, 2H), 6.73 (td, *J* = 7.7, 1.5 Hz, 2H), 6.60 (td, *J* = 7.7, 1.5 Hz, 2H), 5.96 (dd, *J* = 8.0, 1.4 Hz, 2H), 4.84 (d, *J* = 6.9 Hz, 2H), 2.39 – 2.24 (m, 2H). ^13^C NMR (101 MHz, DMSO-*d_6_*, 298 K) δ (ppm): 154.15, 152.15, 151.24, 145.06, 143.17, 133.20, 132.87, 132.60, 132.50, 127.73, 127.05, 123.78, 122.20, 115.60, 113.82, 59.27, 47.04, 27.32. HRMS(MALDI) C_26_H_20_N_4_O_4_S_2_ calcd. [M+H]^+^ for 517.101, found 517.098.

**3-(4-(5-(10H-phenoxazin-10-yl)thiophen-2-yl)pyridin-1-ium-1-yl)propane-1-sulfonate (POZ-T-PY)**

Following general procedure, 10 (185 mg, 0.541 mmol) to afford POZ-T-PY (235.4 mg, 93.8%) as orang solid. ^1^H NMR (400 MHz, DMSO-*d_6_*, 298 K) δ (ppm): 9.02 (d, *J* = 6.8 Hz, 2H), 8.50 – 8.29 (m, 3H), 7.52 (d, *J* = 3.9 Hz, 1H), 6.95 – 6.75 (m, 6H), 6.45 – 6.34 (m, 2H), 4.67 (t, *J* = 6.9 Hz, 2H), 2.46 (t, *J* = 7.0 Hz, 2H), 2.24 (t, *J* = 7.0 Hz, 2H). ^13^C NMR (101 MHz, DMSO-*d_6_*, 298 K) δ (ppm): 147.43, 146.66, 145.02, 143.44, 136.51, 132.62, 132.18, 131.70, 124.09, 123.35, 122.44, 115.87, 114.69, 58.67, 46.95, 27.22. HRMS (MALDI) C_24_H_20_N_2_O_4_S_2_ calcd. [M+H]^+^ for 465.094, found 465.092

| **Compounds** | **Charge Transfer Length (Å)** | **HOMO** | **LUMO** |
| --- | --- | --- | --- |
| DPA-B-PY | 5.18 | 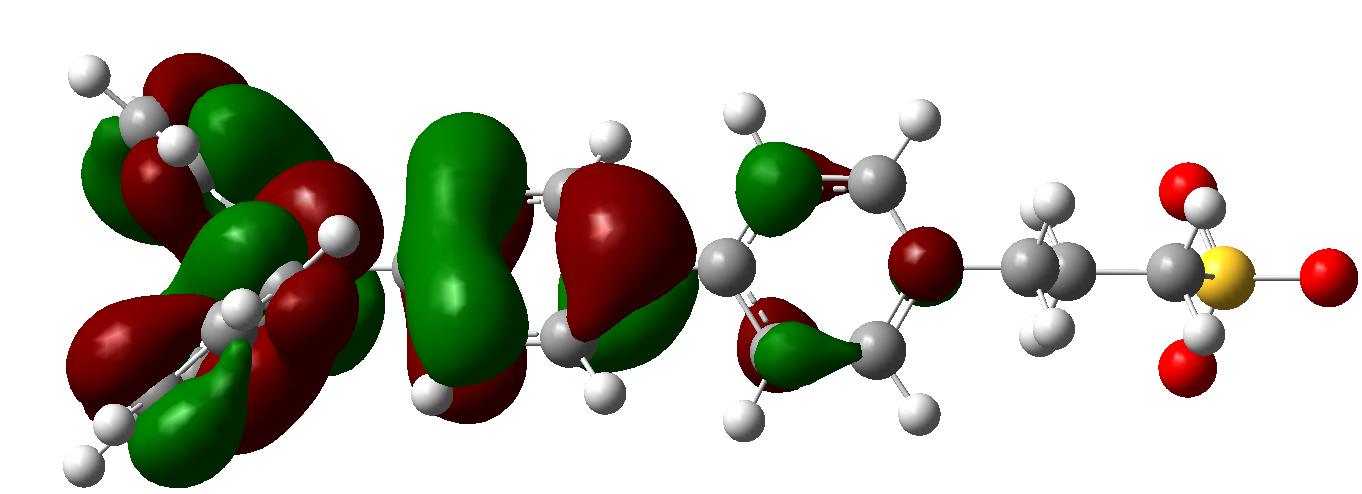 | 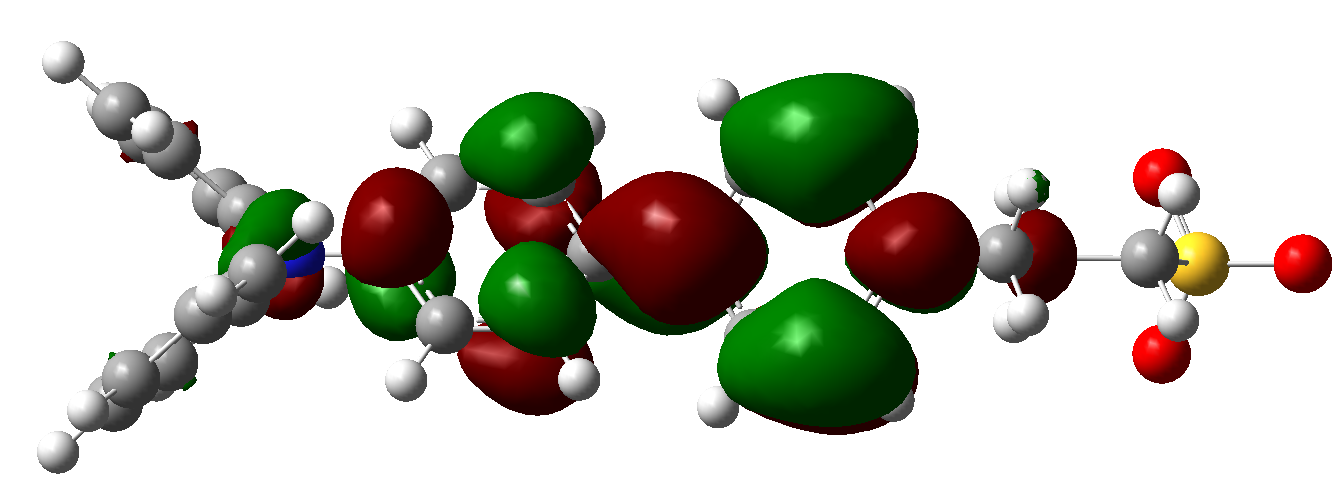 |
| CBZ-B-PY | 6.17 | 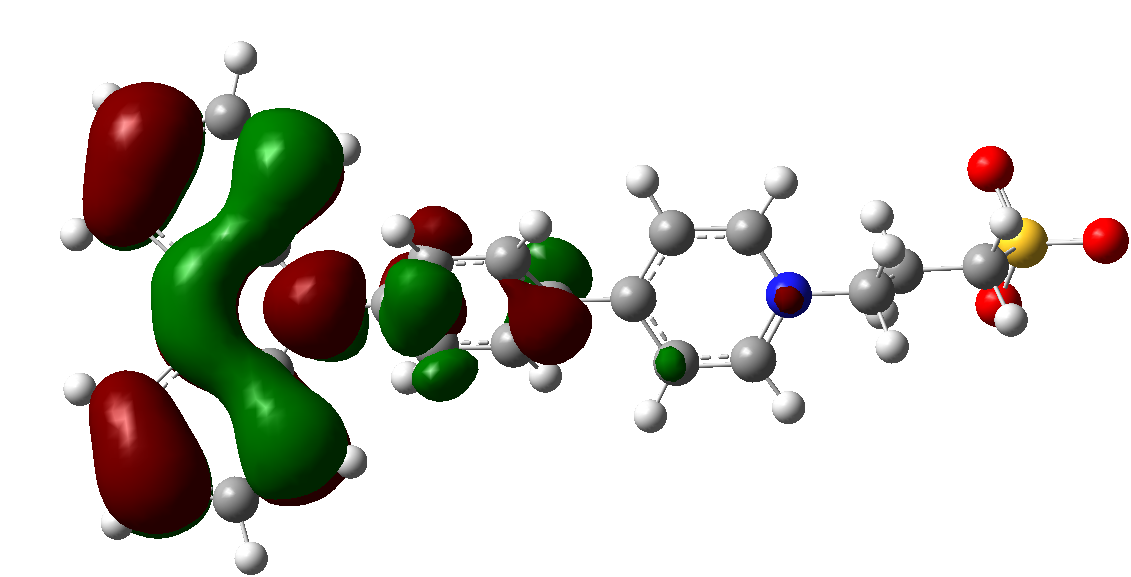 | 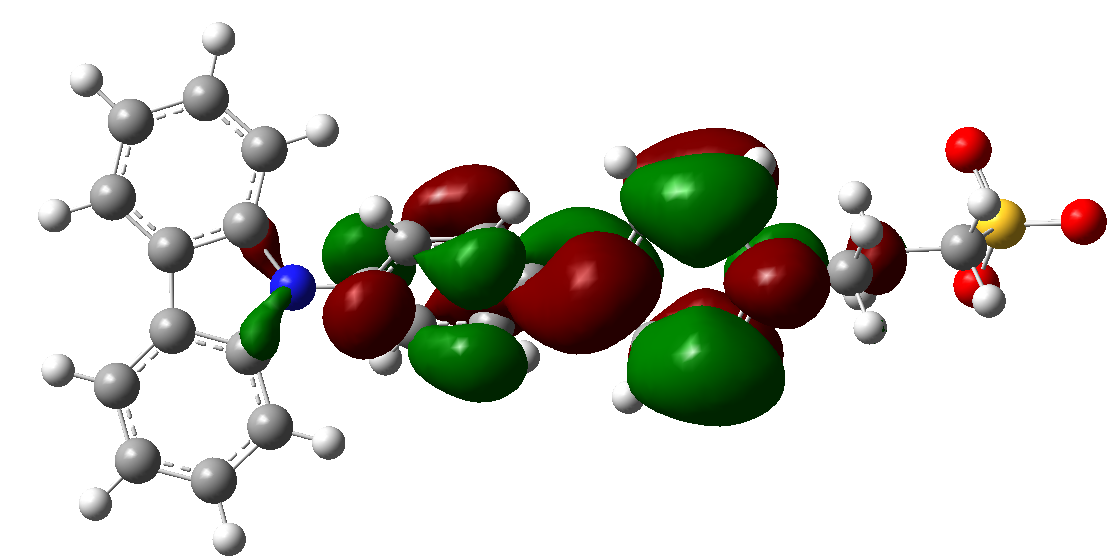 |
| POZ-PY | 2.82 | 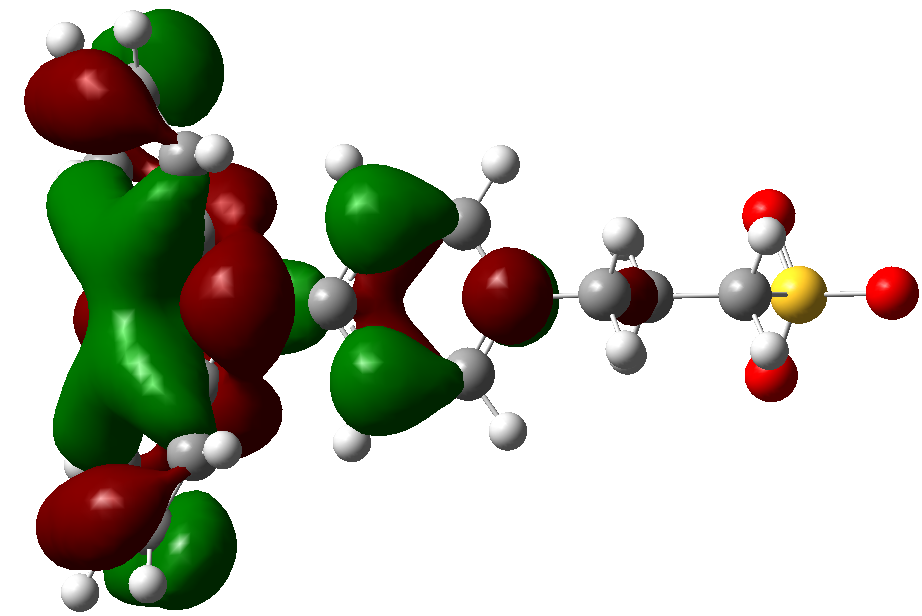 | 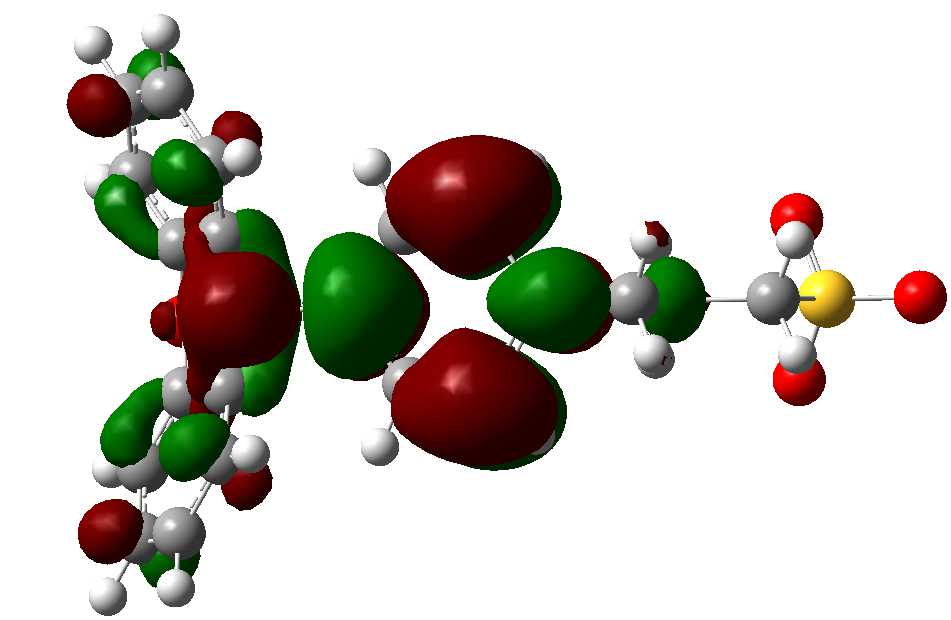 |
| POZ-B-PY | 6.91 | 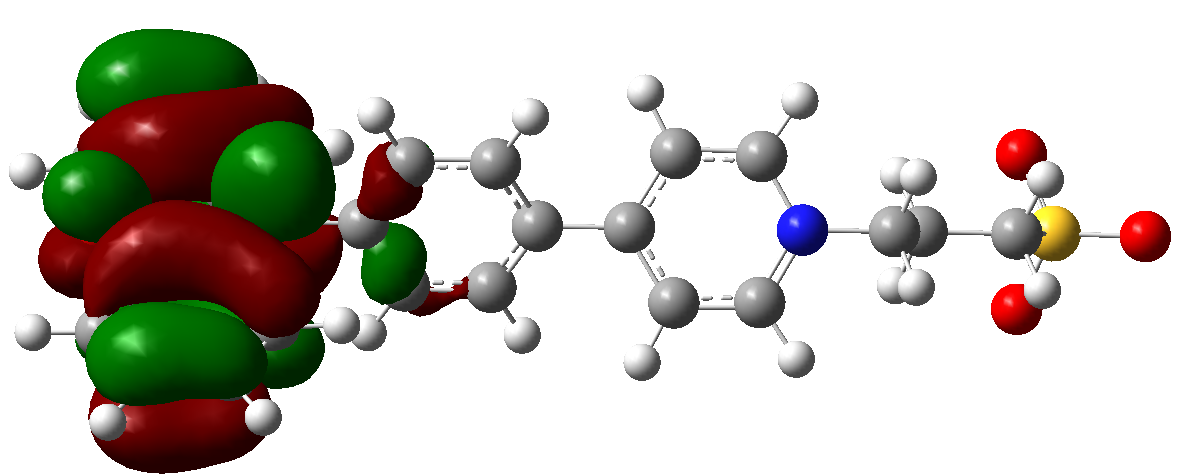 | 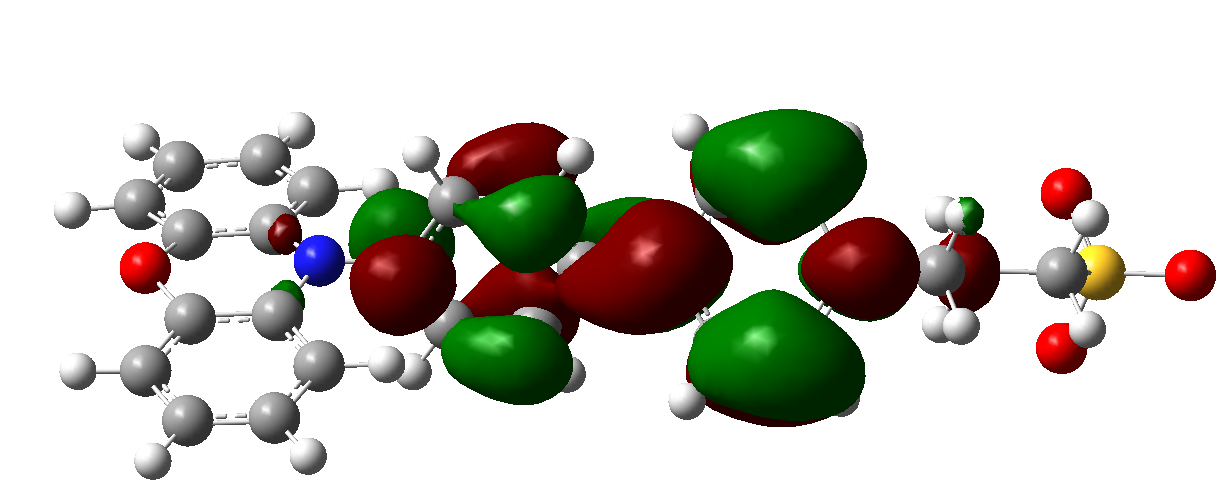 |
| POZ-T-PY | 5.51 | 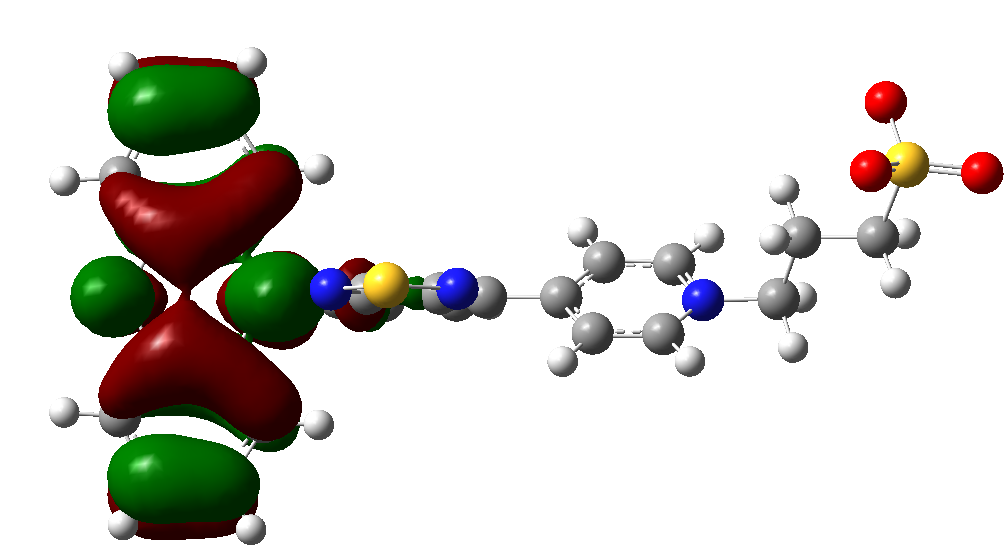 | 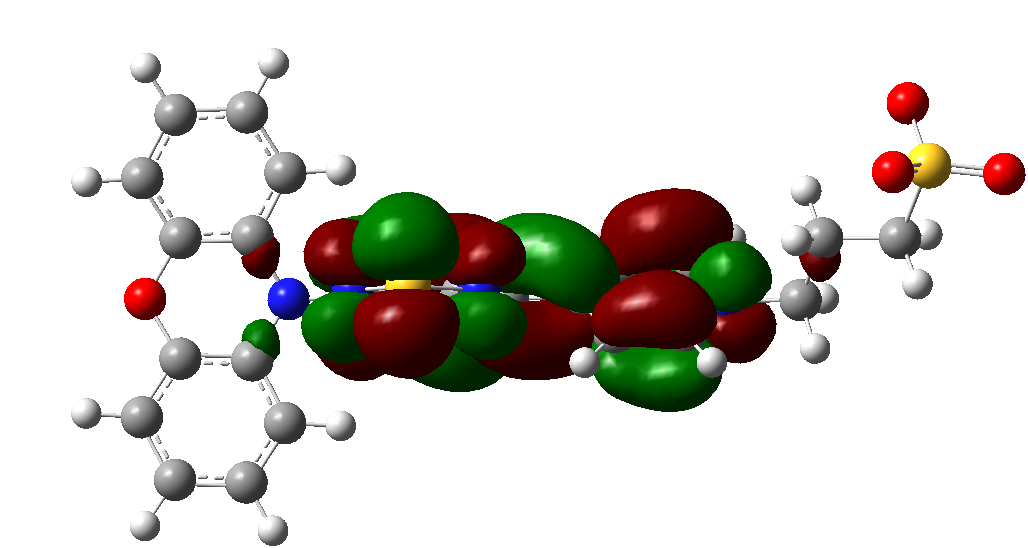 |
| POZ-BT-PY | 6.15 | 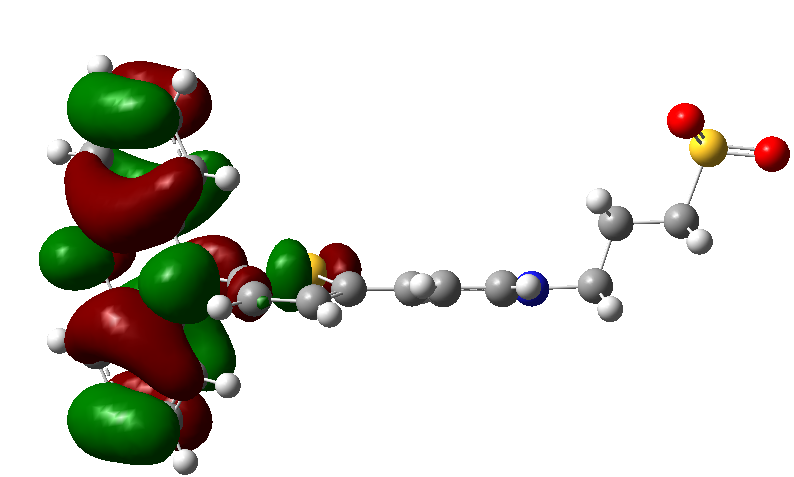 | 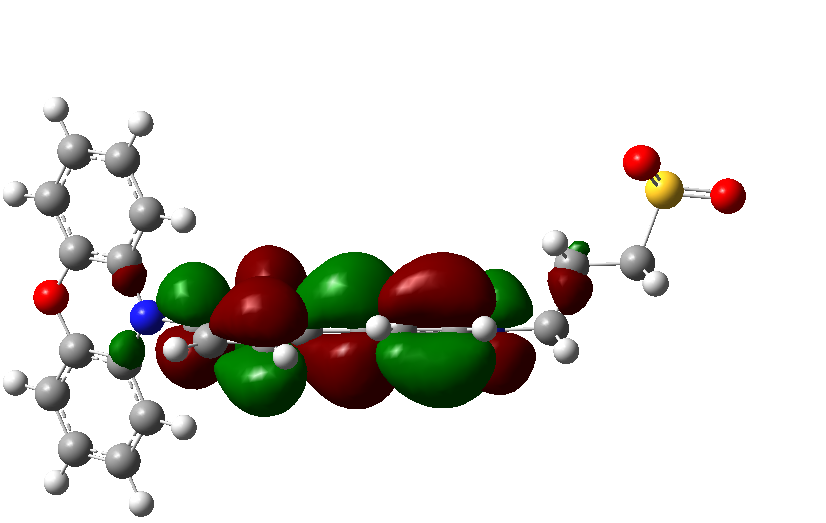 |

**Figure S1.** Frontier molecular orbitals and charge transfer lengths of the targeted PY compounds.

1. Top view

| 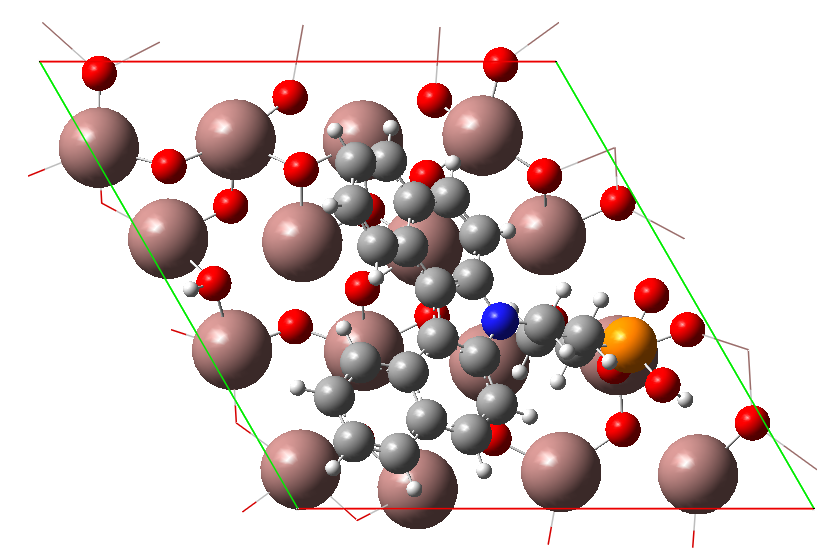 | 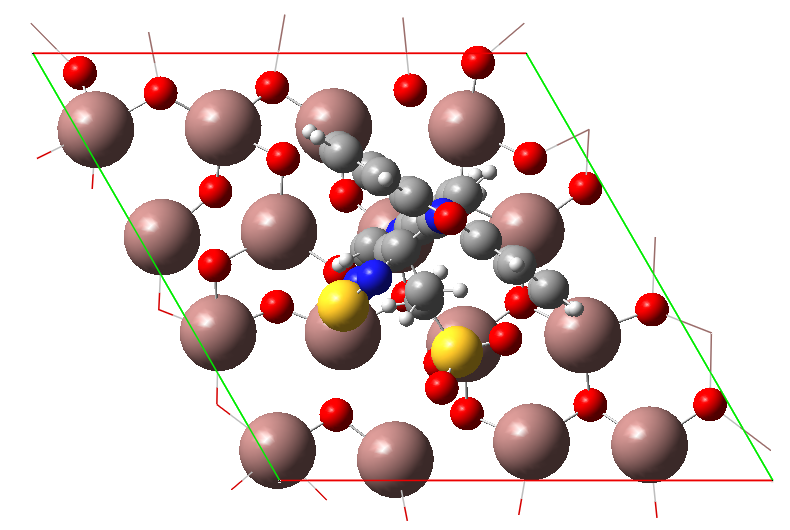 | 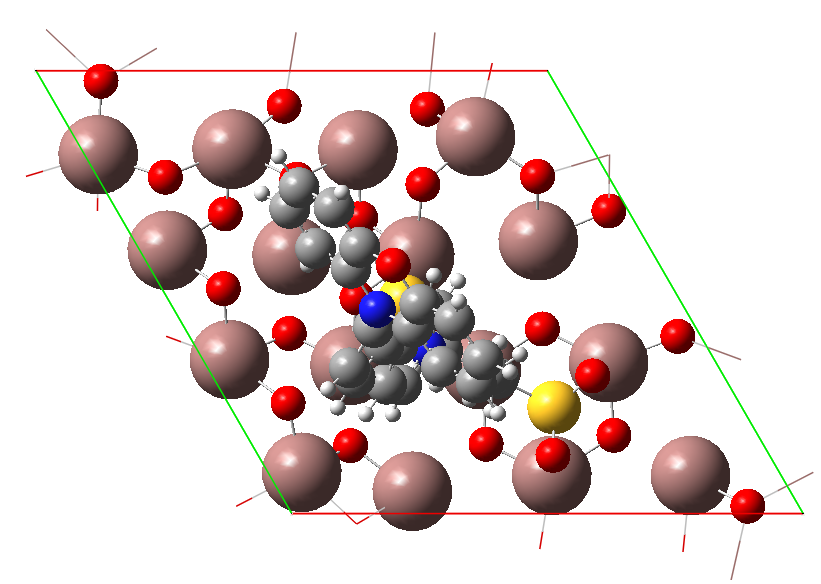 | 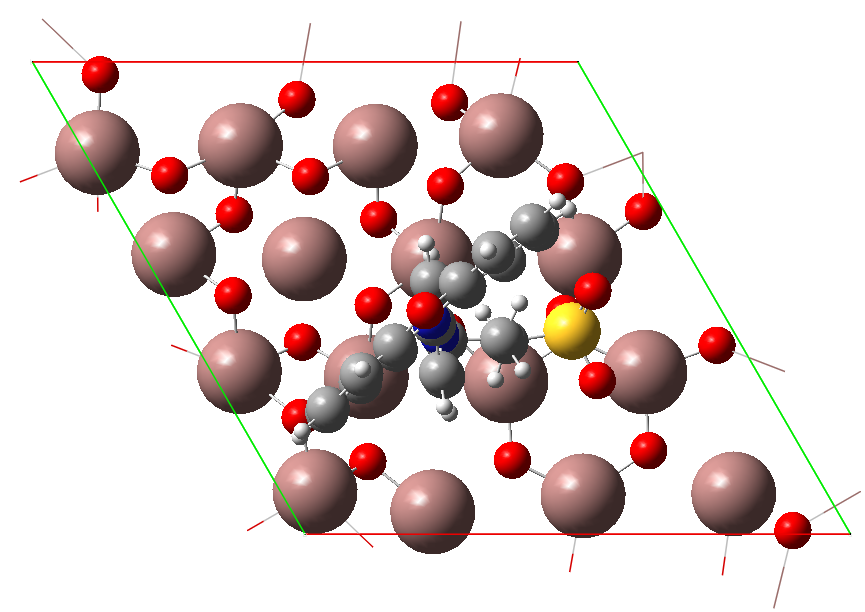 |
| --- | --- | --- | --- |
| CNph | POZ-BT-PY | POZ-T-PY | POZ-PY |
| 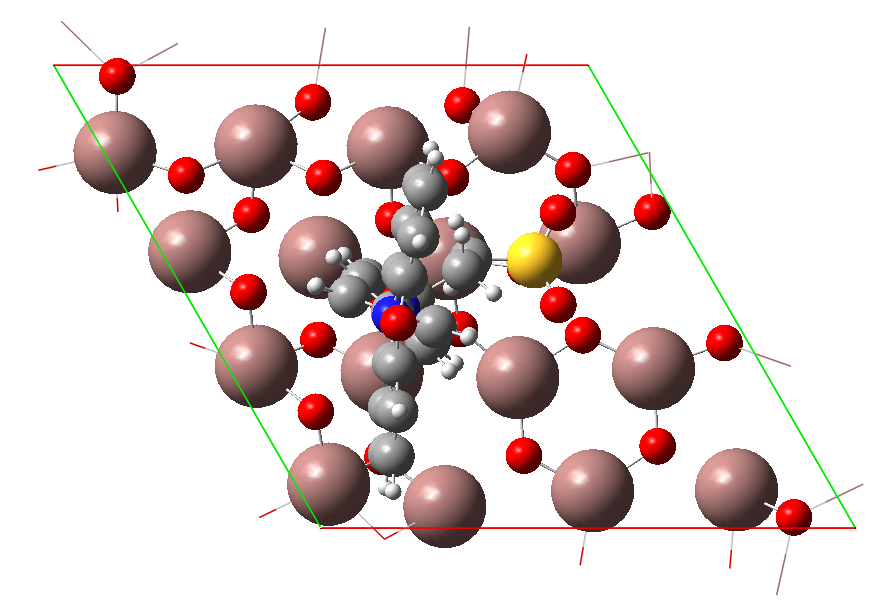 | 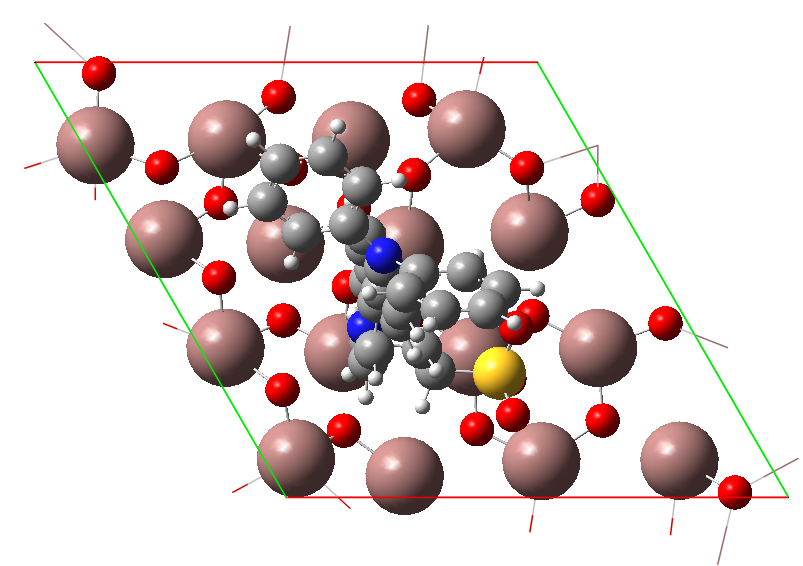 | 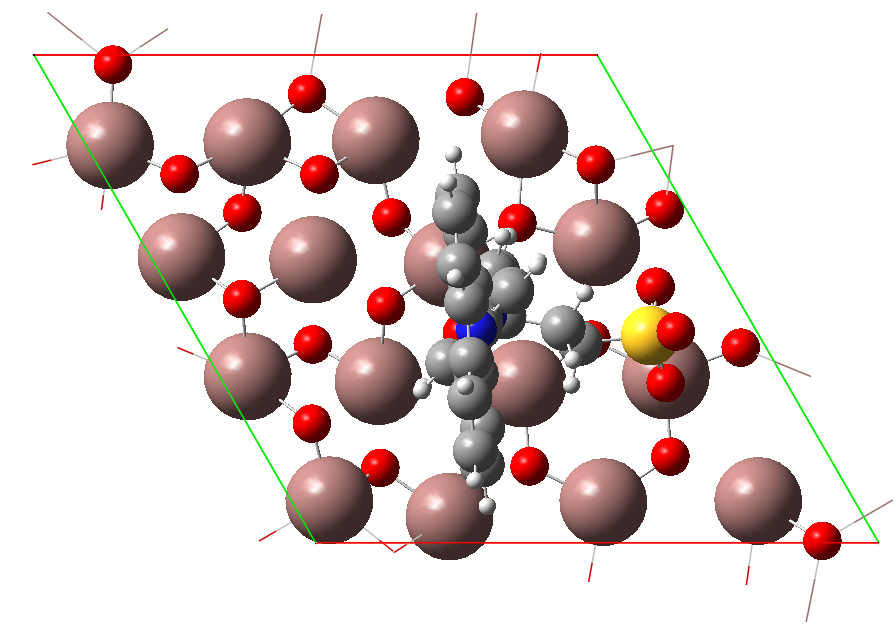 |  |
| POZ-B-PY | DPA-B-PY | CBZ-B-PY |  |

(b) Side view

| 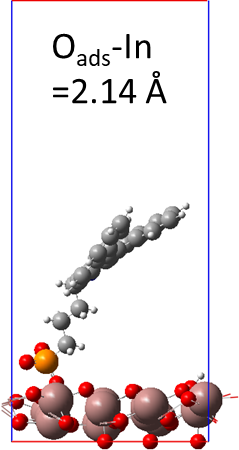 | 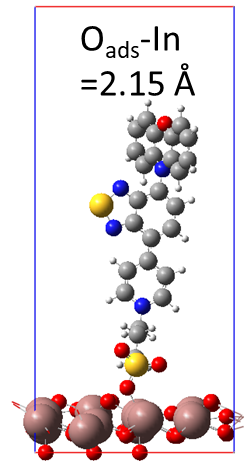 | 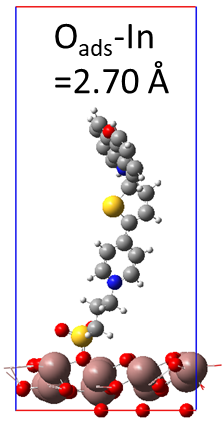 | 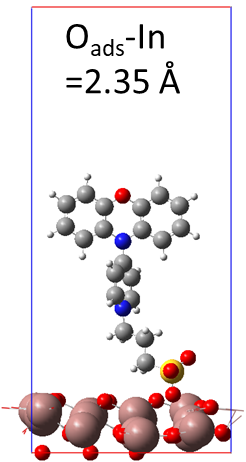 |
| --- | --- | --- | --- |
| CNph | POZ-BT-PY | POZ-T-PY | POZ-PY |
| 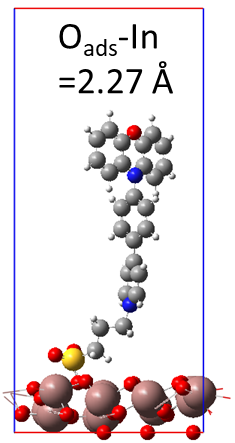 | 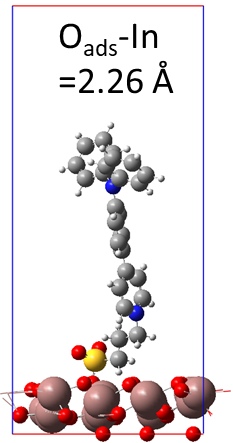 | 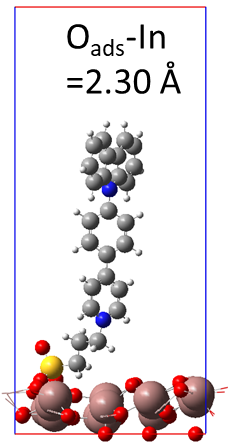 |  |
| POZ-B-PY | DPA-B-PY | CBZ-B-PY |  |

**Figure S2.** (a) Top view of SAM adsorption geometries. (b) Side view and distance between the oxygen atom (O) of the sulfonate/phosphonic acid (O_ads_) and the indium atom (In) on the ITO surface.

**Table S1.** Dihedral angles between the donor and acceptor of the targeted PY compounds.


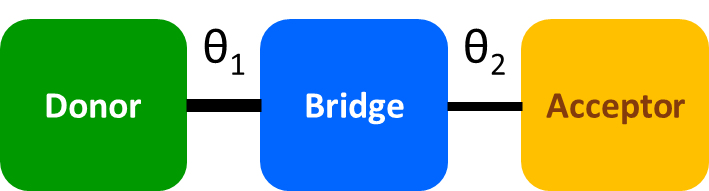

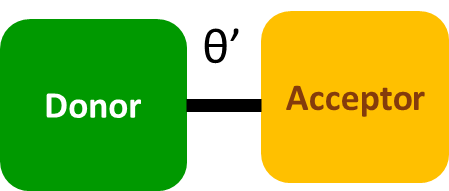


|  | θ_1_ (˚) | θ_2_ (˚) | θ’ (˚) |
| --- | --- | --- | --- |
| DPA-B-PY | 26.99 | 30.64 |  |
| CBZ-B-PY | 54.30 | -36.34 |  |
| POZ-PY |  |  | 13.88 |
| POZ-B-PY | -78.13 | -37.56 |  |
| POZ-T-PY | 81.97 | -16.51 |  |
| POZ-BT-PY | -76.14 | 38.70 |  |

**Table S2.** Adsorption energies of the systems concerned, along with the energies of the substrate with adsorbate, the surface, and the adsorbate.

| Adsorbate | *E*_Adsorbate_ (eV) | *E*_Surface_ (eV) | *E*_System_ (eV) | *E*_ads_ (eV) | PCE (%) |
| --- | --- | --- | --- | --- | --- |
| POZ-T-PY | -338.99 | -188.50 | -528.89 | -1.39 | 23.93 |
| POZ-B-PY | -360.67 |  | -550.88 | -1.71 | 23.67 |
| POZ-PY | -291.75 |  | -481.77 | -1.52 | 23.72 |
| POZ-BT-PY | -373.16 |  | -562.17 | -0.50 | 24.45 |
| CBZ-B-PY | -354.96 |  | -545.97 | -2.51 | 22.5 |
| CNph | -334.34 |  | -525.35 | -2.51 | 23.0 |
| DPA-B-PY | -361.74 |  | -552.34 | -2.10 | 23.25 |


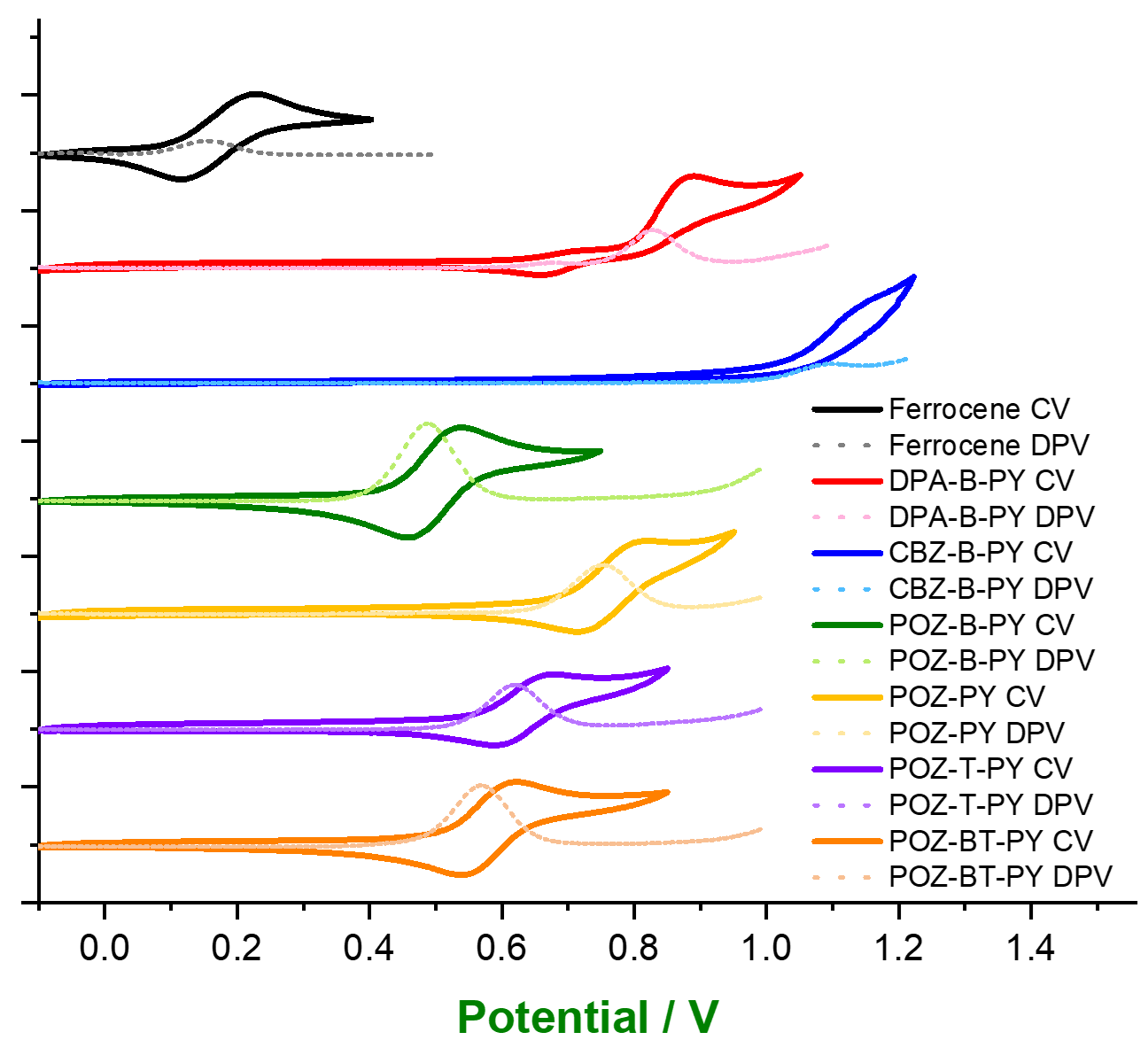


**Figure S3.** CVs (bold solid lines) and DPVs (thin dotted lines) of PY-series compounds in THF/0.1 M TBAP under nitrogen.


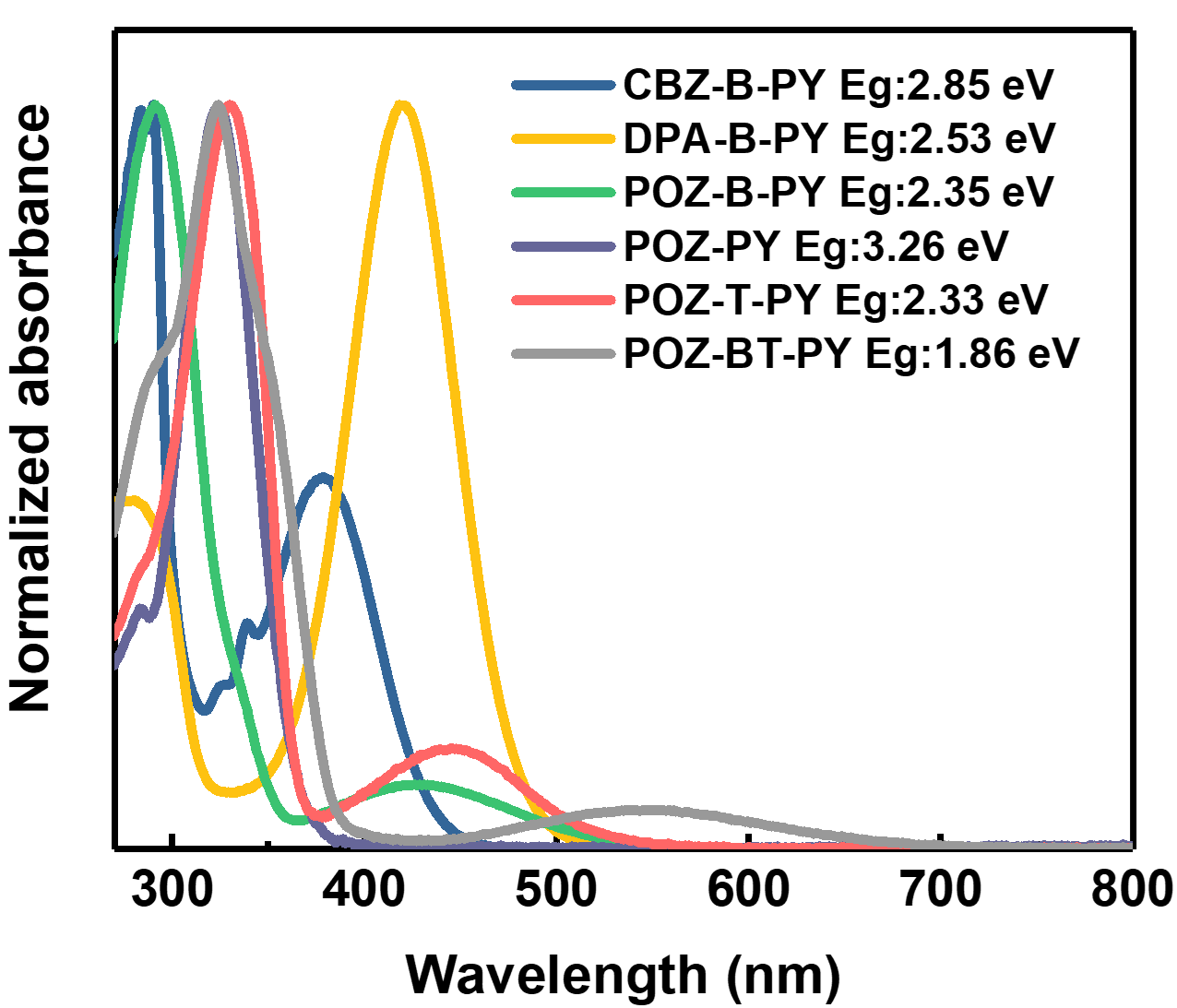


**Figure S4.** UV−vis absorption spectra of PY-series compounds in DMF solution.


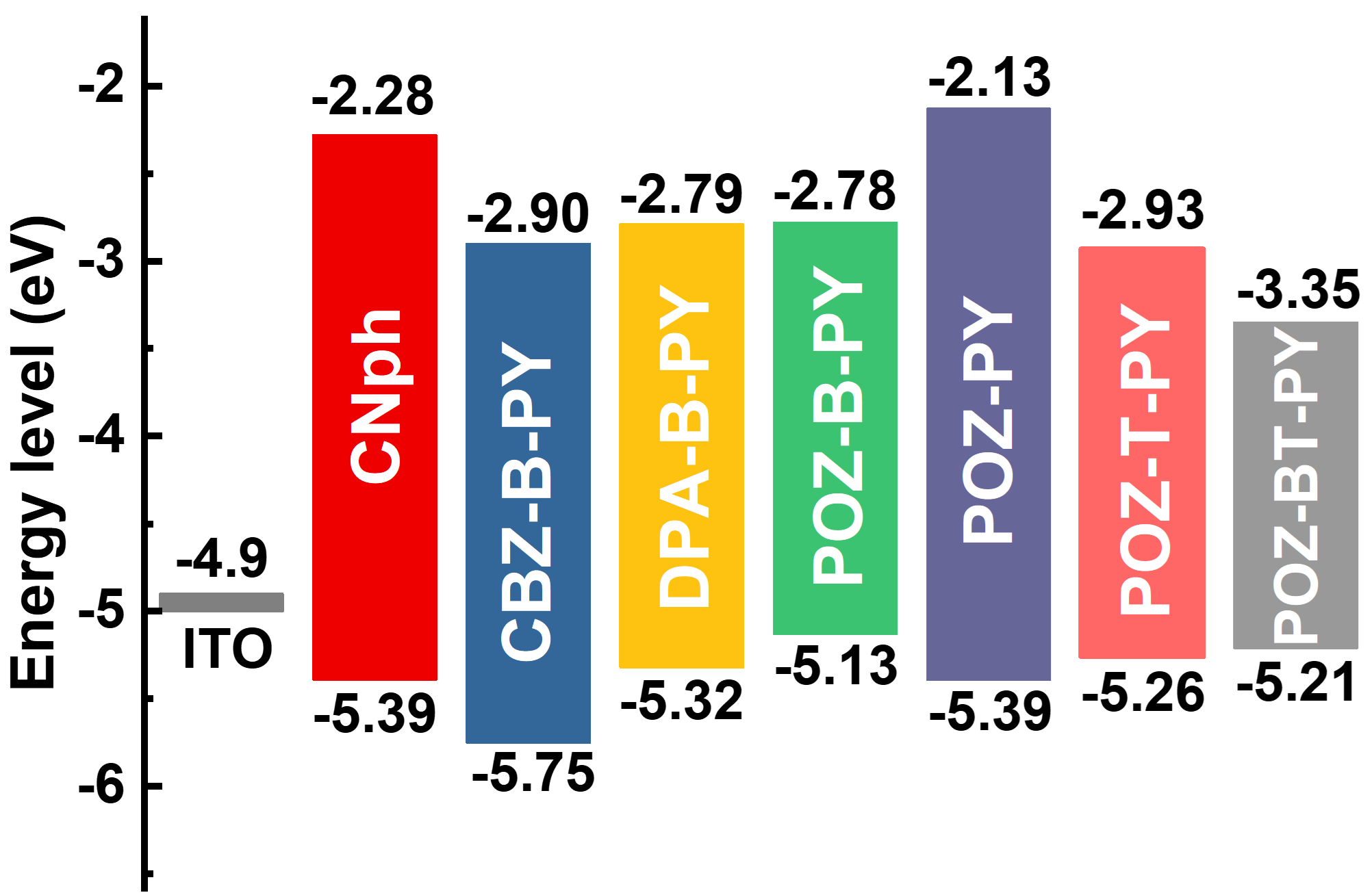


**Figure S5.** Energy level diagram of different compositions in PSCs.


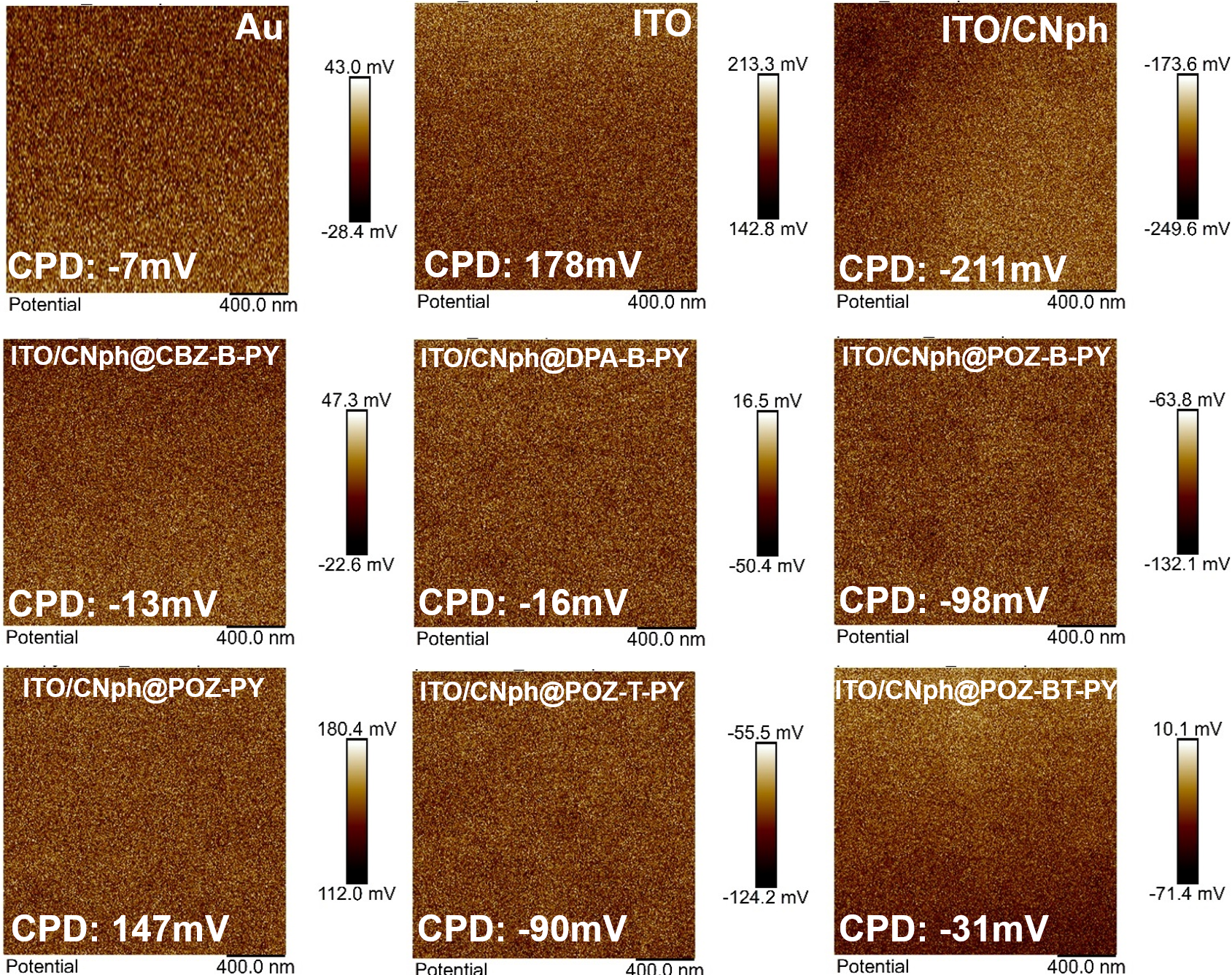


**Figure S6.** KPFM images of Au, bare ITO, ITO/CNph, and ITO/CNph@PY-series substrates.

**Table S3.** CPD and WF of Au, bare ITO, and SAM-modified films.

| Compounds | CPD (meV) | Work Function (eV)^[a]^ |
| --- | --- | --- |
| Au | -7 | 5.100 |
| ITO | 178 | 4.915 |
| ITO/CNph | -211 | 5.304 |
| ITO/CNph@CBZ-B-PY | -13 | 5.106 |
| ITO/CNph@DPA-B-PY | -16 | 5.109 |
| ITO/CNph@POZ-B-PY | -98 | 5.191 |
| ITO/CNph@POZ-PY | 147 | 4.946 |
| ITO/CNph@POZ-T-PY | -90 | 5.183 |
| ITO/CNph@POZ-BT-PY | -31 | 5.124 |

^[a]^The work function (E_f_) of thin films can be calculated according to Equations: CPD = (W_tip_ − W_f_)/e, W_f_ = 5.1 + e(CPD_Au_ − CPD_sample_), E_f_ = −W_f_, W_tip_ and W_f_ are the work functions of the probe tip and the sample surface, respectively, e is the charge of the electron, and E_f_ is the fermi level of perovskite. Normally, the W_tip_ value of the tip was calibrated by the Au sample with W_Au_ = 5.1 eV and CPD_Au_ = −7 mV.


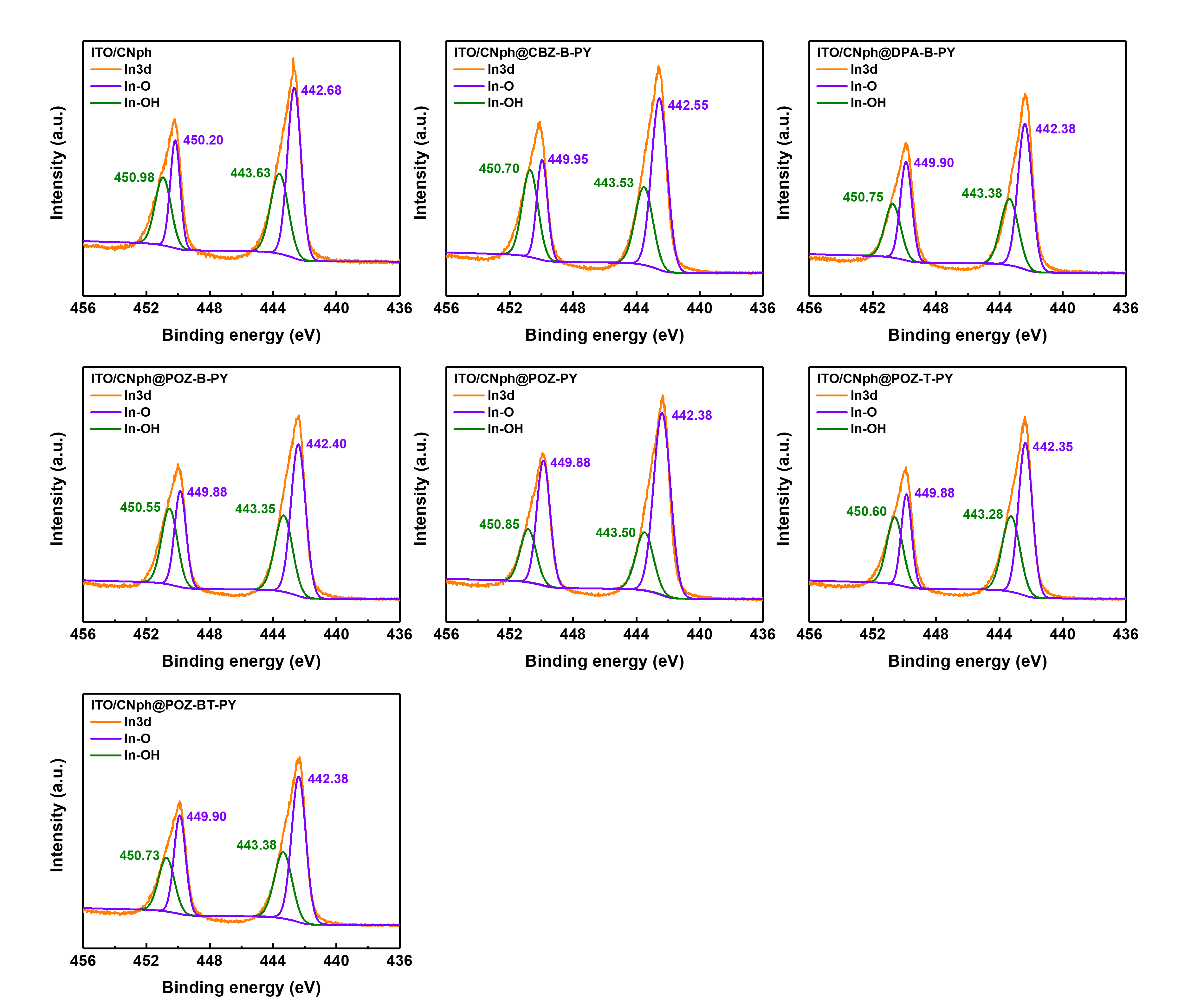


**Figure S7.** XPS spectra of the core-level In 3d element of ITO/CNph and ITO/CNph@PY-series films. The fitted data is represented by the orange line, the characteristic peak of In-O is depicted by the purple line, and the characteristic peak of In-OH is shown by the green line.


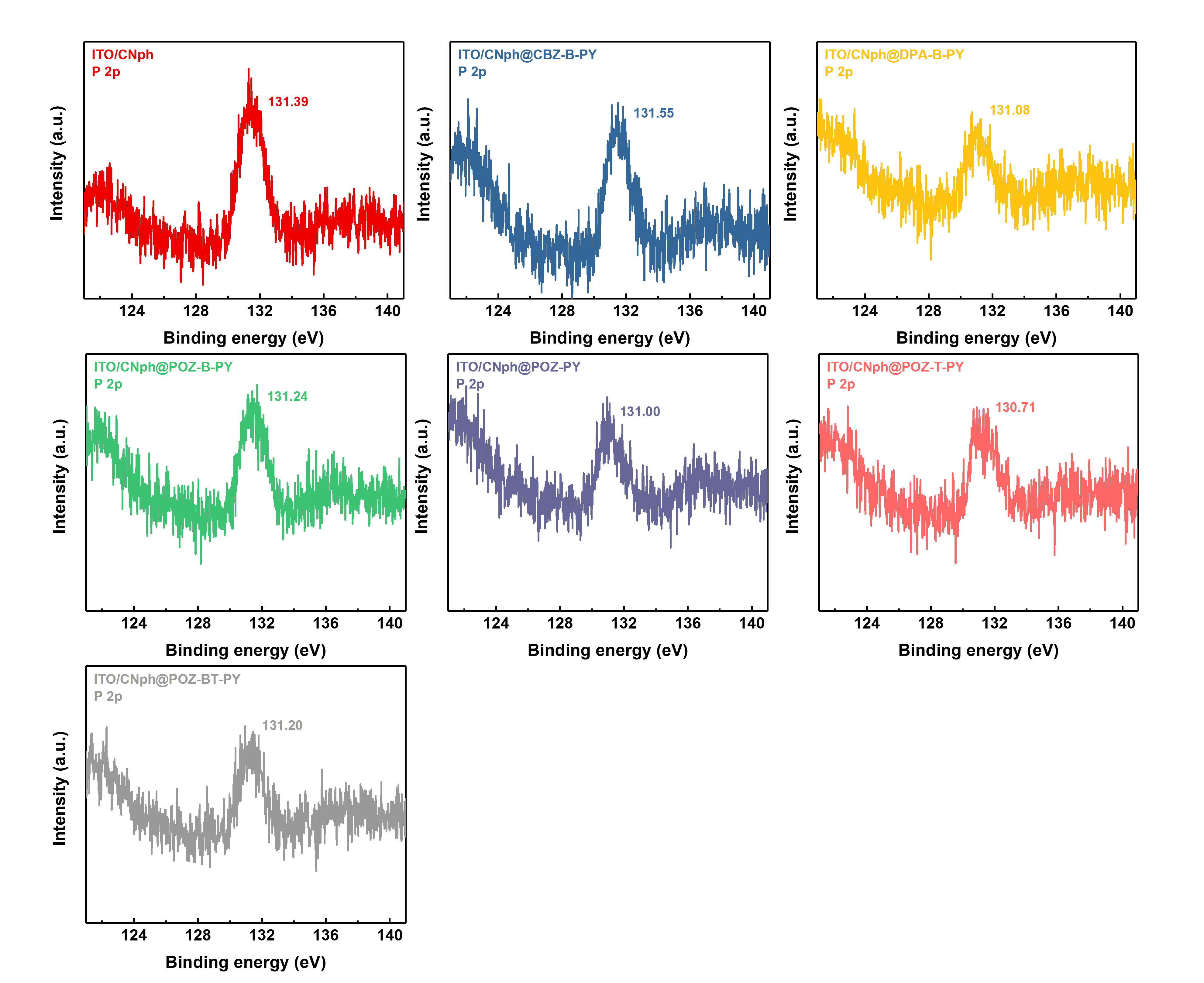


**Figure S8.** XPS spectra of the core-level P 2p element of ITO/CNph and ITO/CNph@PY-series films.


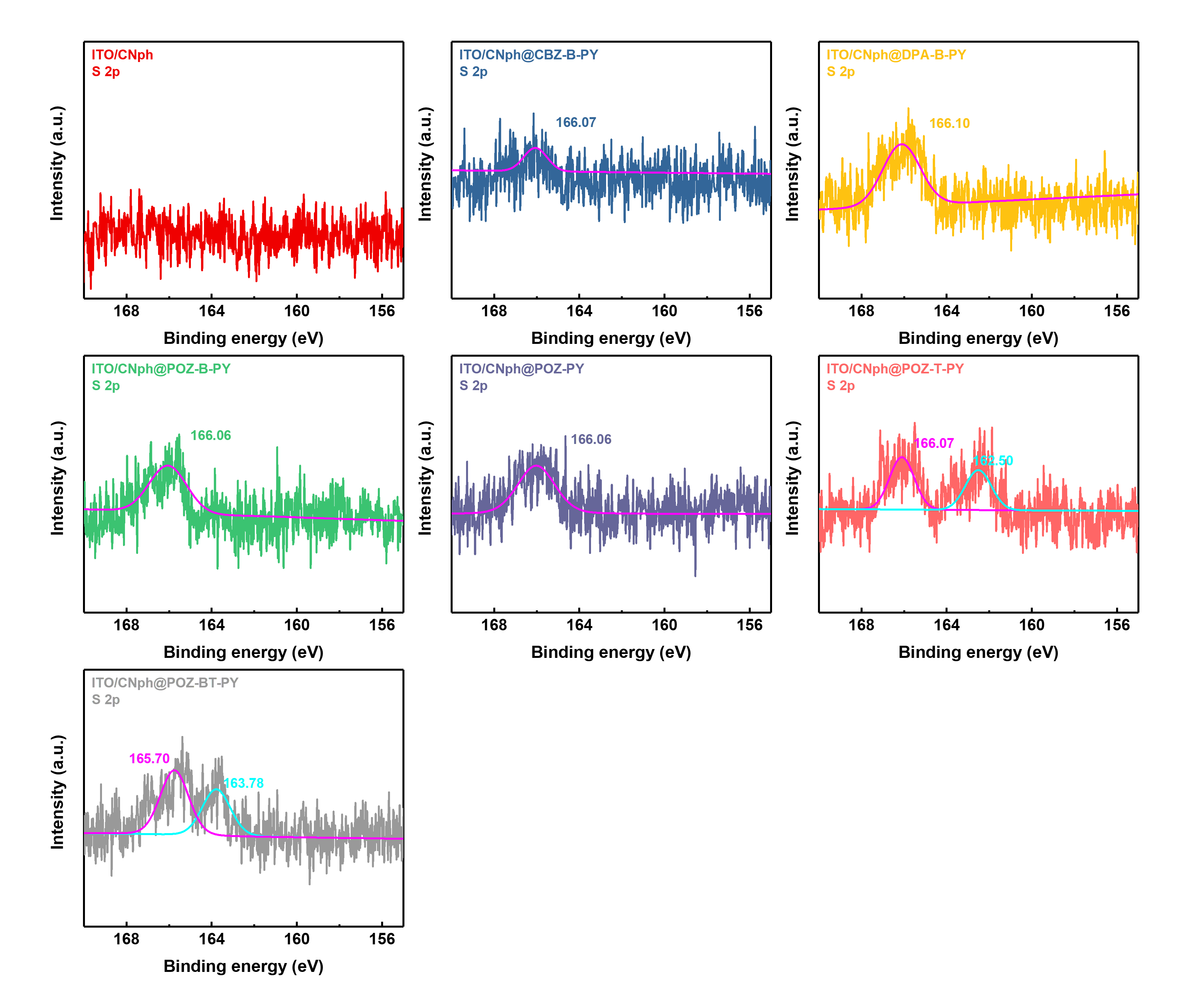


**Figure S9.** XPS spectra of the core-level S 2p element of ITO/CNph and ITO/CNph@PY-series films.


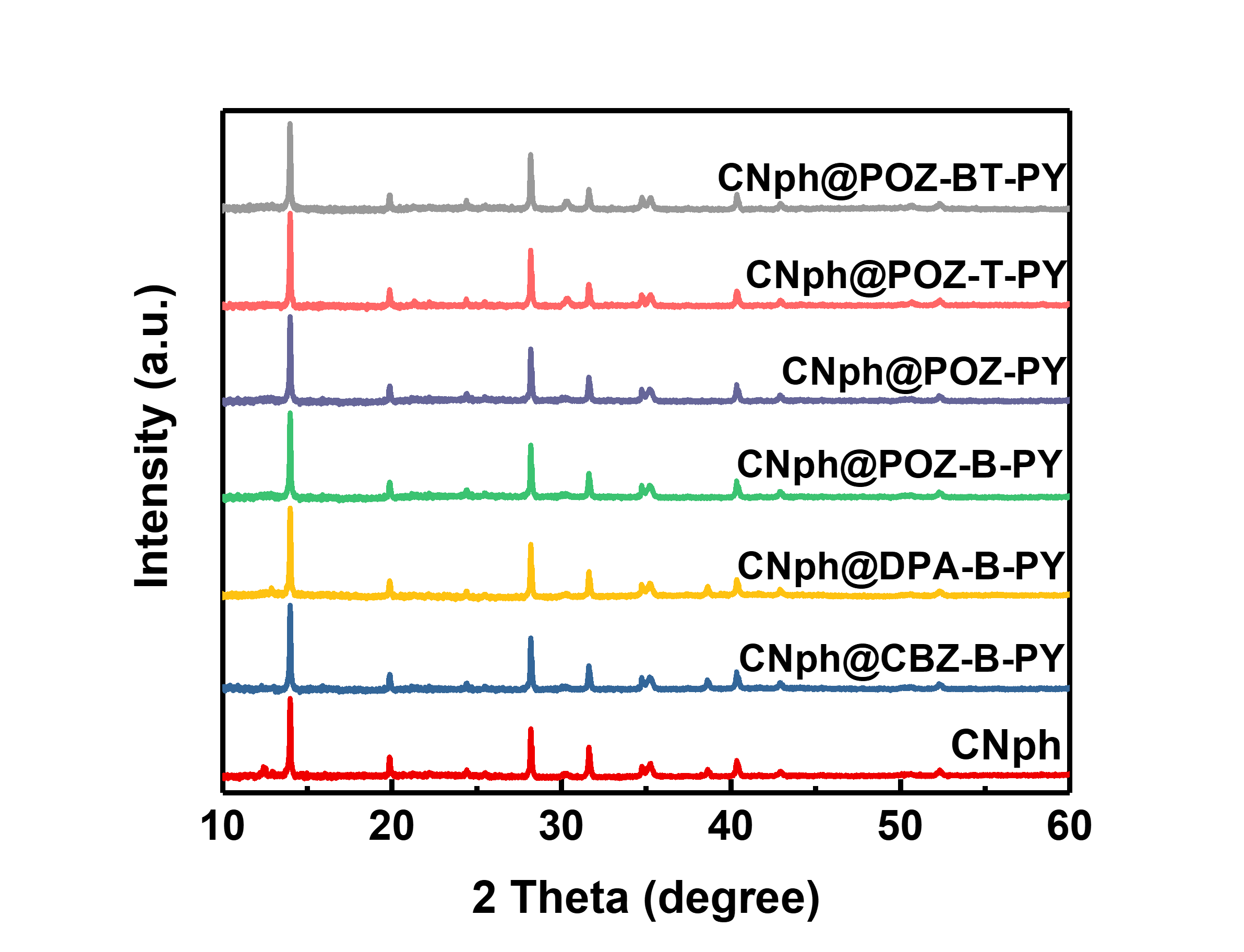


**Figure S10.** XRD patterns of the SAM-modified perovskite films.


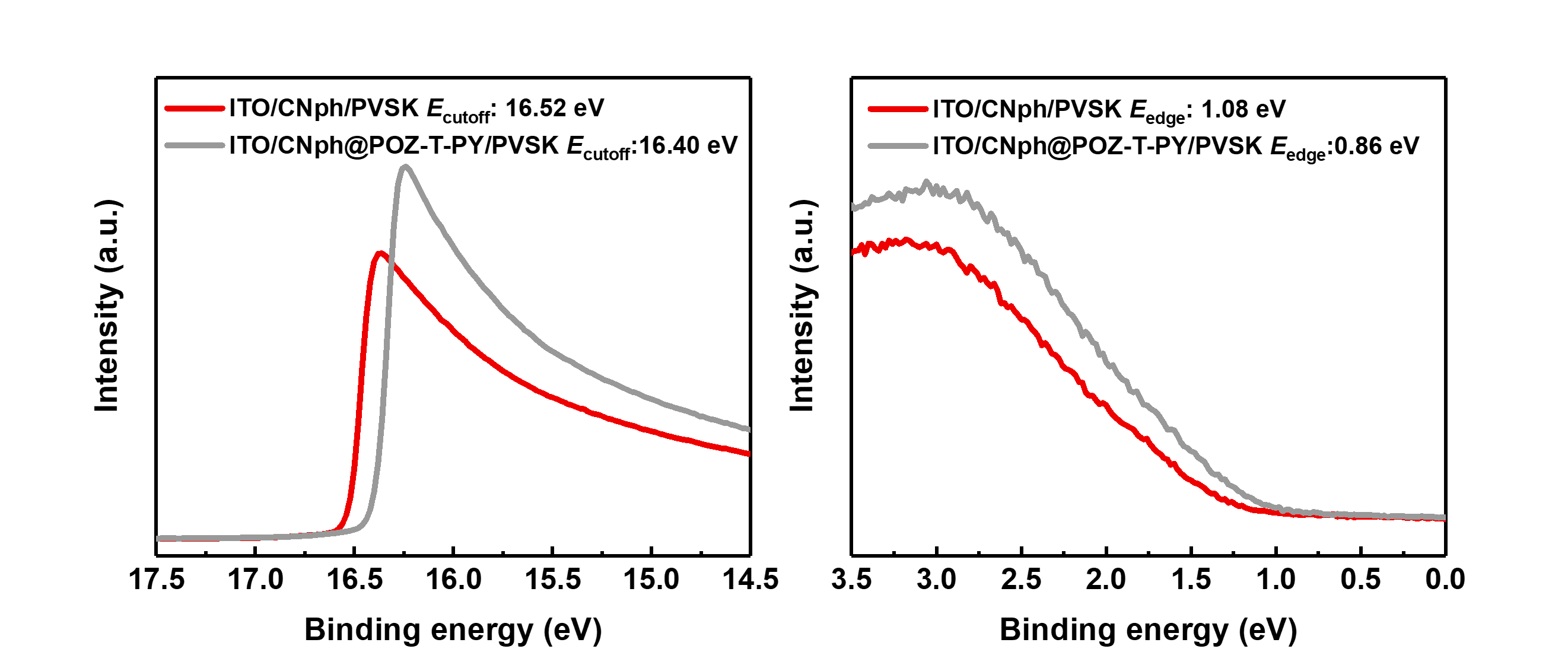


**Figure S11.** UPS spectra of ITO/CNph/perovskites and ITO/CNph@POZ-BT-PY/perovskites samples.


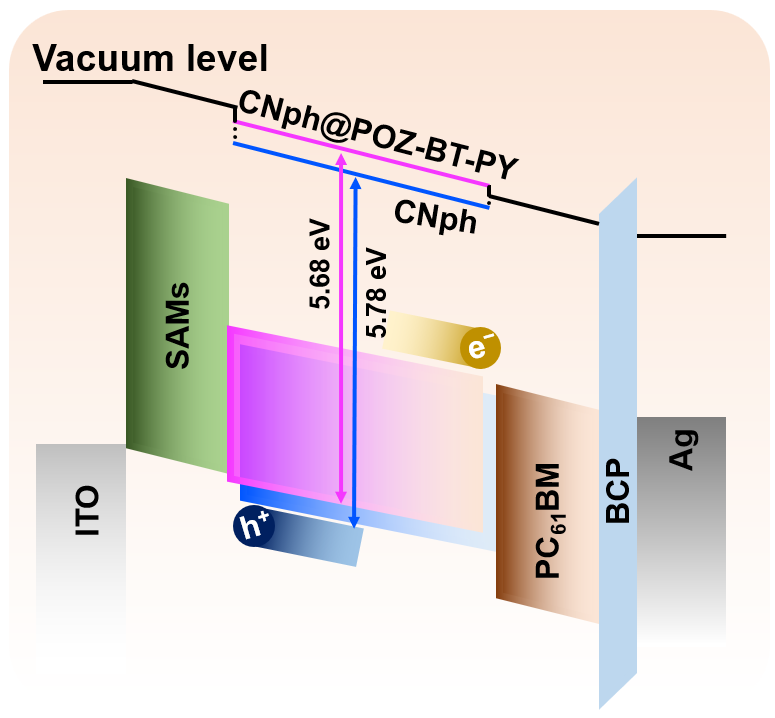


**Figure S12.** Schematic illustration of the energy levels of the individual functional layer in the perovskite photovoltaic device.


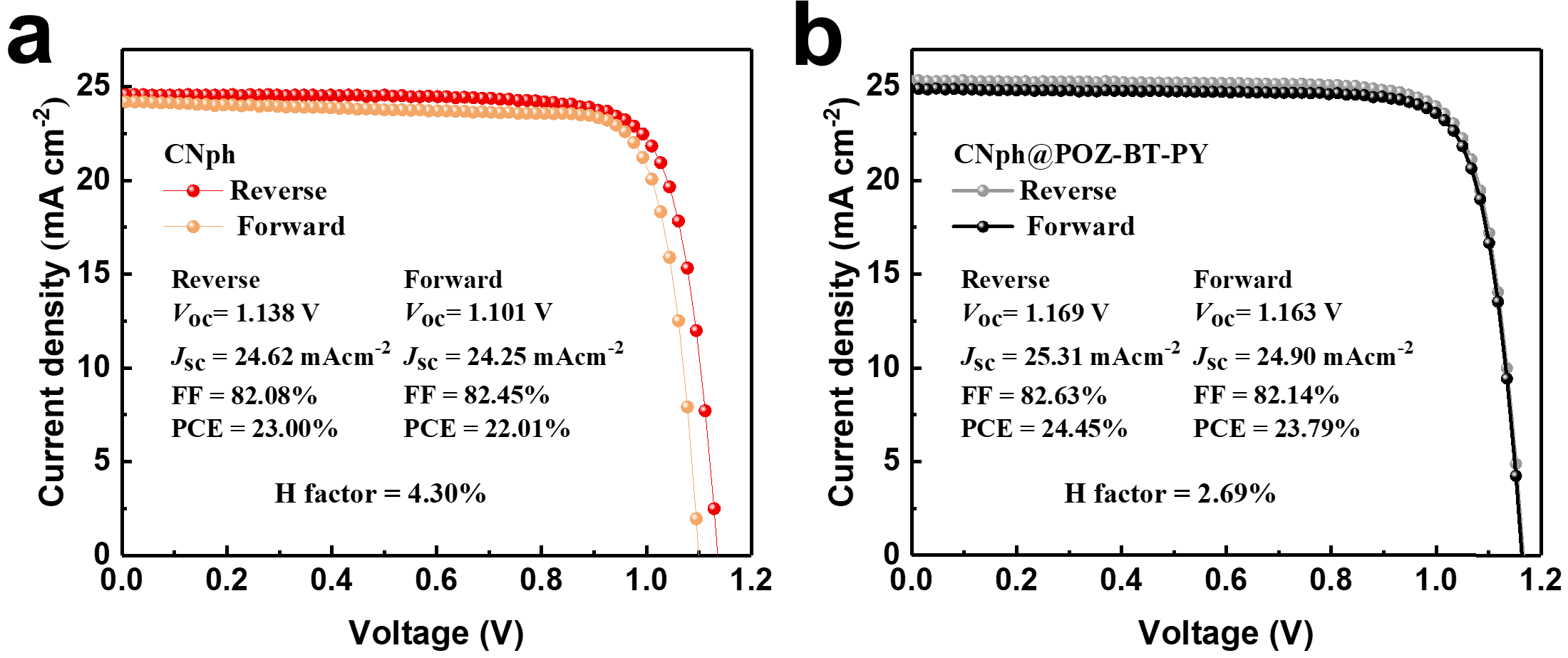


**Figure S13.** *J–V* curves of the champion (a) CNph-based and (b) CNph@POZ-BT-PY-modified PSCs with forward- and reverse-scan modes, and the corresponding photovoltaic parameters under 1 sun illumination.


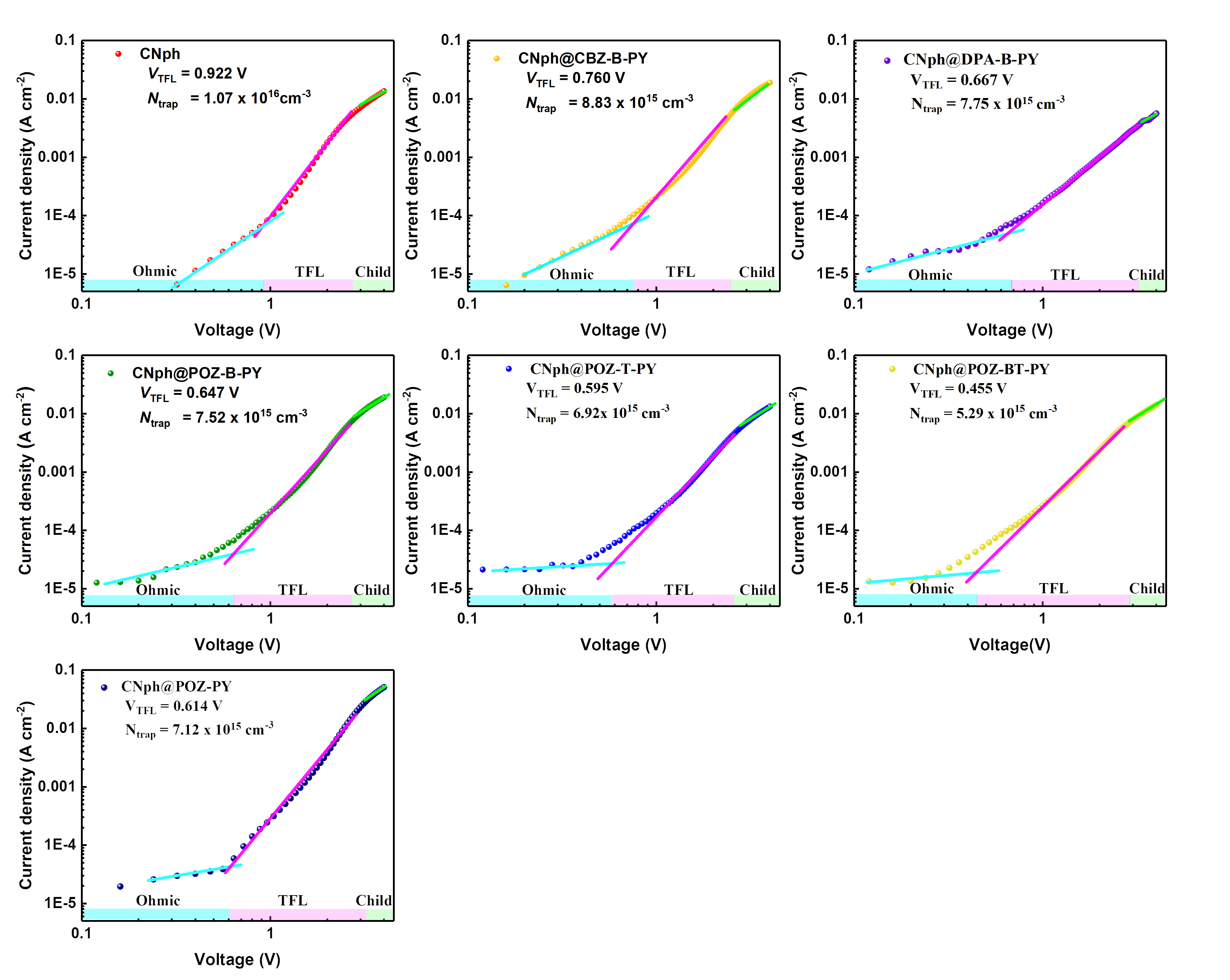


**Figure S14.** SCLC plots of CNph and CNph@PY-series modified perovskites based on hole- only devices.

**
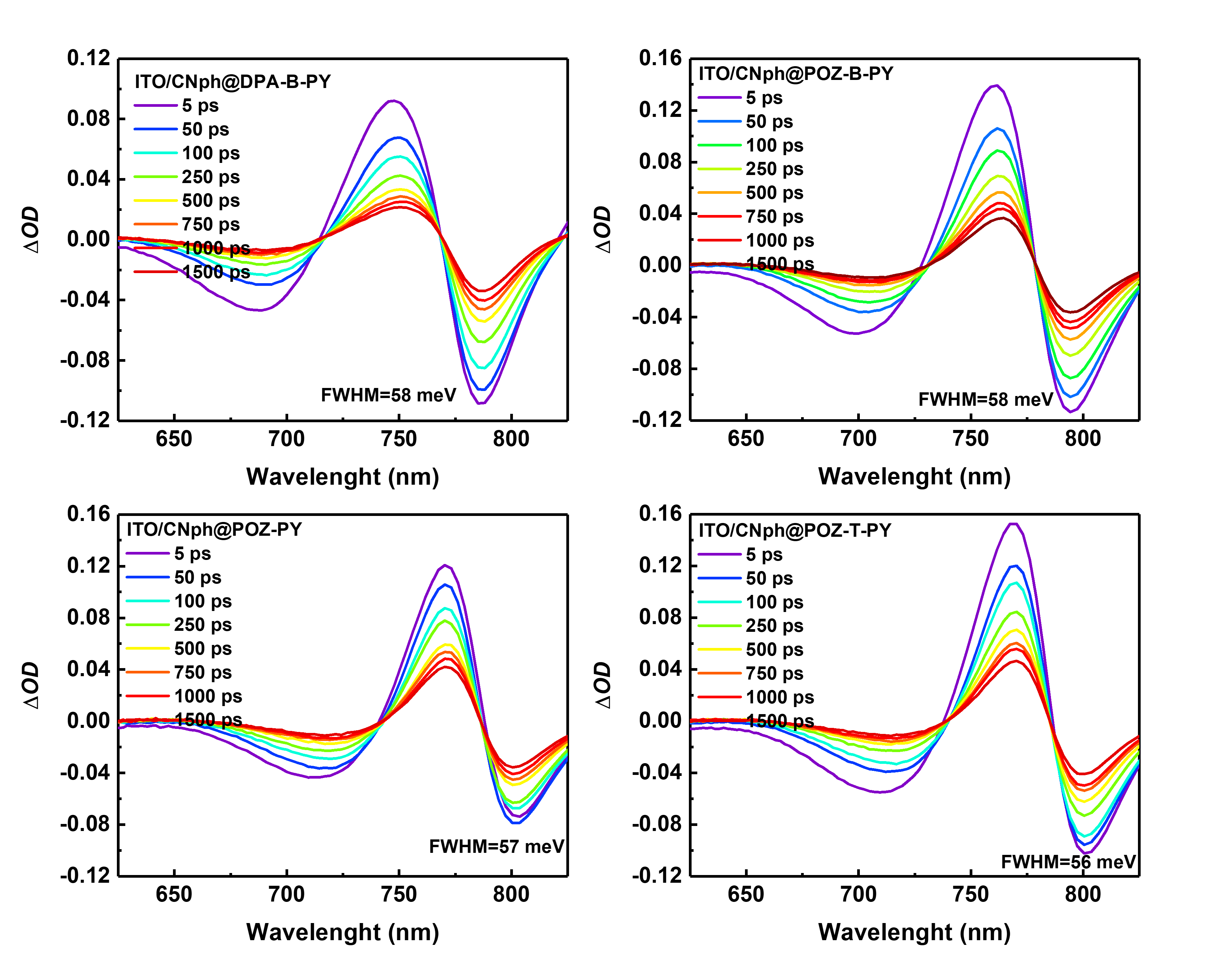
**

**Figure S15.** ps-TA spectra of perovskites atop CNph@PY-series SAM-coated ITO under front face excitation.

**
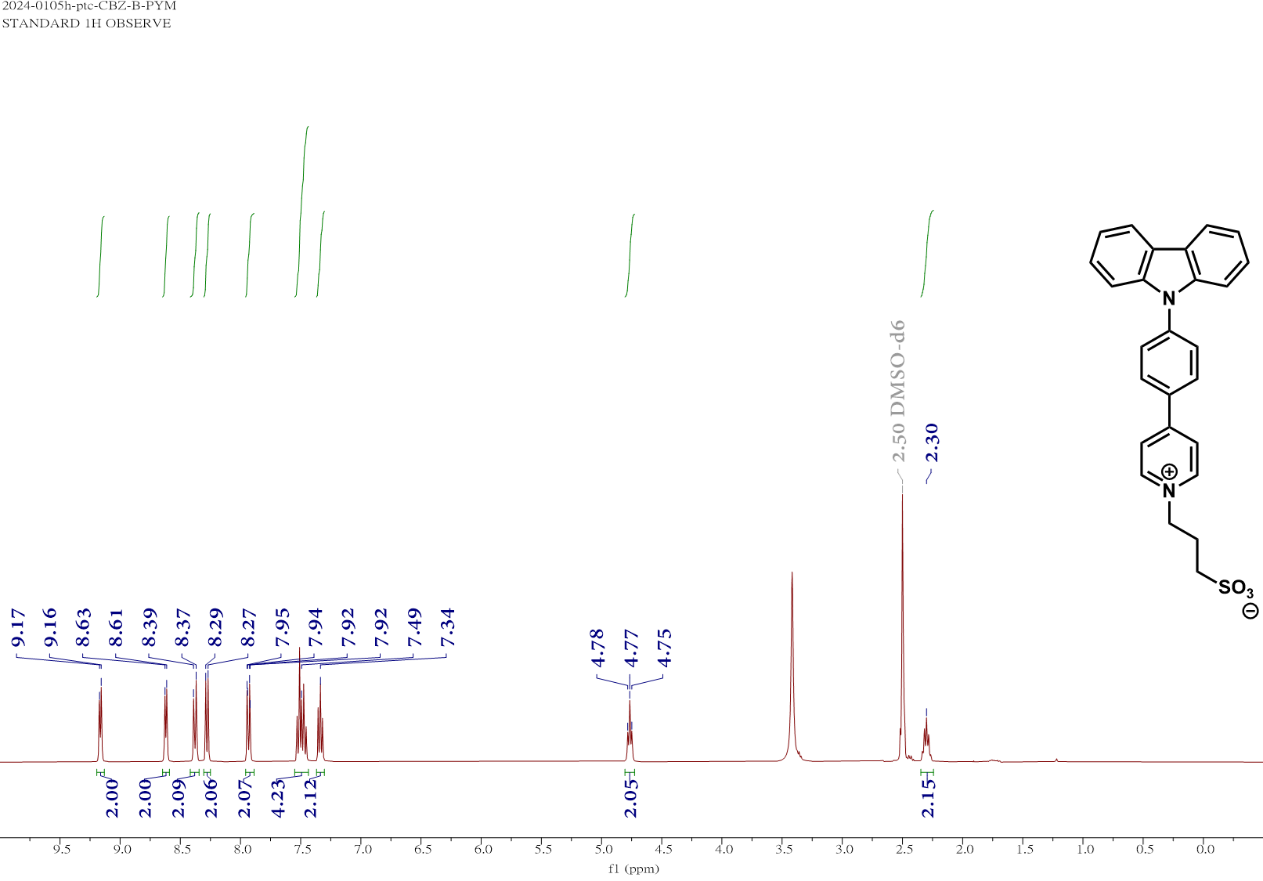
**

**Figure S16.** ^1^H NMR spectrum of CBZ-B-PY (DMSO-*d*_6_).

**
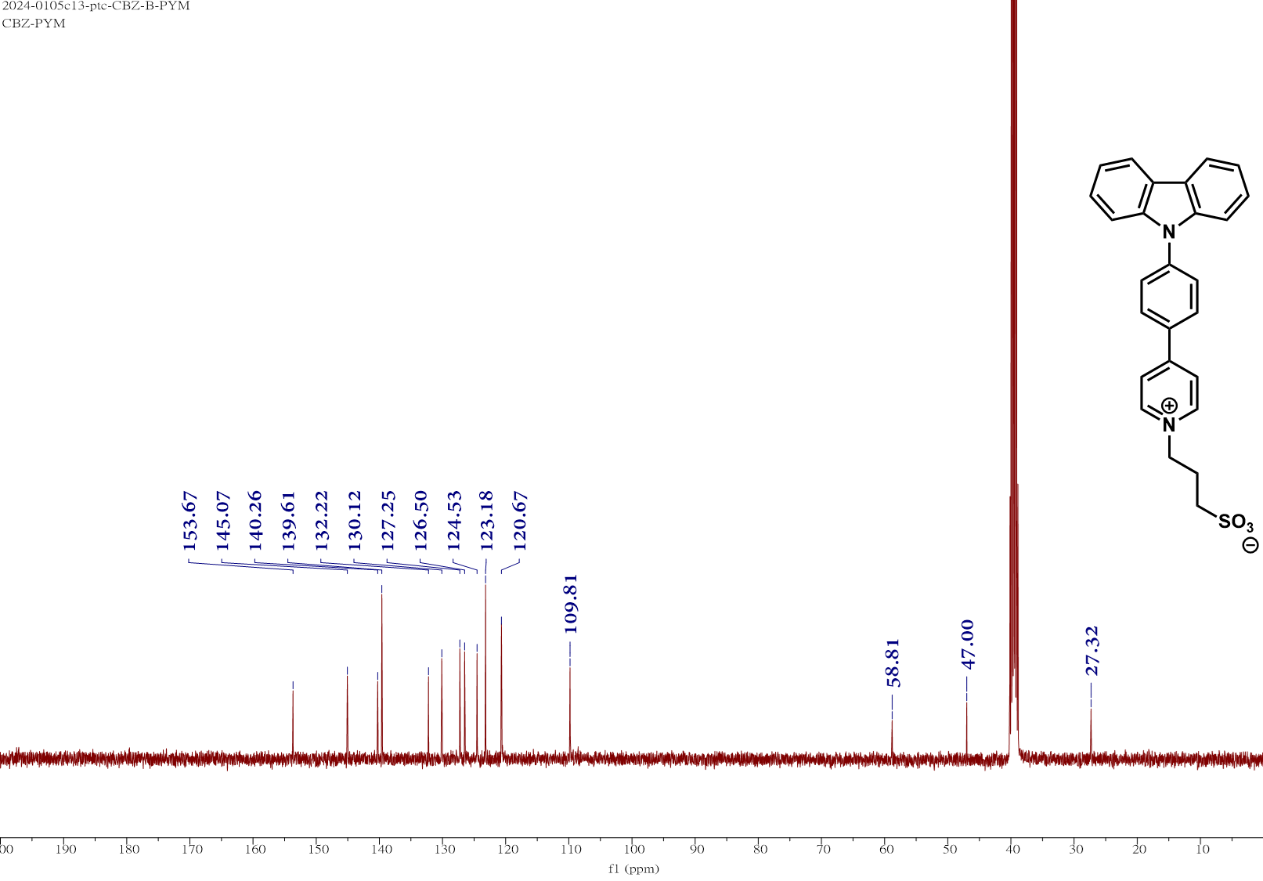
**

**Figure S17**. ^13^C NMR spectrum of **CBZ-B-PY** (DMSO-*d_6_*).


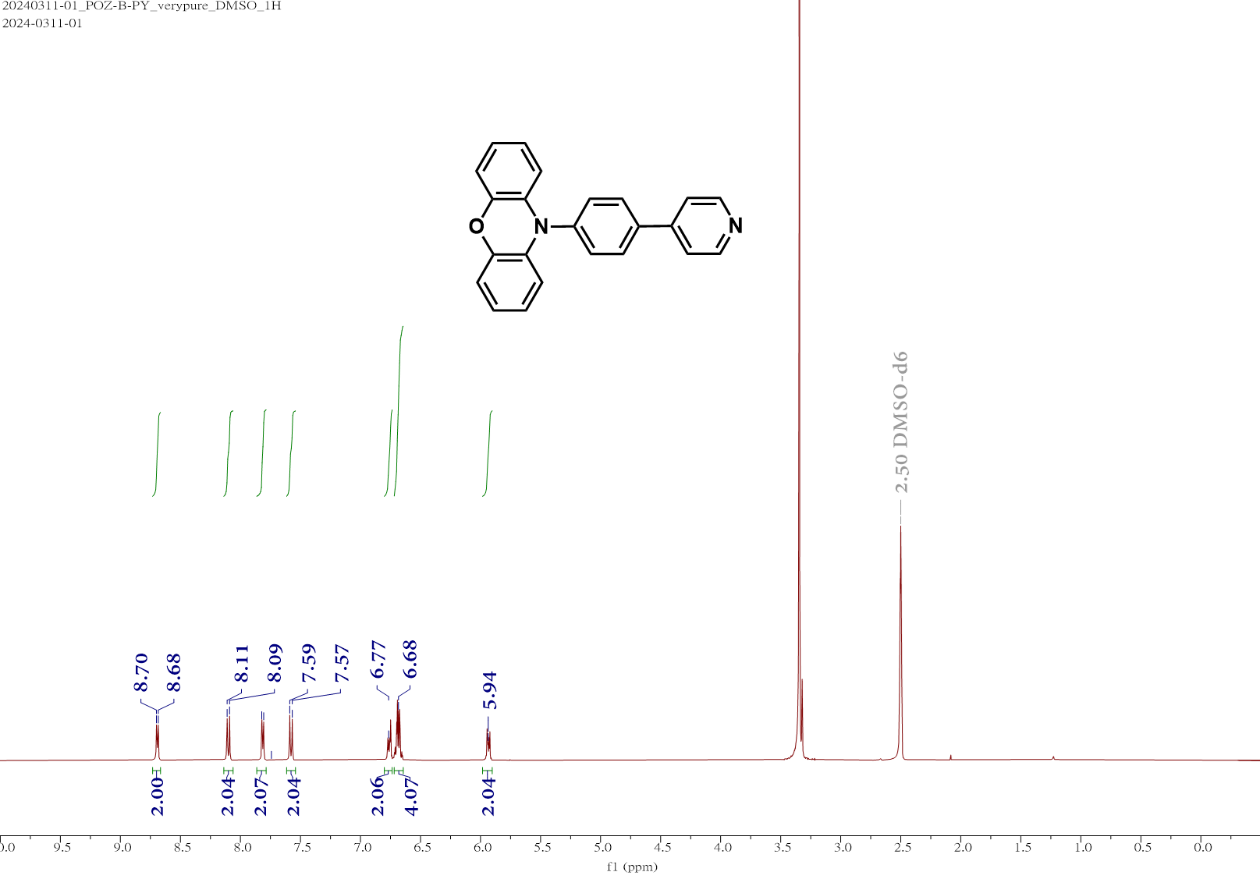


**Figure S18.** ^1^H NMR spectrum of **5** (DMSO-*d_6_*).


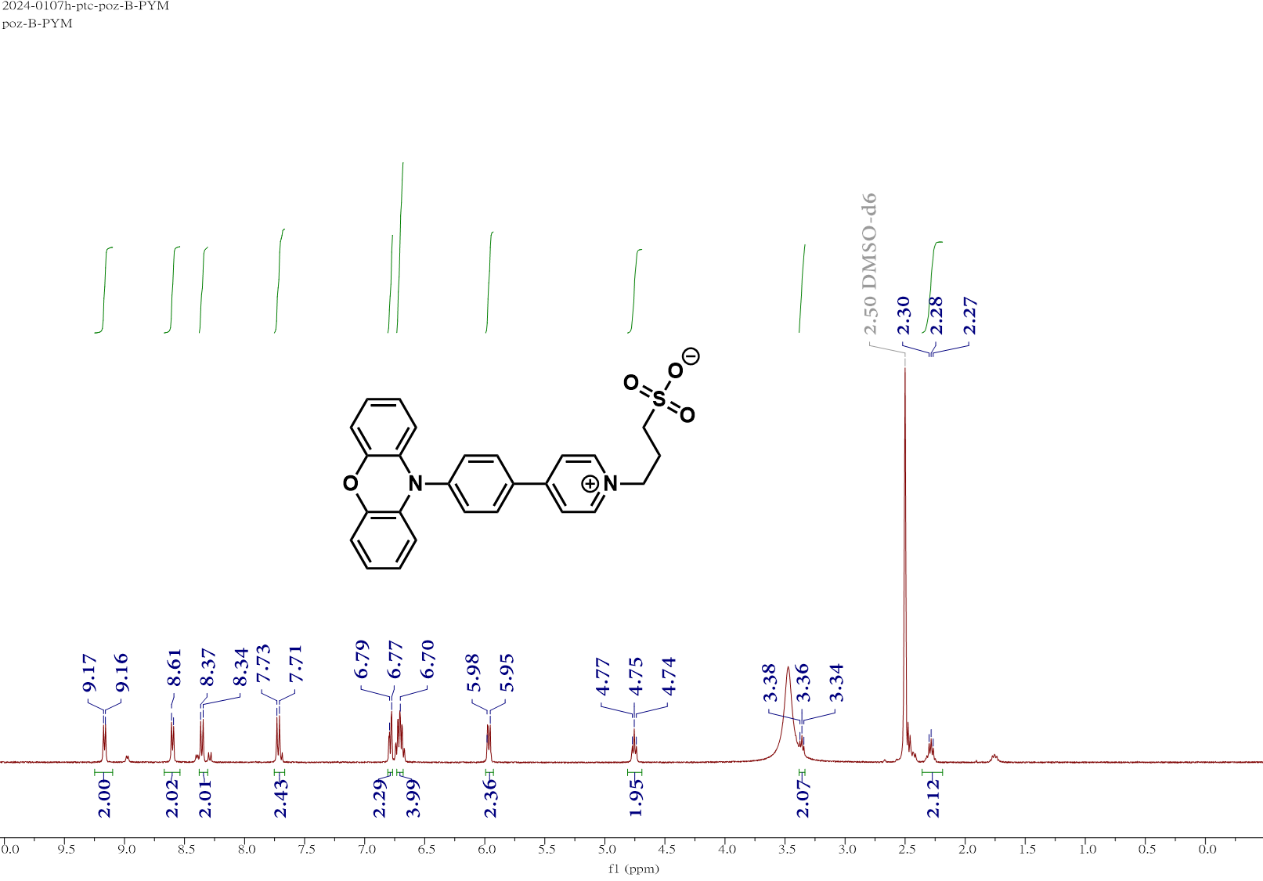


**Figure S19.** ^1^H NMR spectrum of **POZ-B-PY** (DMSO-*d_6_*).


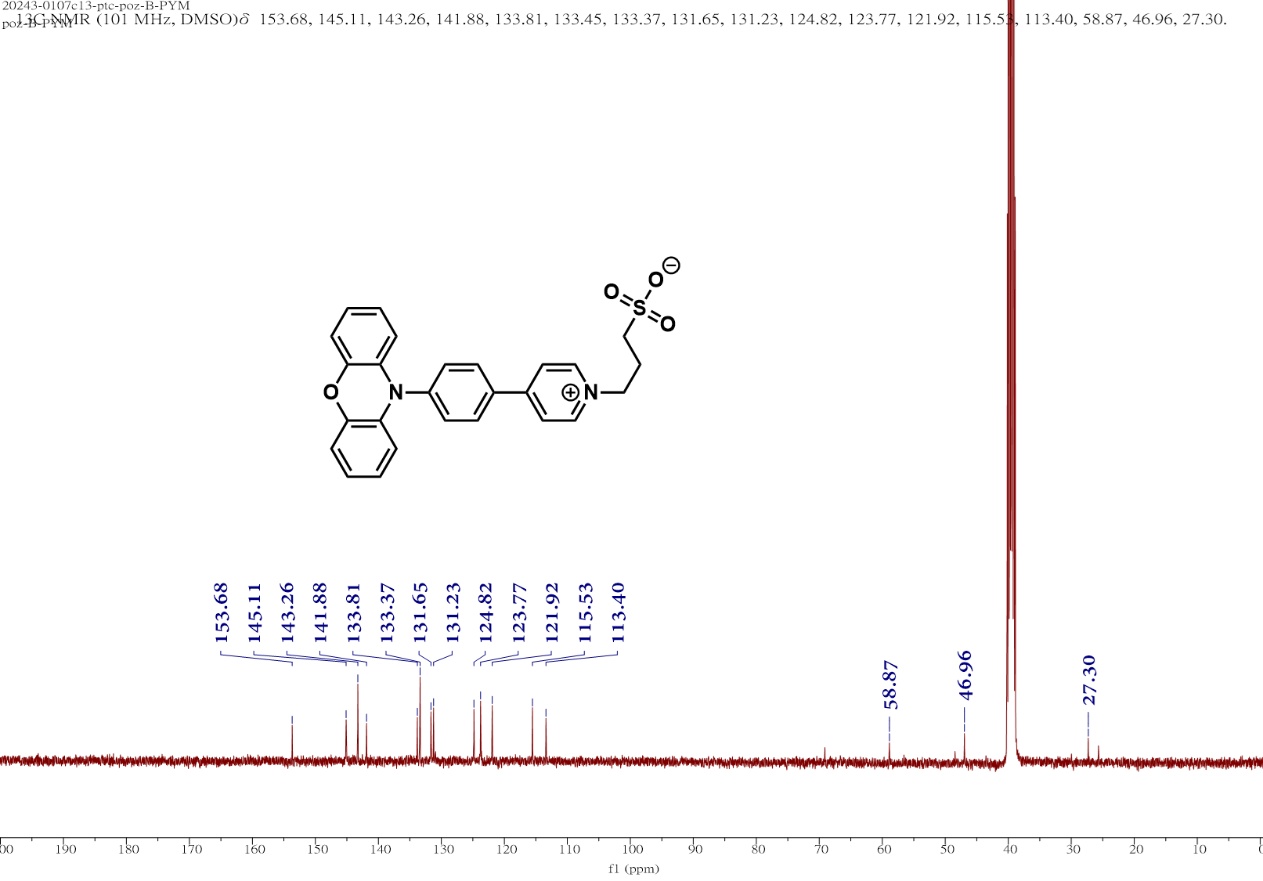


**Figure S20.** ^13^C NMR spectrum of **POZ-B-PY** (DMSO-*d_6_*).

**
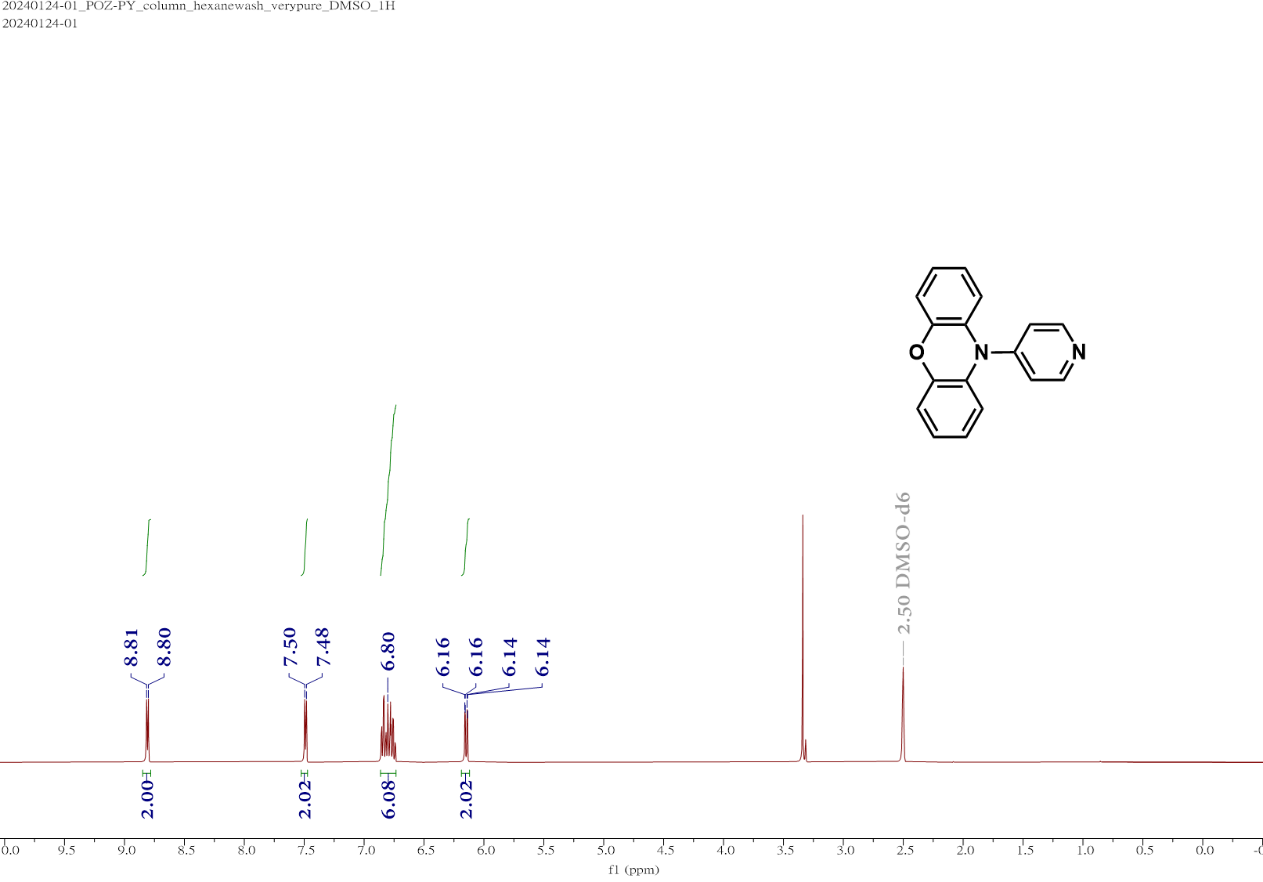
**

**Figure S21.** ^1^H NMR spectrum of **6** (DMSO-*d_6_*).

**
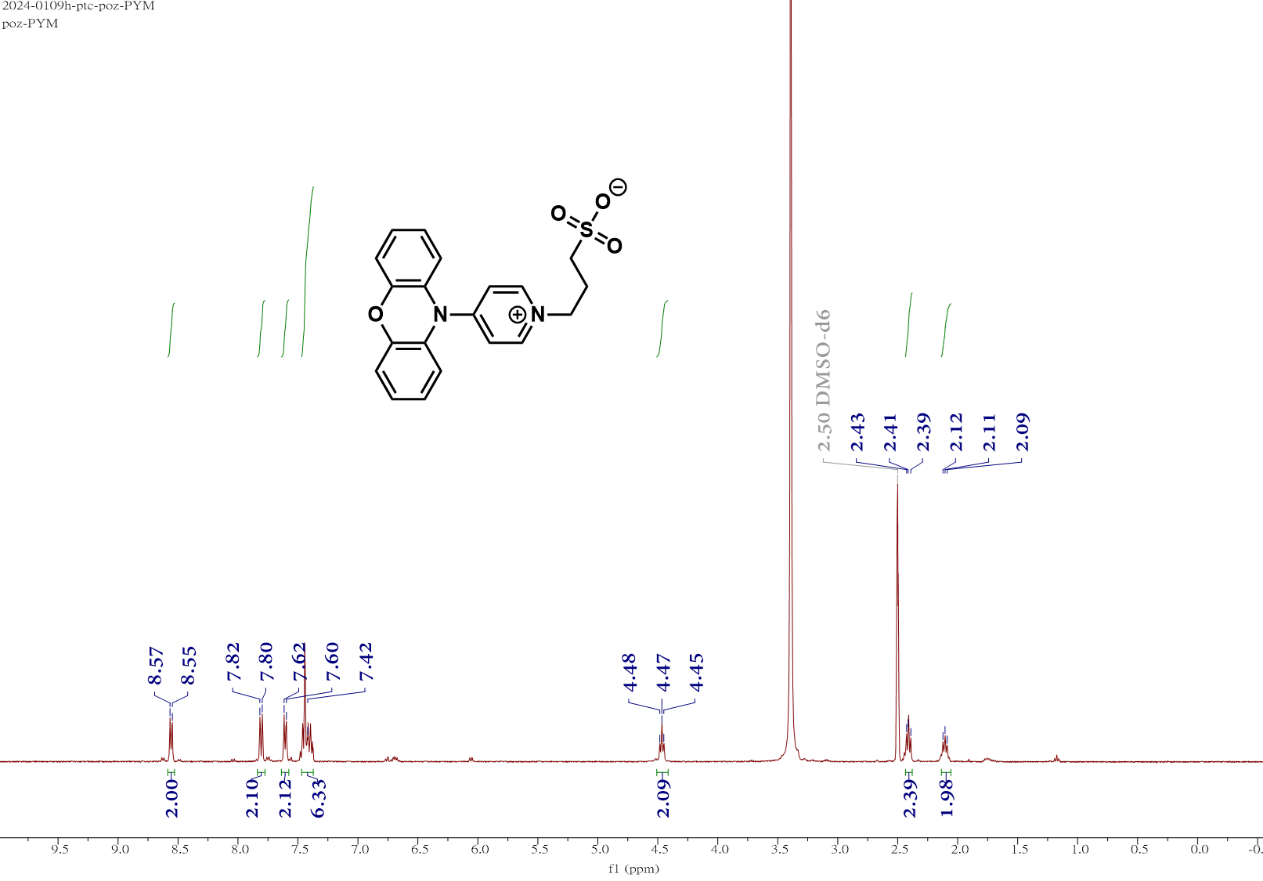
**

*Figure S22*. ^1^H NMR spectrum of **POZ-B-PY** (DMSO-*d_6_*).

**
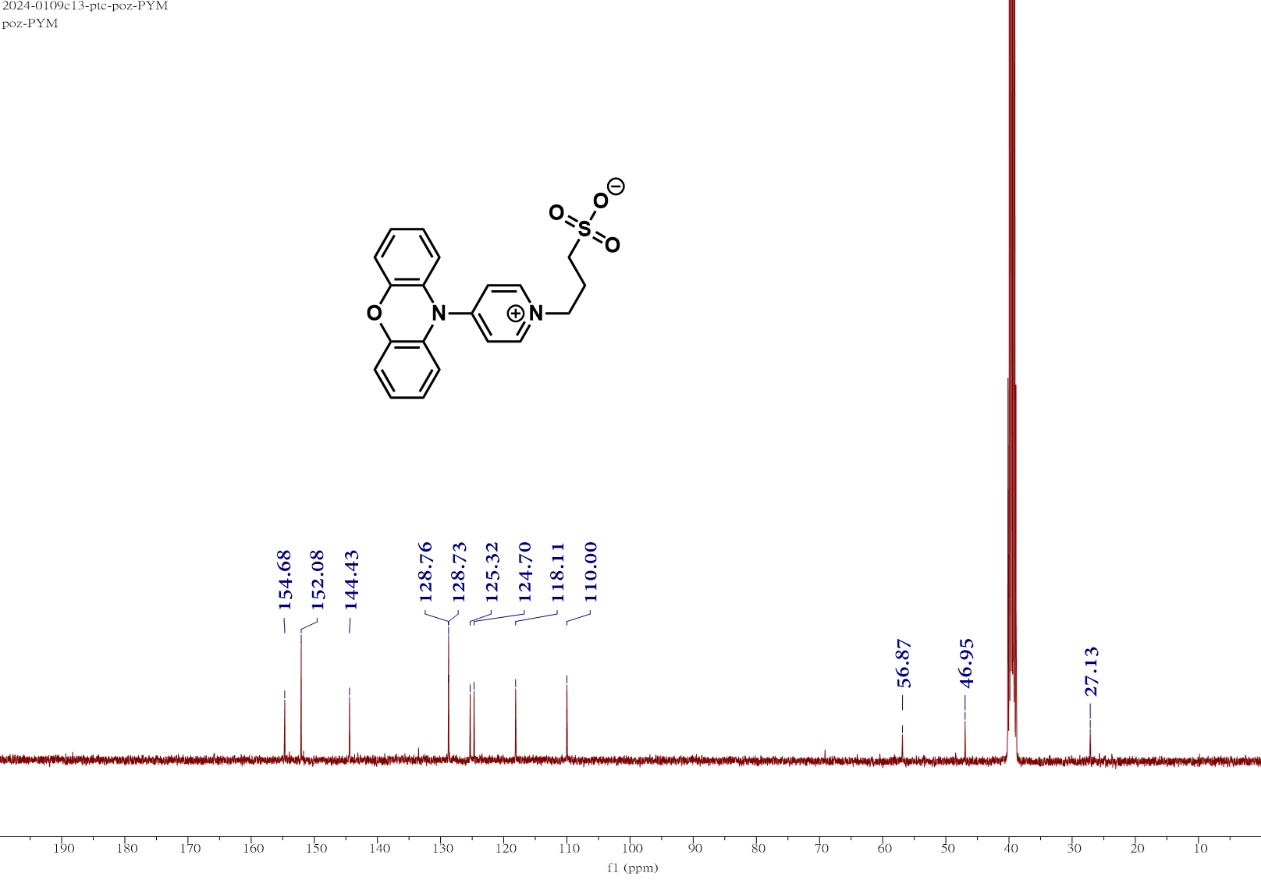
**

**Figure S23.** ^13^C NMR spectrum of **POZ-B-PY** (DMSO-*d_6_*).

**
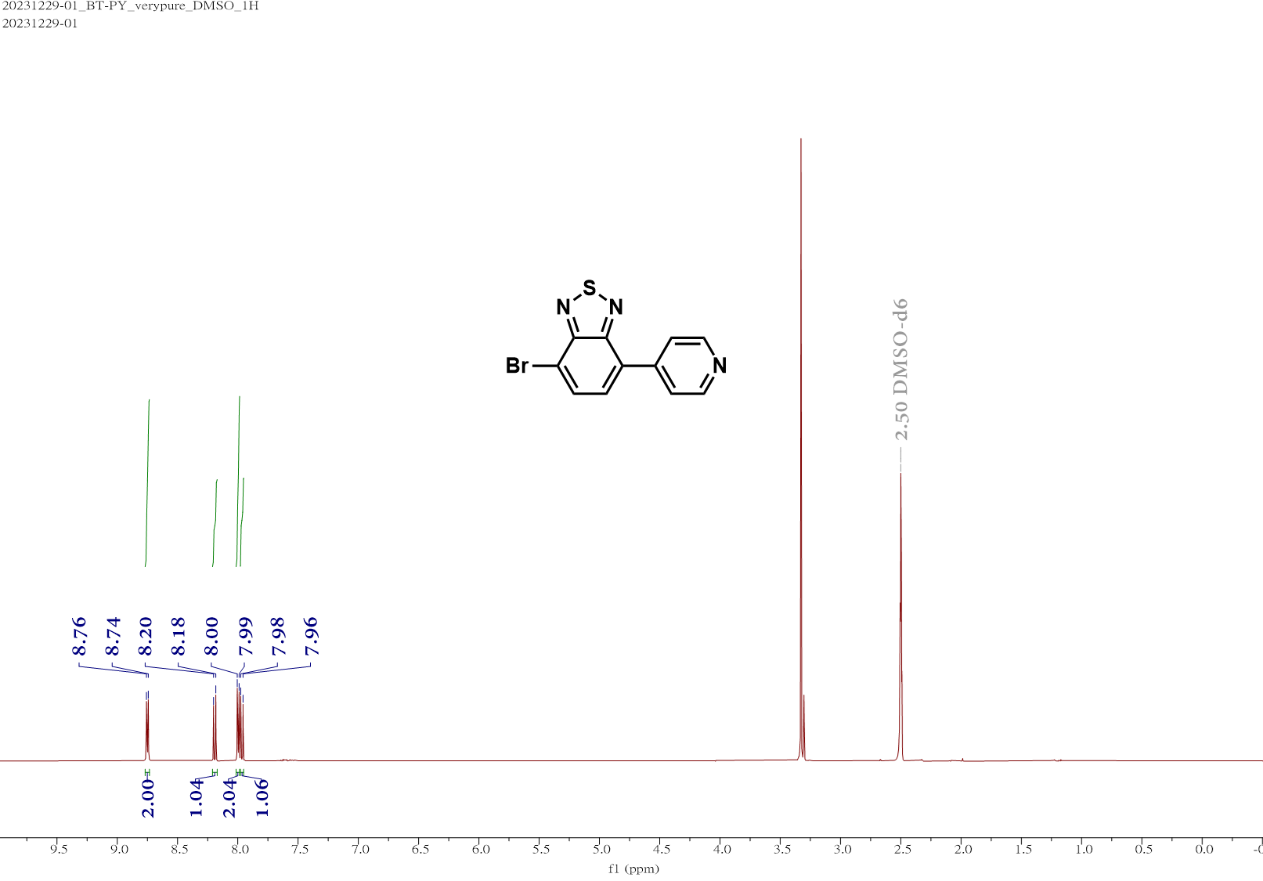
**

**Figure S24.** ^1^H NMR spectrum of **7** (DMSO-*d_6_*).

**
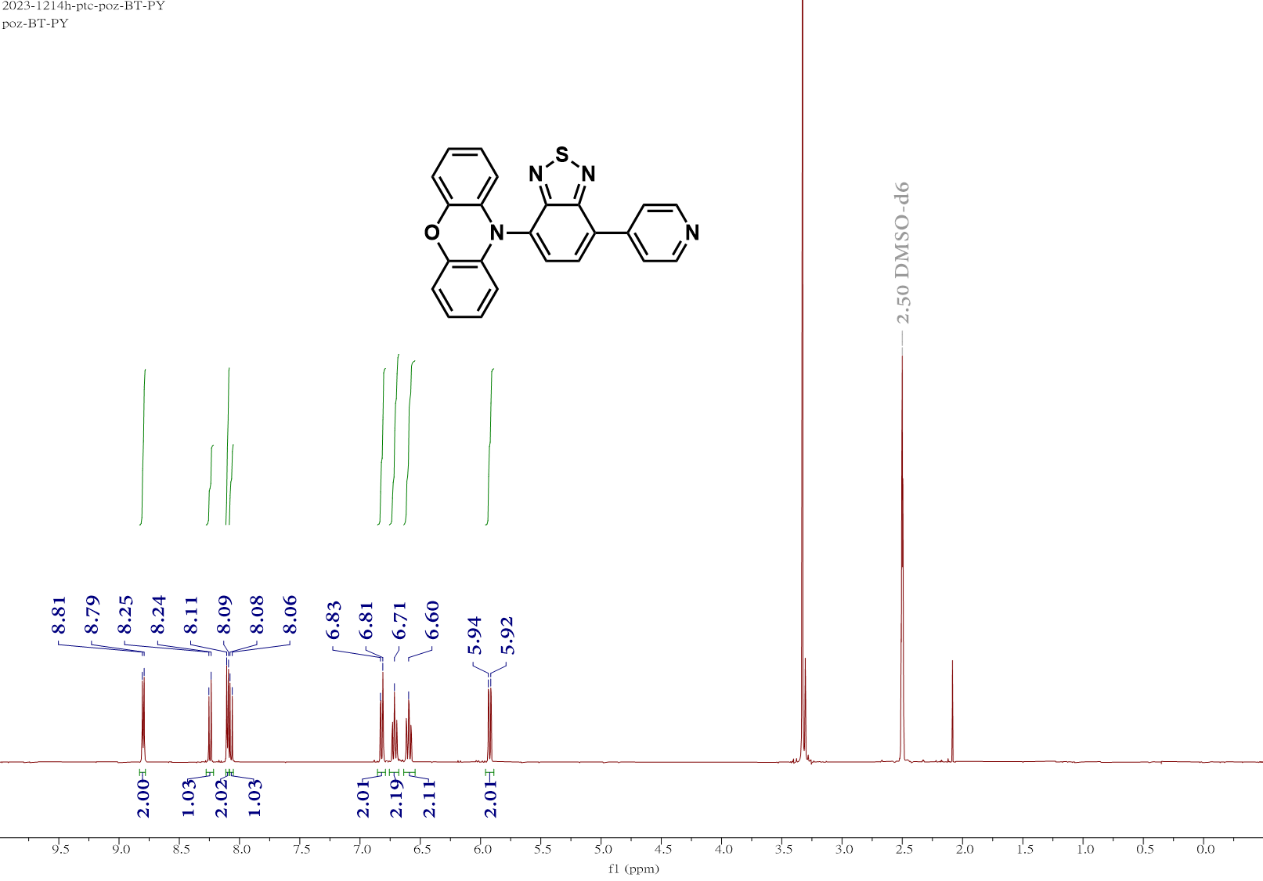
**

**Figure S25.** ^1^H NMR spectrum of **8** (DMSO-*d_6_*).

**
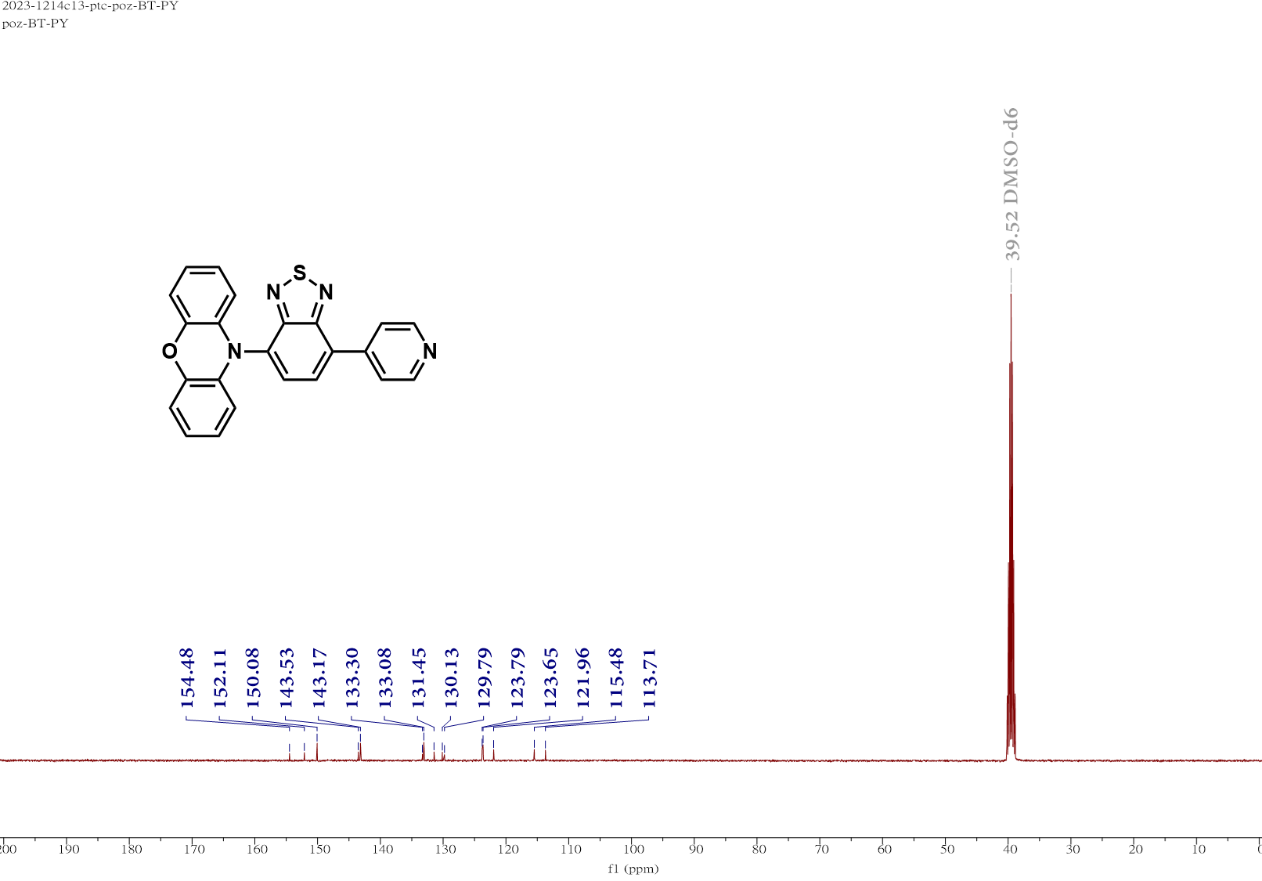
**

**Figure S26.** ^13^C NMR spectrum of **8** (DMSO-*d_6_*).

**
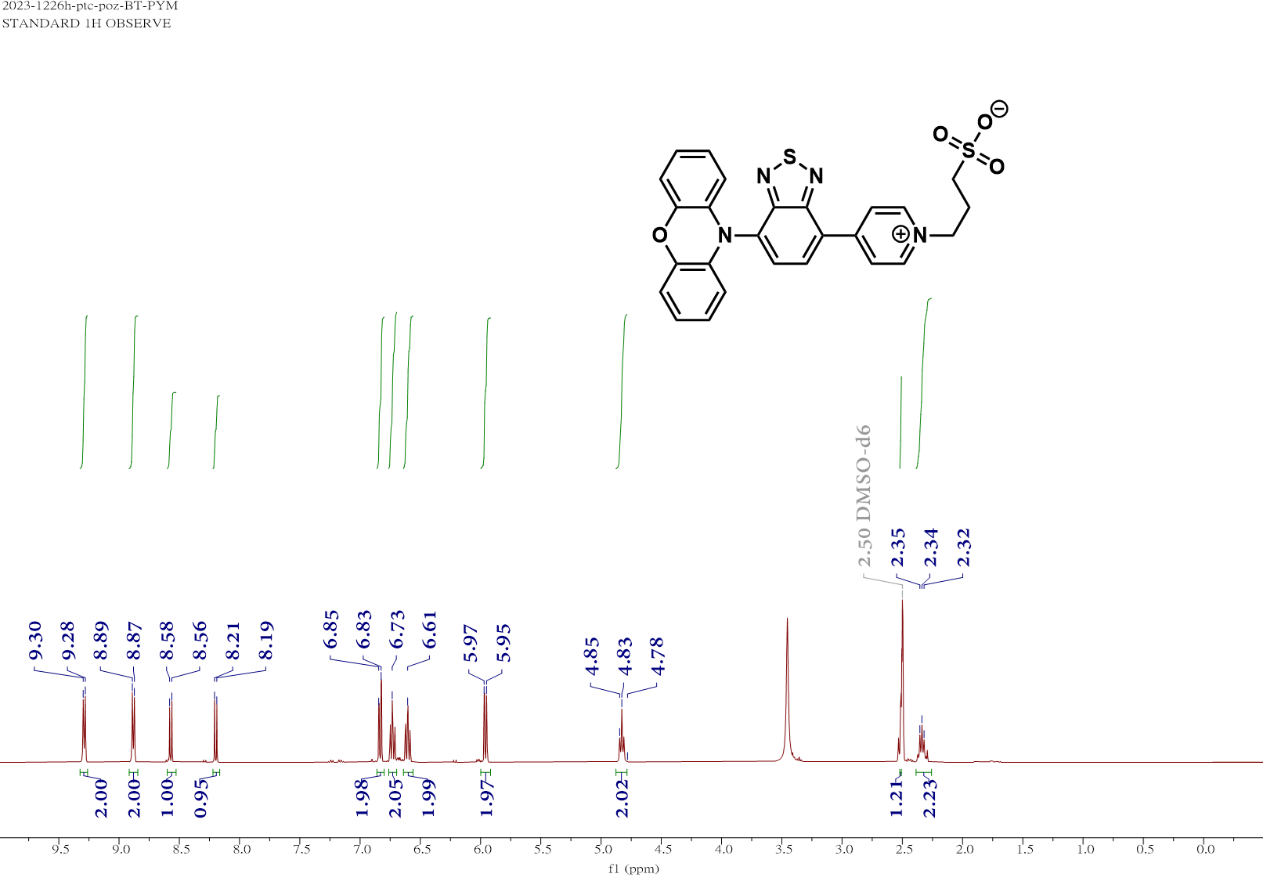
**

**Figure S27.** ^1^H NMR spectrum of **POZ-BT-PY** (DMSO-*d_6_*)

**
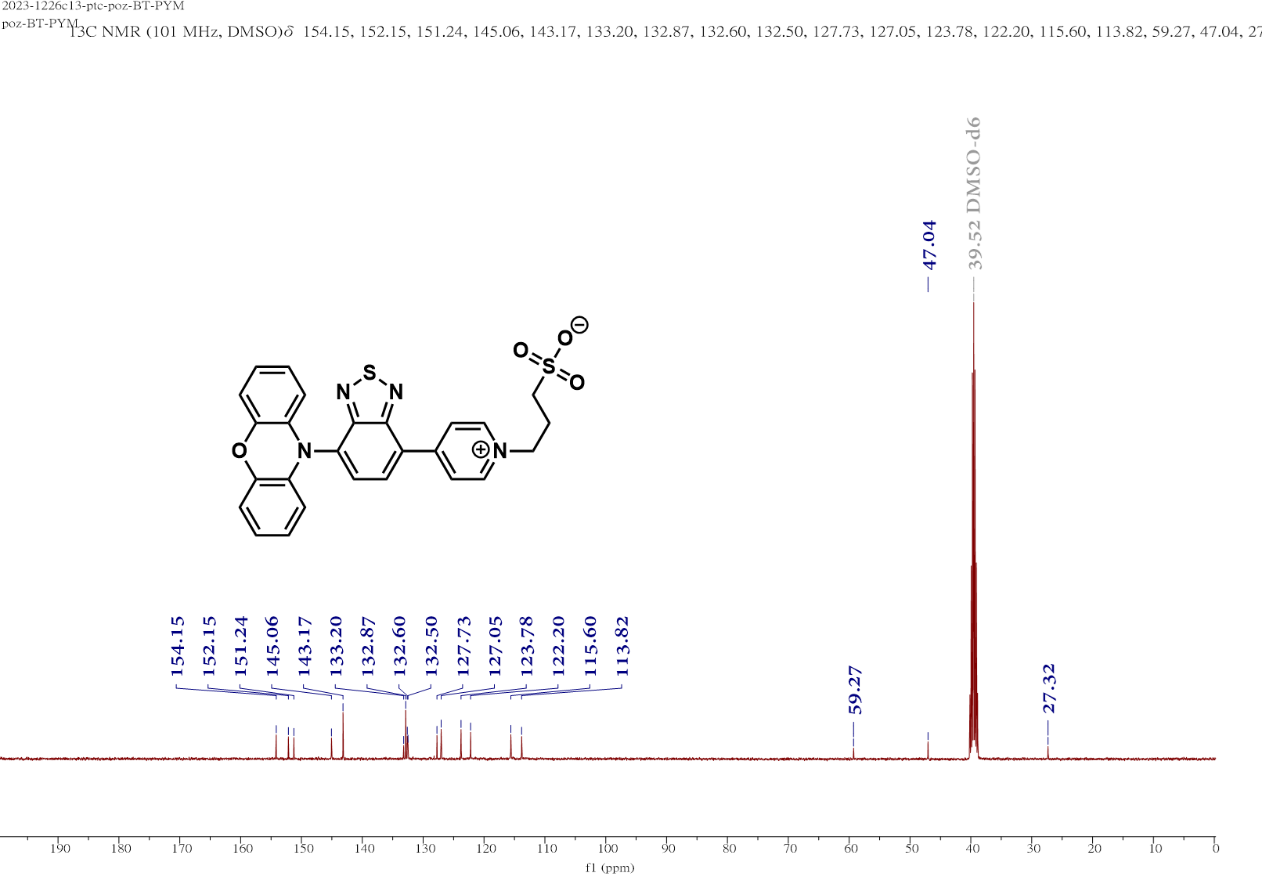
**

**Figure S28.** ^13^C NMR spectrum of **POZ-BT-PY** (DMSO-*d_6_*).

**
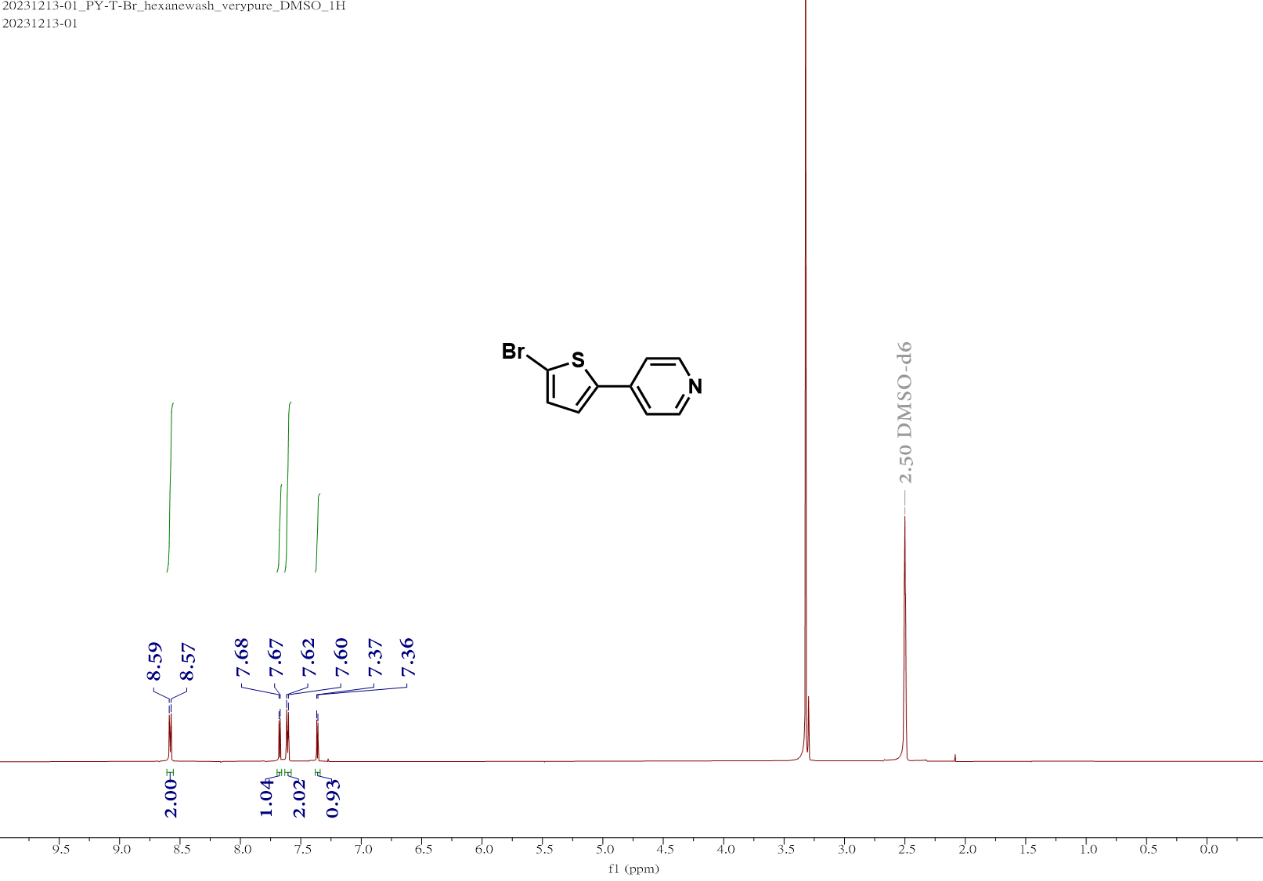
**

**Figure S29.** ^1^H NMR spectrum of **9** (DMSO-*d_6_*)

**
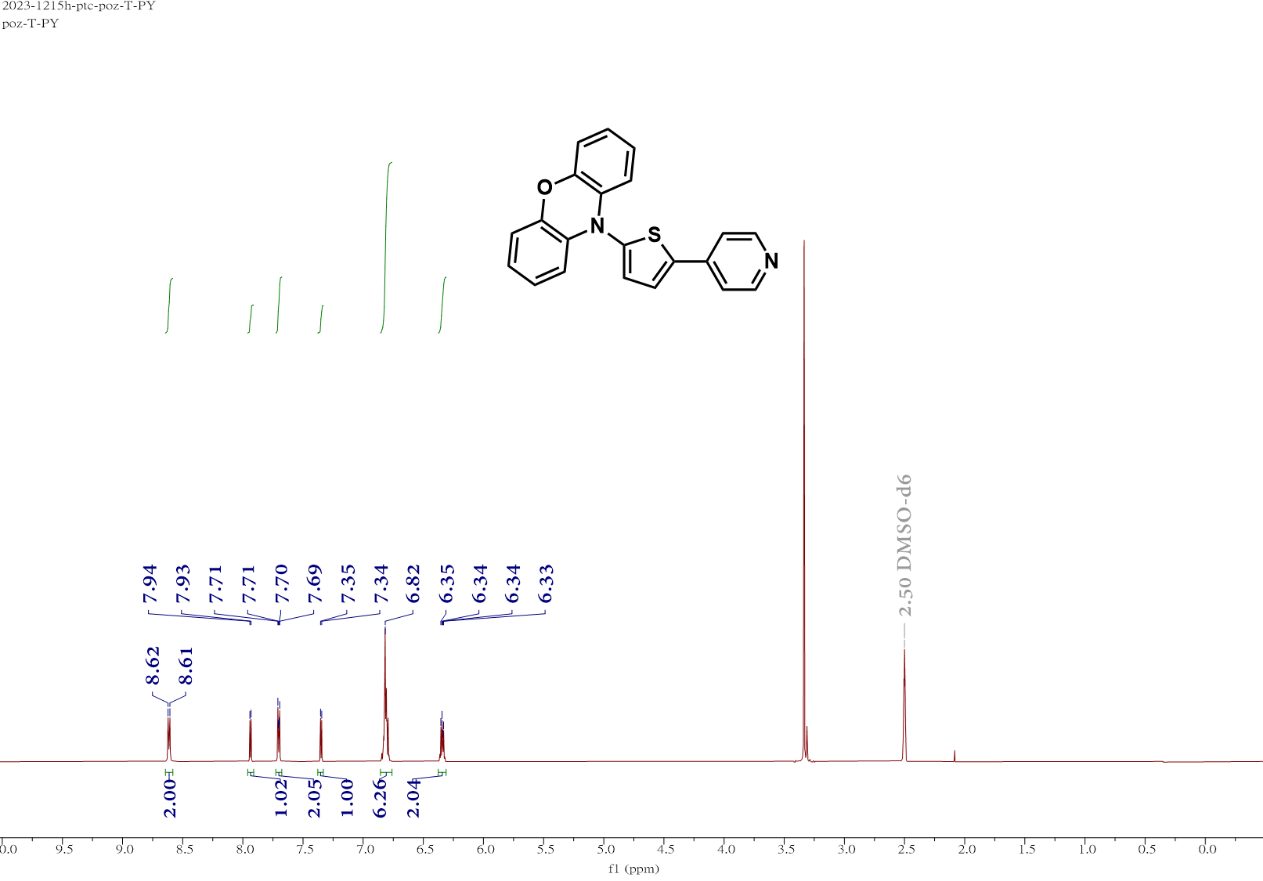
**

**Figure S30.**^1^H NMR spectrum of **10** (DMSO-*d_6_*)

**
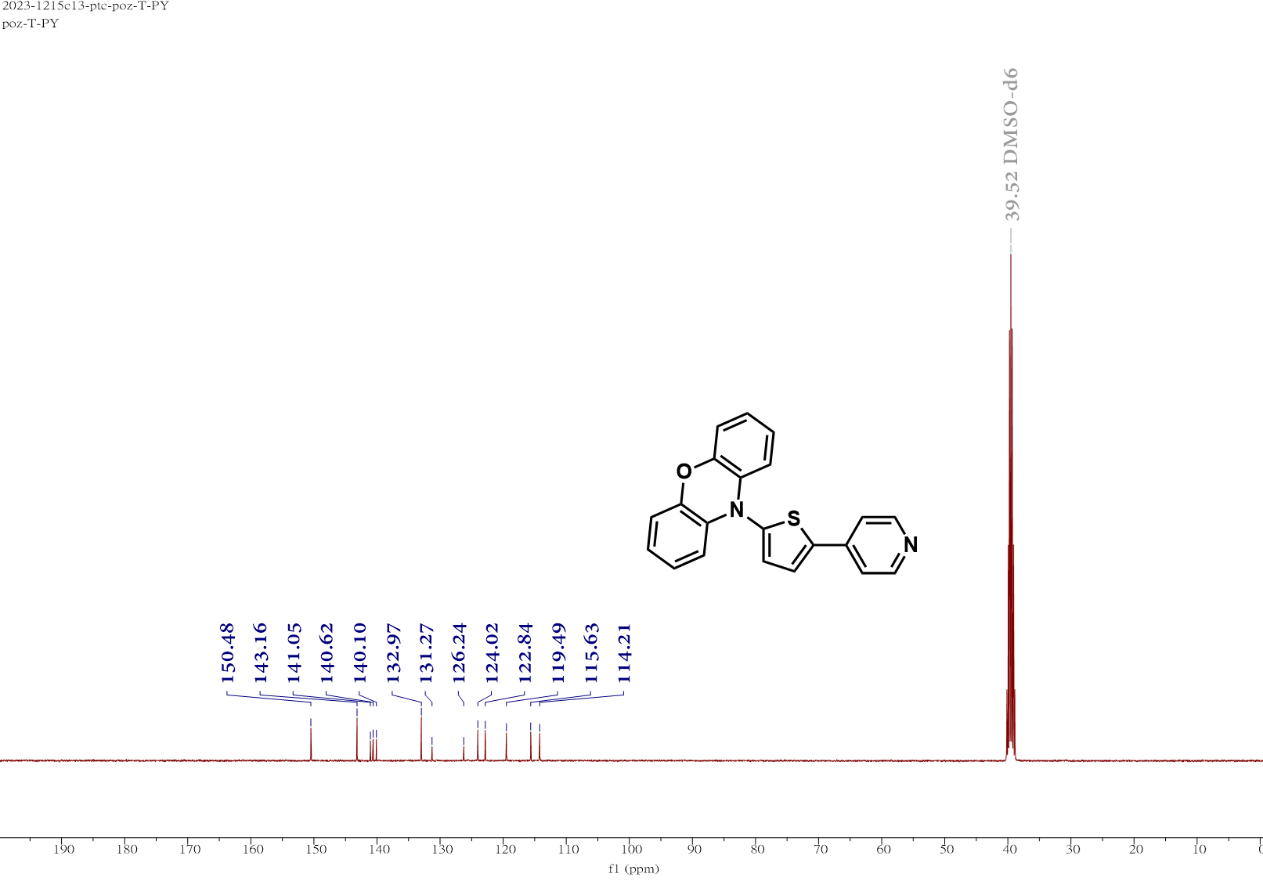
**

**Figure S31.** ^13^C NMR spectrum of **10** (DMSO-*d_6_*).

**
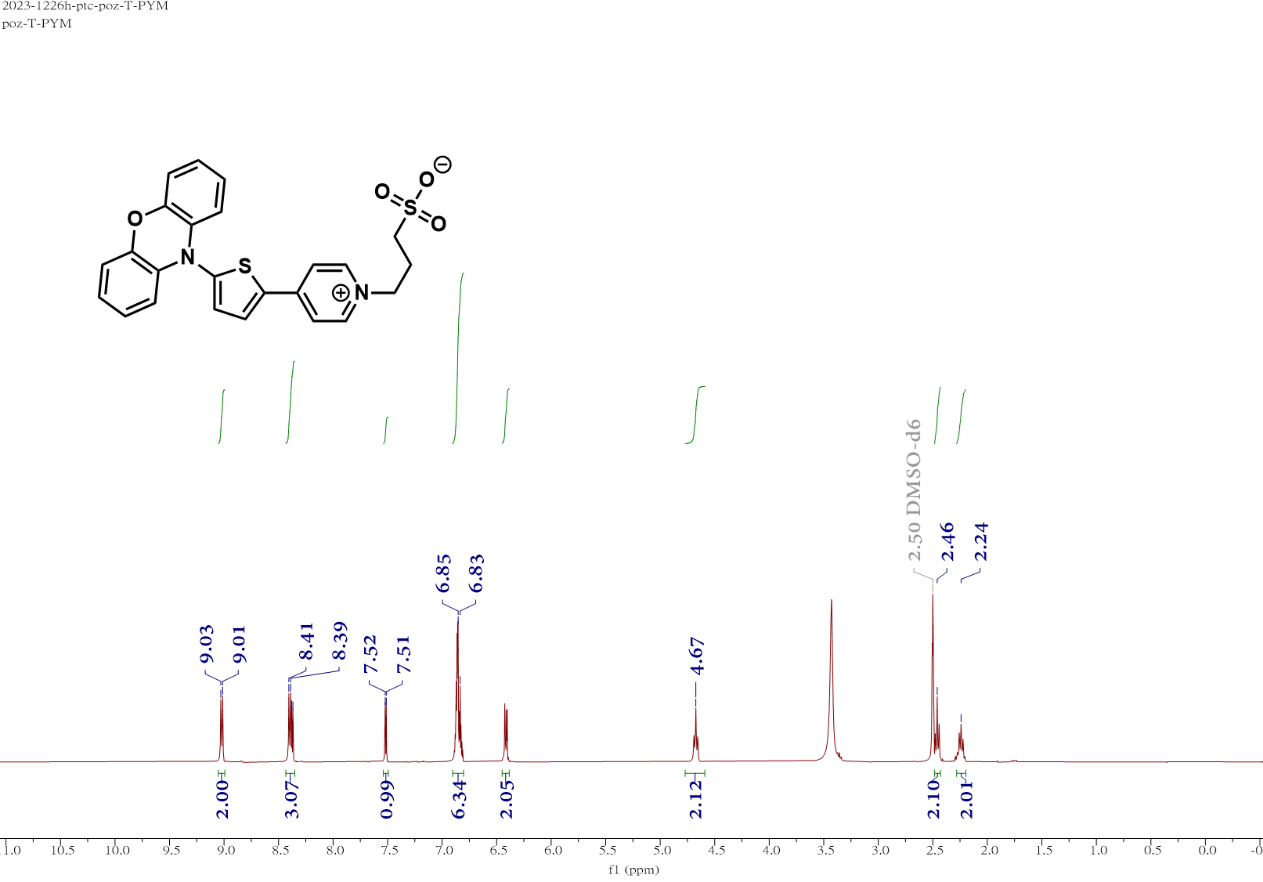
**

**Figure S32.** ^1^H NMR spectrum of **POZ-T-PY** (DMSO-*d_6_*)

**
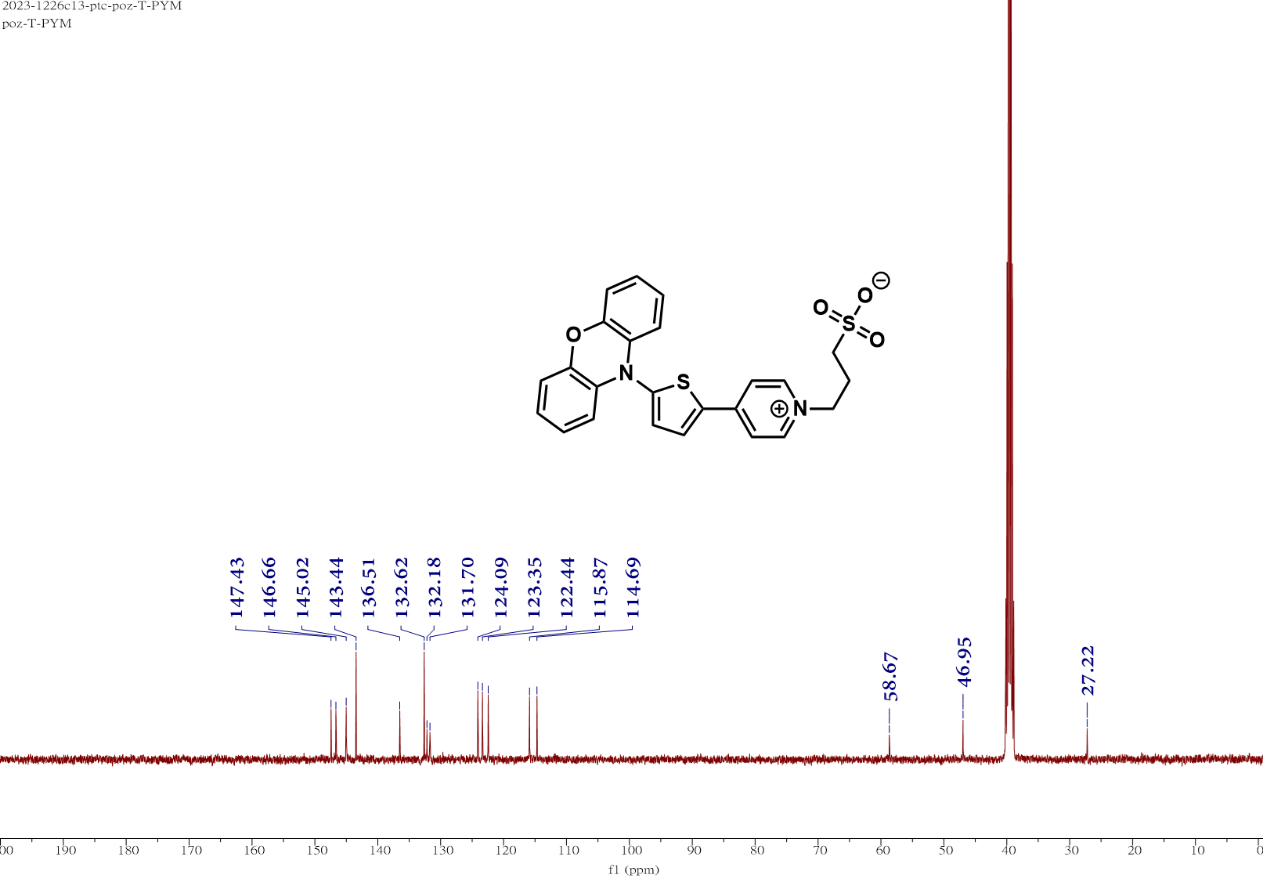
**

**Figure S33.** ^13^C NMR spectrum of **POZ-T-PY** (DMSO-*d_6_*).


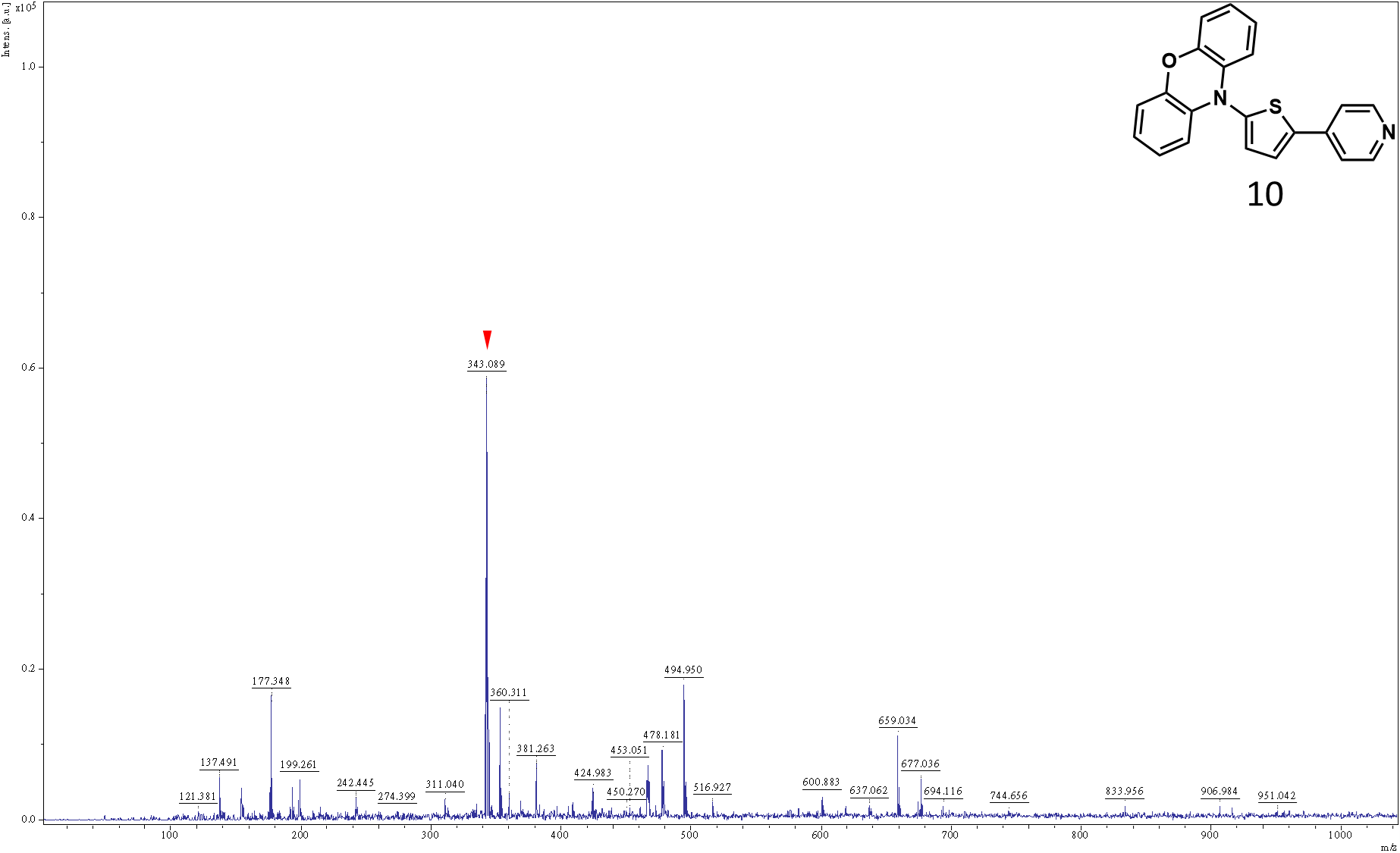


**Figure S34.** Mass Spectra of compound **10**.


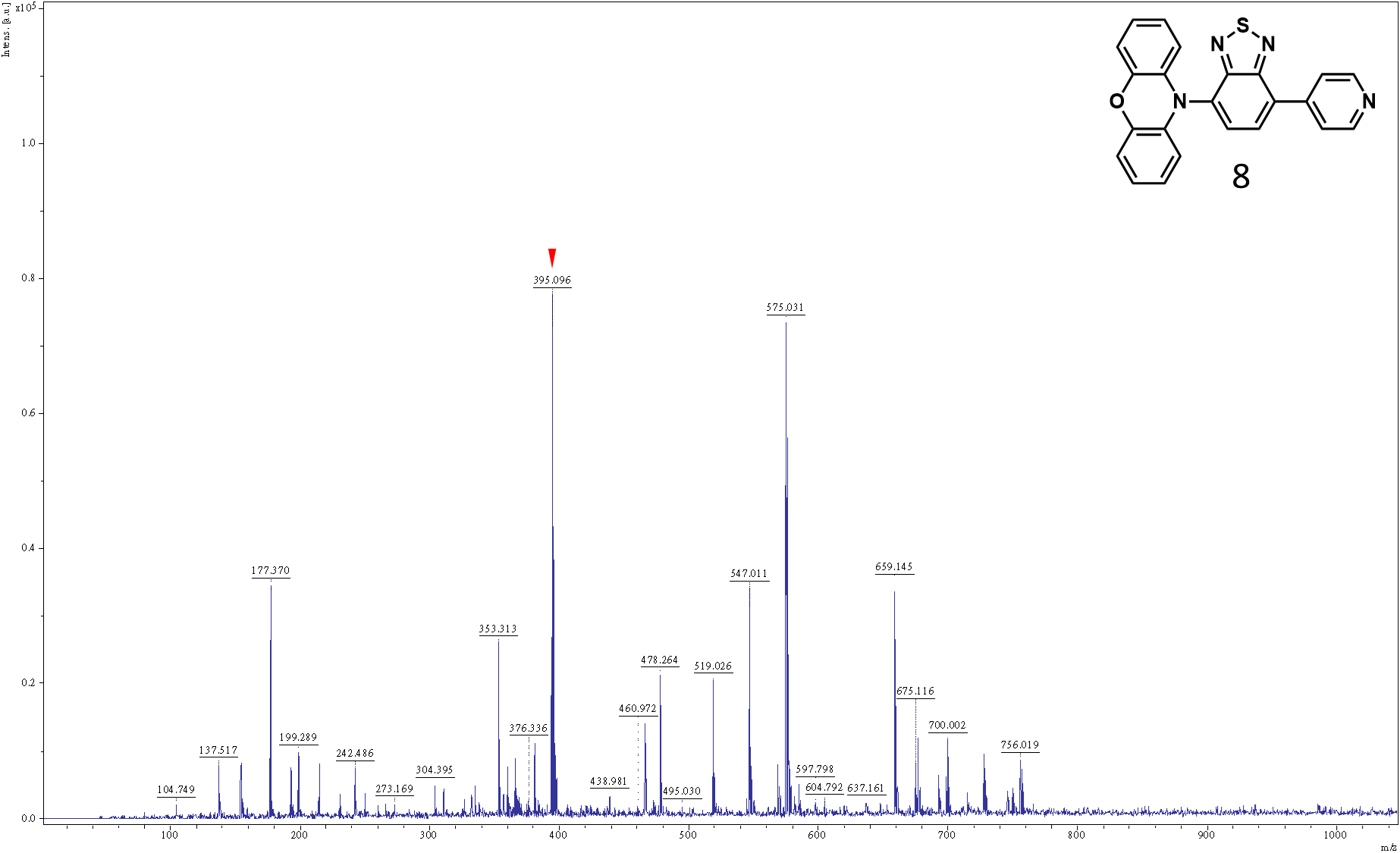


**Figure S35.** Mass Spectra of compound **8**.


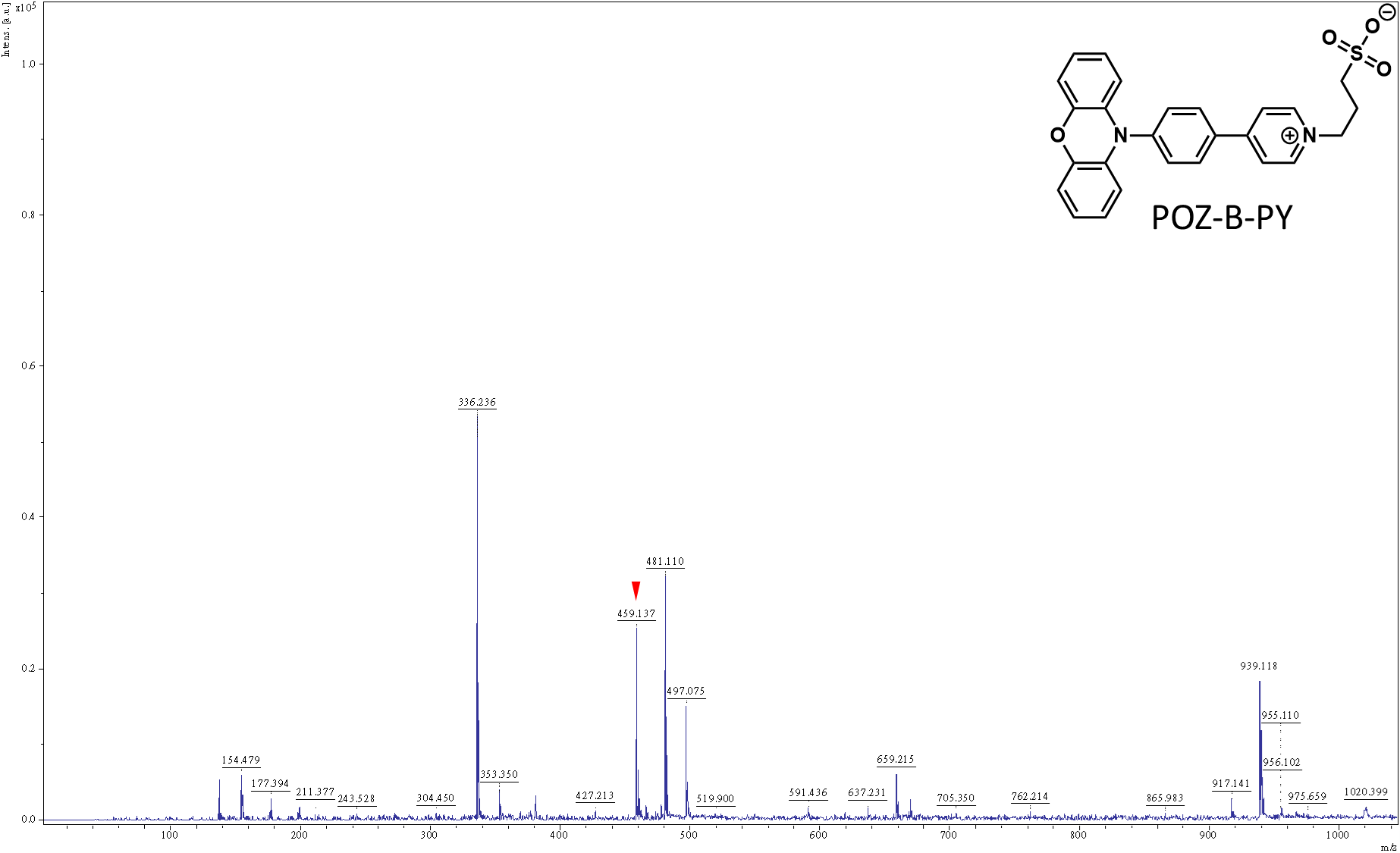


**Figure S36.** Mass Spectra of **POZ-B-PY**.


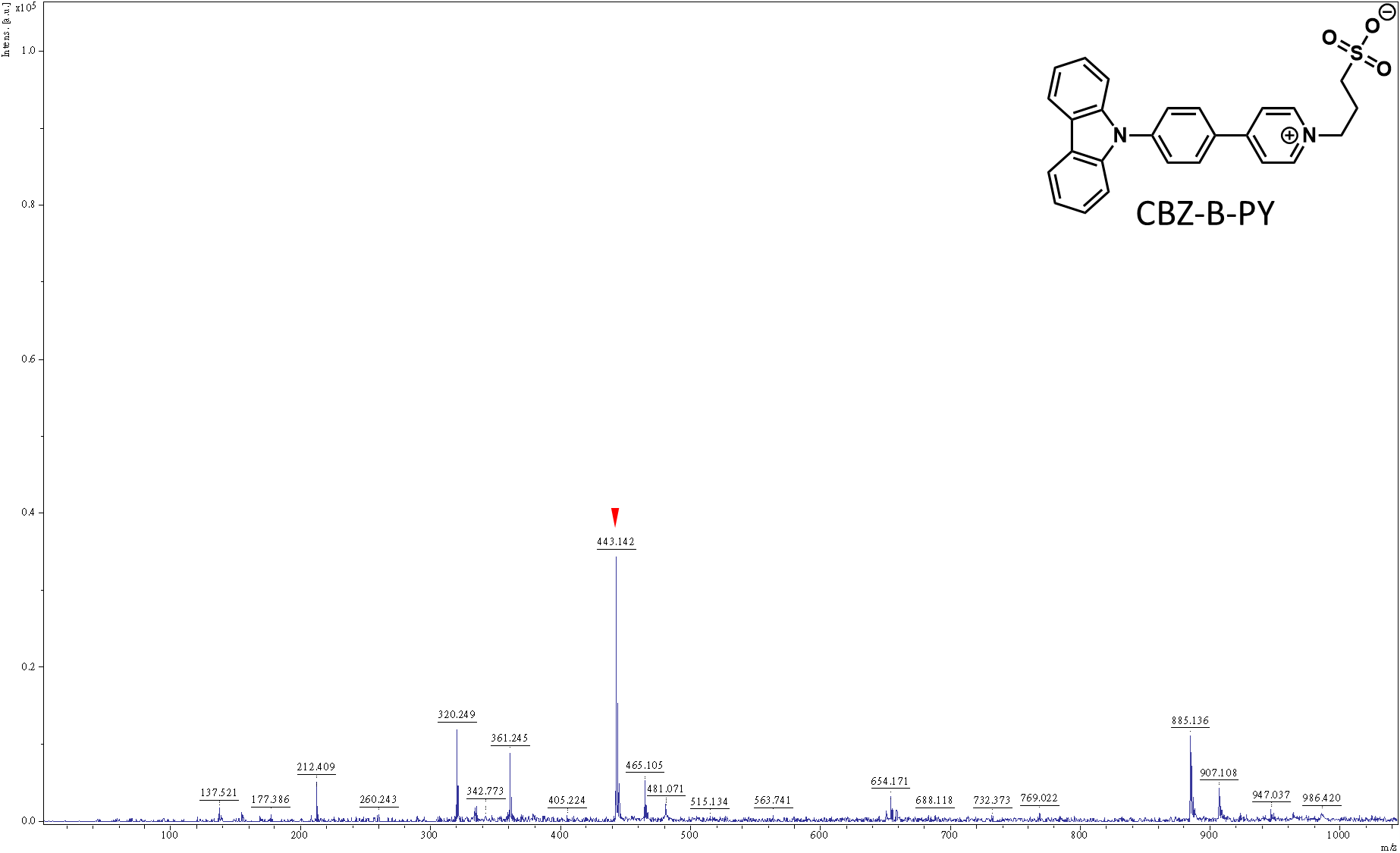


**Figure S37.** Mass Spectra of **CBZ-B-PY**.


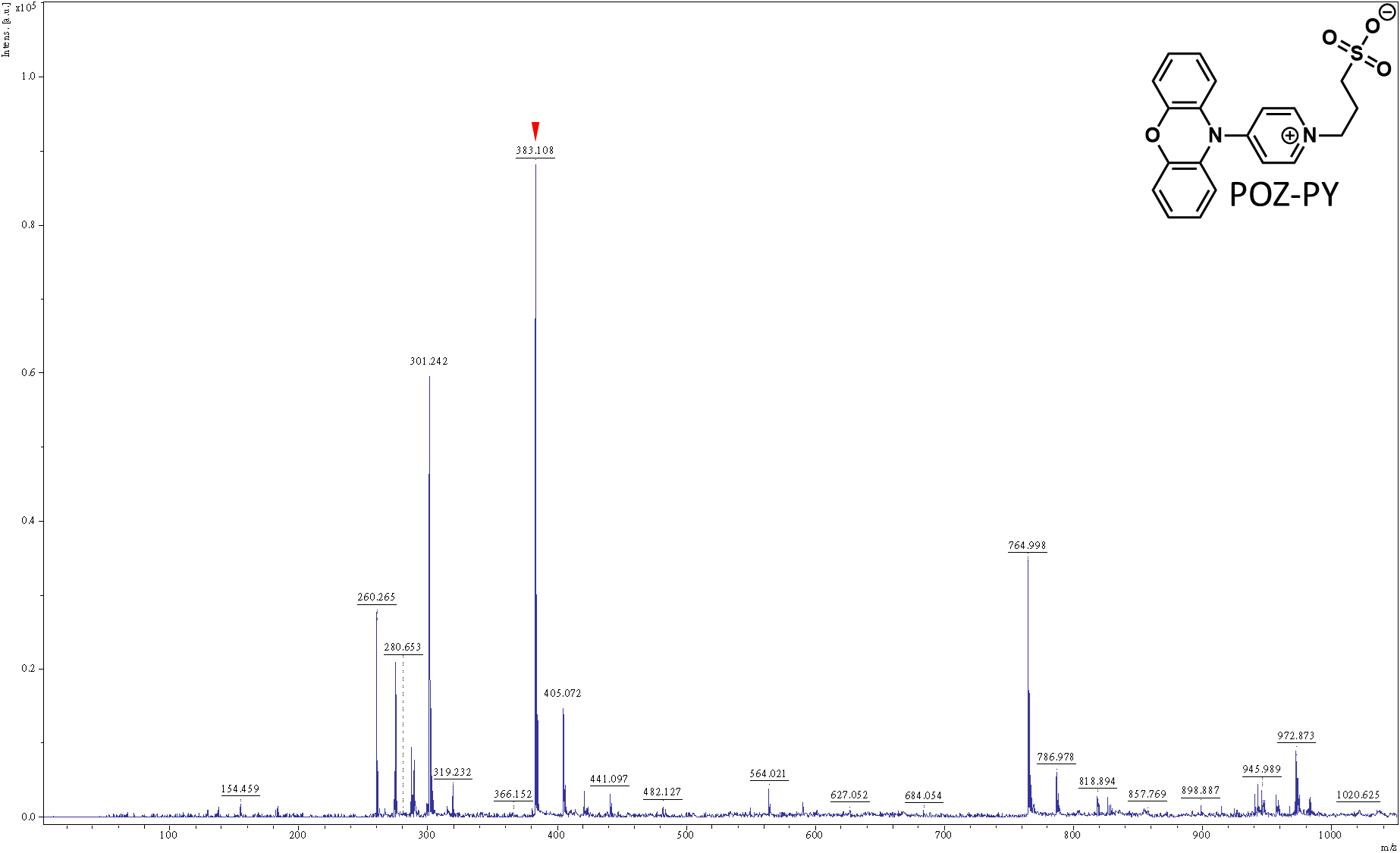


**Figure S38.** Mass Spectra of **POZ- PY**.


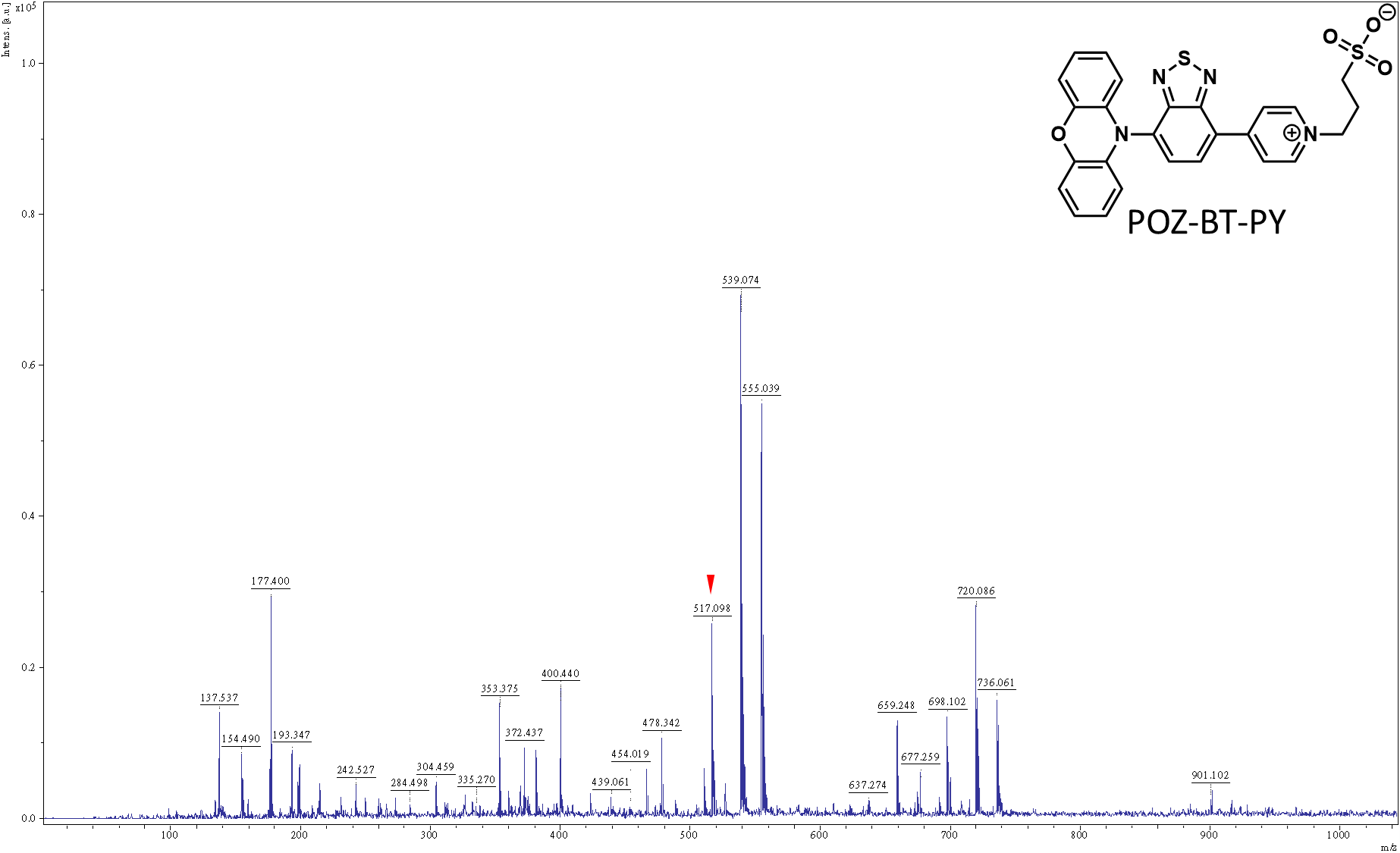


**Figure S39.** Mass Spectra of **POZ-BT-PY**.


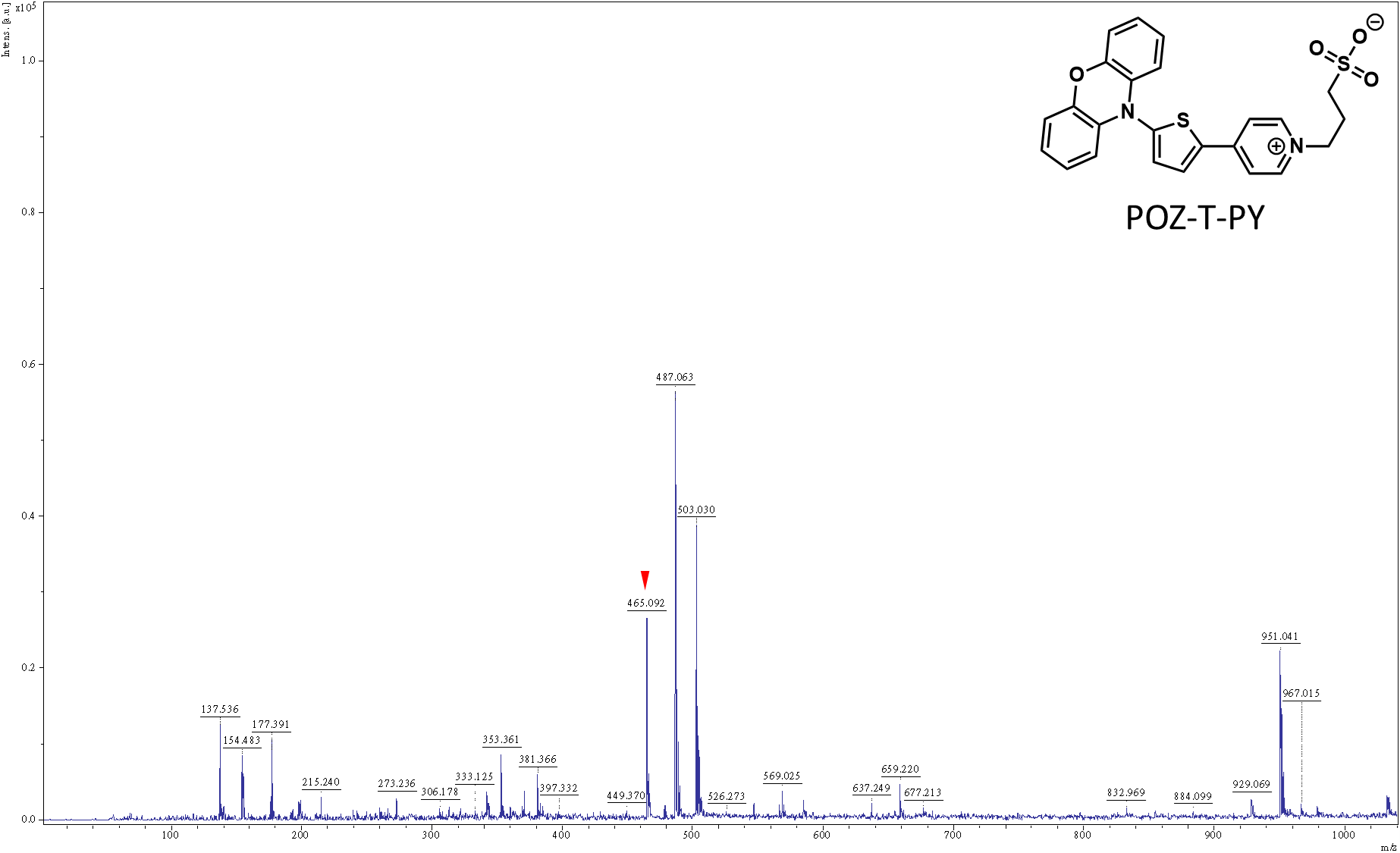


**Figure S40.** Mass Spectra of **POZ-T-PY**.

**References:**

(1) Y.-C. Wei, B.-H. Chen, R.-S. Ye, H.-W. Huang, J.-X. Su, C.-Y. Lin, J. Hodgkiss, L.-Y. Hsu, Y. Chi, K. Chen, C.-H. Lu, S.-D. Yang, P.-T. Chou, *Angew. Chem. Int. Ed.* **2023**, *62*, e202300815.

(2) C.-H. Lu, Y.-J. Tsou, H.-Y. Chen, B.-H. Chen, Y.-C. Cheng, S.-D. Yang, M.-C. Chen, C.-C. Hsu, A. H. Kung, *Optica* **2014**, *1*, 400-406.

(3) C.-H. Lu, W.-H. Wu, S.-H. Kuo, J.-Y. Guo, M.-C. Chen, S.-D. Yang, A. H. Kung, *Opt. Express* **2019**, *27*, 15638-15648.
